# Novel mutations in the kinase domain of BCR-ABL gene causing imatinib resistance in chronic myeloid leukemia patients

**DOI:** 10.1038/s41598-019-38672-x

**Published:** 2019-02-20

**Authors:** Chodimella Chandrasekhar, Pasupuleti Santhosh Kumar, Potukuchi Venkata Gurunadha Krishna Sarma

**Affiliations:** 1 Department of Hematology, Sri Venkateswara Institute of Medical Sciences, Tirupati, 517507 Andhra Pradesh India; 2 Department of Biotechnology, Sri Venkateswara Institute of Medical Sciences, Tirupati, 517507 Andhra Pradesh India

## Abstract

Mutations in the drug binding region of BCR-ABL lead to imatinib resistance during the management of chronic myeloid leukemia (CML). In our study, 62 Philadelphia positive (Ph^+^) CML patients showing conspicuous expression of BCR-ABL gene were treated with imatinib. At the end of 3 months, 21/62 (33.87%) patients did not obtain complete hematological response (CHR) and also showed no significant decrease in BCR-ABL gene expression. In all the imatinib-resistant patients BCR-ABL gene was PCR amplified and sequenced. The sequence analysis showed four novel missense mutations p.(Leu301Ile), p.(Tyr320His), p.(Glu373Asp), p.(Asp381Asn) and six already reported mutations p.(Val256Gly), p.(Thr315Ile), p.(Gly250Glu), p.(Tyr253His), p.(Phe317Leu), p.(Met351Thr) which contributed in the formation of inactive enzyme and also two novel frameshift mutations p.(Glu281*) and p.(Tyr393*), which resulted in truncated protein formation. Further, the structural analysis revealed all these mutations affected P-loop, gatekeeper, catalytic and activation loop domain regions of the enzyme causing poor imatinib binding in the ATP region. The primary intention of the study was to find out the mutations in the BCR-ABL gene causing imatinib resistance. This study highlights the need for BCR-ABL gene sequence analysis to detect the mutations in CML patients in order to properly guide the therapy.

## Introduction

Chronic myeloid leukemia (CML) is a clonal myeloproliferative disorder of primitive hematopoietic progenitor cells^1^. The BCR-ABL tyrosine kinase produced by the t(9;22)(q34;q11) translocation fuses the parts of the q arm of chromosome 9 to the q arm of chromosome 22, creating a hybrid BCR-ABL gene, also known as the Philadelphia chromosome and initiates the event of CML^2–7^. The abl is a proto-oncogene that encodes a protein tyrosine kinase involved in a variety of cellular processes, including cell division, adhesion, differentiation, DNA damage response, and apoptosis. This BCR-ABL gene is ubiquitously expressed and is regulated by cyclin-dependent kinase 1 (CDK1) or cell division cycle protein 2 homolog (CDC2)-mediated by phosphorylation and therefore, the mutations in this gene cause loss of regulation of DNA damage response and apoptosis which are some of the strong contributory reasons for cancerous condition in CML patients^6,8–11^.

The crystal structure of the abl N-terminal regulatory region with its Src homology 3 (SH3) and Src homology 2 (SH2) domains is important for the regulation of its activity *in vivo*^5^. The SH3 domain is essentially a β sandwich, which consists of five β strands (βa-βe) and β hairpin-like turn (the ab loop)^9^. The SH2 domain is composed of the central antiparallel β sheet that is flanked on one side by an α helix (αA) and on the other side by a second alpha helix (αB) and a small β hairpin^10^. Along with the SH3 and SH2 domains, phosphate binding loop, imatinib binding region and catalytic site are present in the ABL kinase domain.

Imatinib is used as a frontline therapy for CML and imatinib is a selective inhibitor of tyrosine kinase that binds to the ATP-binding site of BCR-ABL gene. In CML patients, mutations in the kinase domain of BCR-ABL gene result in the imatinib resistance^8,12^. The mutations in the BCR-ABL gene interfere with critical hydrogen bonds that form between the kinase domain of BCR-ABL and inhibitor molecule, thus impairing the ability of the BCR-ABL protein to bind to the ATP-binding site of inhibitor molecule.

Hence the present study was aimed at identifying the mutations in the kinase domain of BCR-ABL gene in the clinically proven CML patients. The kinase domain of BCR-ABL gene was PCR-amplified and sequenced. The expression of BCR-ABL gene was measured through quantitative real time-PCR. Further, *in silico* analysis was carried out to correlate the structural and functional analysis of the BCR-ABL gene.

## Results

### Clinical characteristics

**Figure 1:**
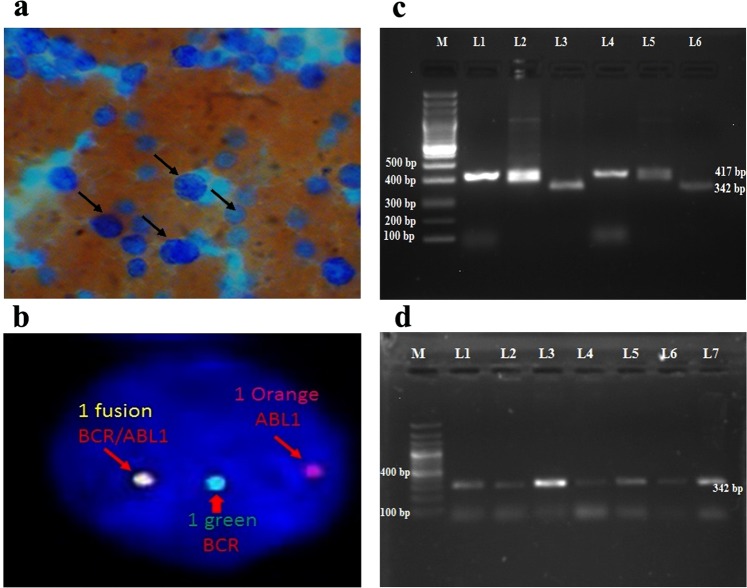
(**a**) Peripheral smear of CML patient by using Leishman staining showed large granulocytic cells. (**b**) The BCR/ABL1:t(9;22) FISH probe (Vysis) in the Interphase cell showing 1 fusion, 1 Orange and 1 Green signals indicating the BCR/ABL: Ph-positive (9q deletion variant) status. (**c**) Reverse transcriptase-polymerase chain reaction of BCR-ABL transcript variants in 1.5% agarose gel electrophoresis. Lane M: 100 bp ladder, Lane L1, L2, L4 and L5: CML patients samples showing b3a2 variant with 417 bp size, Lane L3 and L6: CML patients samples showing b2a2 variant with 342 bp size. (**d**) The 1.5% agarose gel showing Lane M: 100 bp ladder, Lane L1-L7: CML patients samples showing b2a2 variant with 342 bp size.

The demographic profile, disease characteristics of 62 CML patients studied were shown in Table 1. The CML was marginally more common in males (n = 39, 62.9%) than in females (n = 23, 37.09%) (Table 1). The morphological identification of CML was done on peripheral smear and bone marrow histopathology and different phases were recognized (Fig. 1a). With respect to clinical phase, patients were more in chronic phase (CP) (n = 35, 56.45%) than in accelerated phase (AP) (21, 33.87%) or blast crisis (BC) (6, 9.67%). Baseline hematological parameters were used to calculate prognostic scores such as Sokal score as indicated in Table 1. At the time of diagnosis, 46.77% of patients were with high baseline Sokal scores while 35.48% and 17.74% were with intermediate and low scores respectively (Table 1).

**Table 1:** Baseline clinical characteristics of all CML patients (n = 62) at diagnosis.

**No**. **of patients**	62
**Males**, **No**.	39
**Females**, **No**.	23
**Age (years): median**	53.73
**WBC (x 10** ^**9**^ **/l): median**	59.2
**PLTS (x 10** ^**9**^ **/l): median**	301.5
**Hb (g/dl): median**	11.6
**Splenomegaly: median**	05.8
**Sokal risk: No**.	
Low	11
Intermediate	22
High	29
**Hematological response at 3 months after imatinib initiation**	
CHR	36 (58.06%)
PHR	05 (8.06%)
No HR	21 (33.87%)

All the CML patients have put on imatinib treatment and the dosage was as follows: for CP patients it was 400 mg/day and for AP/BC it was 600 mg/day. The dosage of imatinib, however, was increased appropriately in patients who did not achieve the adequate anticipated responses in terms of haematological response etc. The characterization of resistance was based on the European LeukemiaNet 2009 guidelines^13^. At the end of 3 months treatment, 33.87% (21/62) patients did not achieve CHR and failed to respond to the imatinib treatment while 05 (8.06%) patients showed partial hematological response (PHR). All the other 36 (58.06%) patients showed CHR and responded well to imatinib (Table 1). Interestingly, out of 21 resistant CML patients, 61.90% (13/21 patients) were in CP, of which 61.52% (8/13 patients) were with high Sokal scores and the rest 38.46% (5/13) were with intermediate Sokal score (Tables 2 and 3). Also, 19.04% (4/21) of the resistant patients were in AP, 19.04% (4/21) were in BC and all these patients had high Sokal scores (Tables 2 and 3). In our study, only 41/62 CML patients were willing for cytogenetic and molecular studies and the remaining 21 patients declined to participate.

**Table 2:** Sokal scores of 21/62 resistant patients.

Sokal score in (n = 21) resistant patients	CP (n = 13)	AP (n = 4)	BC (n = 4)
Low	0	0	0
Intermediate	5	0	0
High	8	4	4

**Table 3:** Different stages of CML patients with transcript variants.

	Resistance			Sensitive		
	CP	AP	BC	CP	AP	BC
b3a2	8	2	2	8	0	0
b2a2	5	2	2	4	7	1

### Fluorescence ***in situ*** hybridization (FISH) analysis

The FISH analysis was performed on all the CML patients before imatinib treatment using dual color locus BCR-ABL gene-specific probes. The BCR-ABL signal pattern in 24.39% of the patients showed, 3 green 3 red (3G3R) dual fusion and 75.60% of the patients showed 1 fusion 1 orange 1 green (1F1O1G) at t(9;22)(q34;q11) translocation (Philadelphia positive) (Fig. 1b).

### Qualitative analysis of BCR-ABL fusion transcripts

The BCR-ABL fusion transcripts (b3a2 and b2a2) were analyzed in 41/62 CML patient samples using qualitative reverse transcriptase polymerase chain reaction (RT-PCR). Among 41 patients 25 (60.97%) patients showed the presence of b3a2 transcript variant with a 417 base pair PCR product (Fig. 1c) and 16 (39.02%) patients showed the presence of b2a2 transcript variant, recognized as a 342 base pair PCR product (Fig. 1d). From the database of all CML patients, the Sokal score was calculated at the time of diagnosis and correlated with the b3a2, b2a2 transcript variants. It was noted that more patients from b3a2 transcript variant group were presenting with high Sokal scores 26.82% (11/41), than those of b2a2 transcript variant group 9.75% (4/41) (Table 4).

**Table 4:** Clinical features of CML patients with transcript variants.

	b3a2	b2a2
**No**. **of patients**	25	16
**Males**, **No**.	15	9
**Females**, **No**.	10	7
**Age (years): median**	53.73	52.4
**WBC (x 109/l): median**	59.2	45.3
**PLTS (x 109/l): median**	301.5	290.7
**Hb (g/dl): median**	11.6	10.3
**Splenomegaly: median**	5.8	4
**Sokal risk: No**.		
**Low**	6	5
**Intermediate**	8	7
**High**	11	4
**Hematological response at 3 months after imatinib initiation**		
CHR	8 (19.51%)	12 (29.26%)
PHR	2 (4.87%)	3 (7.31%)
No HR	10 (24.39%)	6 (14.63%)
**Cytogenetic response at 6 months after imatinib initiation**		
CCyR	3 (7.31%)	9 (21.95%)
PCyR/NMR	1 (2.43%)	3 (7.31%)
None	15 (36.58%)	10 (24.39%)
**Molecular response at 12 months after imatinib initiation**		
Complete	2 (4.87%)	8 (19.51%)
Major	5 (12.19%)	6 (14.63%)
No	12 (29.26%)	8 (19.51%)

CHR: Complete hematological response; PHR: Partial hematological response; CCyR: Complete cytogenetic response; PCyR: Partial cytogenetic response; NMR: No molecular response.

### Assessment of BCR-ABL gene expression (qRT-PCR analysis) in CML patients

**Figure 2:**
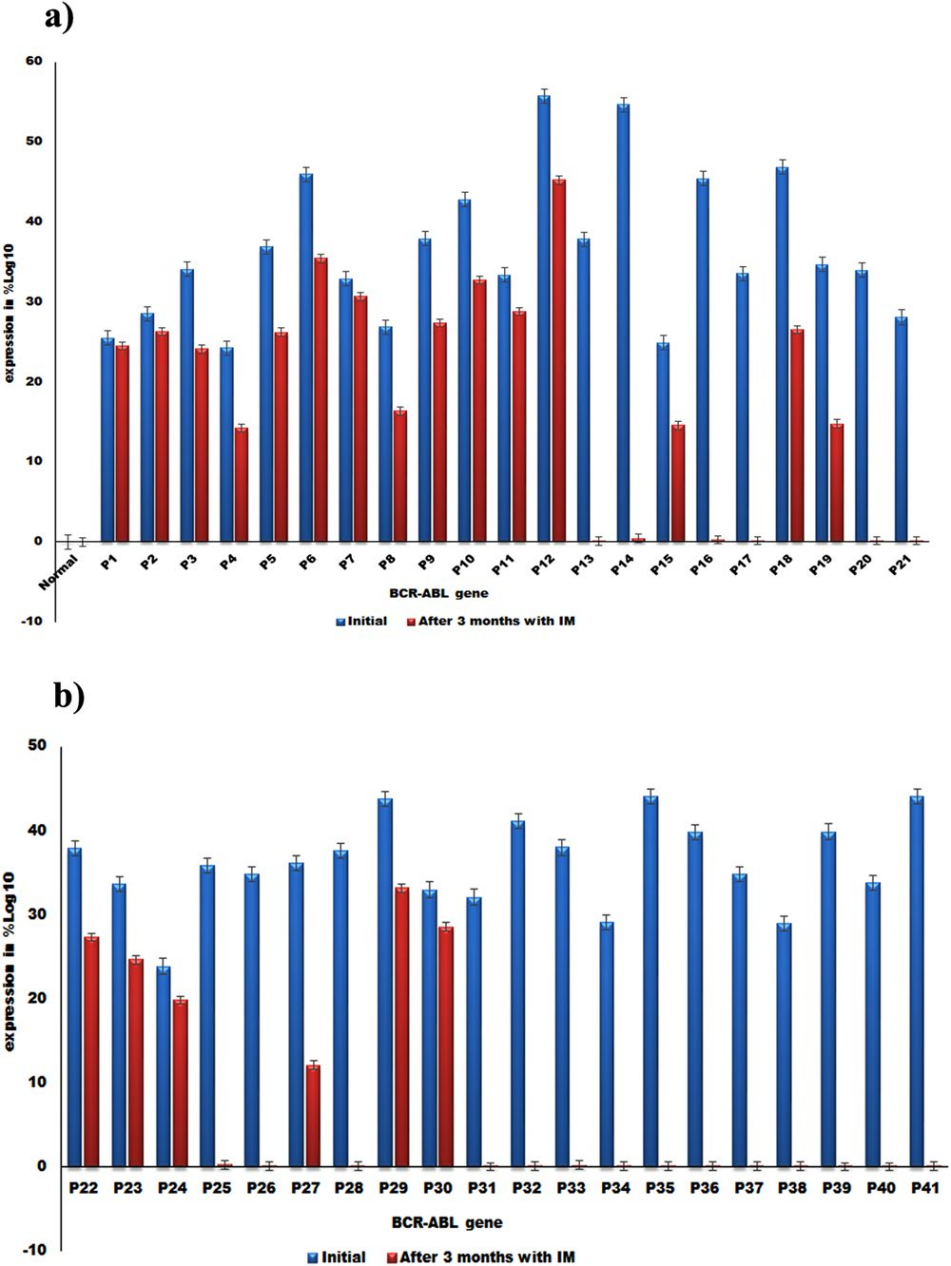
(**a**) Quantification of BCR-ABL gene expression in normal and CML patients (P1-P21) by qRT-PCR. (**b**) Quantification of BCR-ABL gene expression in CML patients (P22-P41) by qRT-PCR, 21/62 patients showed no fall in BCR-ABL gene expression even after 90 days treatment with imatinib (blue color), while (20/62) CML patients showed distinct fall in the BCR-ABL gene expression (red color).

The quantitative Real-time PCR (qRT-PCR) was performed in all (n = 41) CML patients before imatinib treatment and conspicuous BCR-ABL gene expression was observed and all these CML patients were put on imatinib treatment (Fig. 2a,b). After 90 days of treatment, the BCR-ABL gene expression was again assessed by using qRT-PCR in all the patients enrolled for the study. The results showed, 51.21% (21/41) CML patients had no appreciable change in the expression of BCR-ABL gene and 48.78% (20/41) patients showed significant fall in the expression of BCR-abl gene (Fig. 2a,b). With respect to cytogenetic response at 6 months after initiation of imatinib, complete cytogenetic response (CCyR) was observed in 29.26%, partial cytogenetic response (PCyR) in 9.75% of CML patients. With regards to molecular response at 12 months after initiation of imatinib, complete molecular response (CMoR) was observed in 24.39% and major molecular response (MMoR) in 26.82% of CML patients (Table 4).

At the end of 3 months of imatinib therapy, 29.26% (12 patients, 4 CP, 7 AP, 1 BC) of b2a2 transcript variant group registered CHR while 7.31% had PHR (Tables 3 and 4). In b3a2 transcript variant patient group, 19.51% (8 patients, all in CP) had CHR and 4.87% exhibited PHR (Tables 3 and 4). With respect to cytogenetic response at 12 months after imatinib initiation, 21.95% of b2a2 patients had CCyR, and 7.31% had PCyR. On the other hand, b3a2 transcript variants had CCyR in 7.31% and PCyR in 2.43% of patients (Tables 3 and 4). The molecular response at 12 months after imatinib treatment, 19.51% of b2a2 patients recorded CMoR while 14.63% had MMoR. On the contrary, only 4.87% of b3a2 transcript variant patients showed CMoR, 12.19% demonstrated MMoR while no molecular response was visible in 48.78% patients (Tables 3 and 4).

### Amplification and sequencing of the kinase domain (KD) of BCR-ABL gene

**Figure 3:**
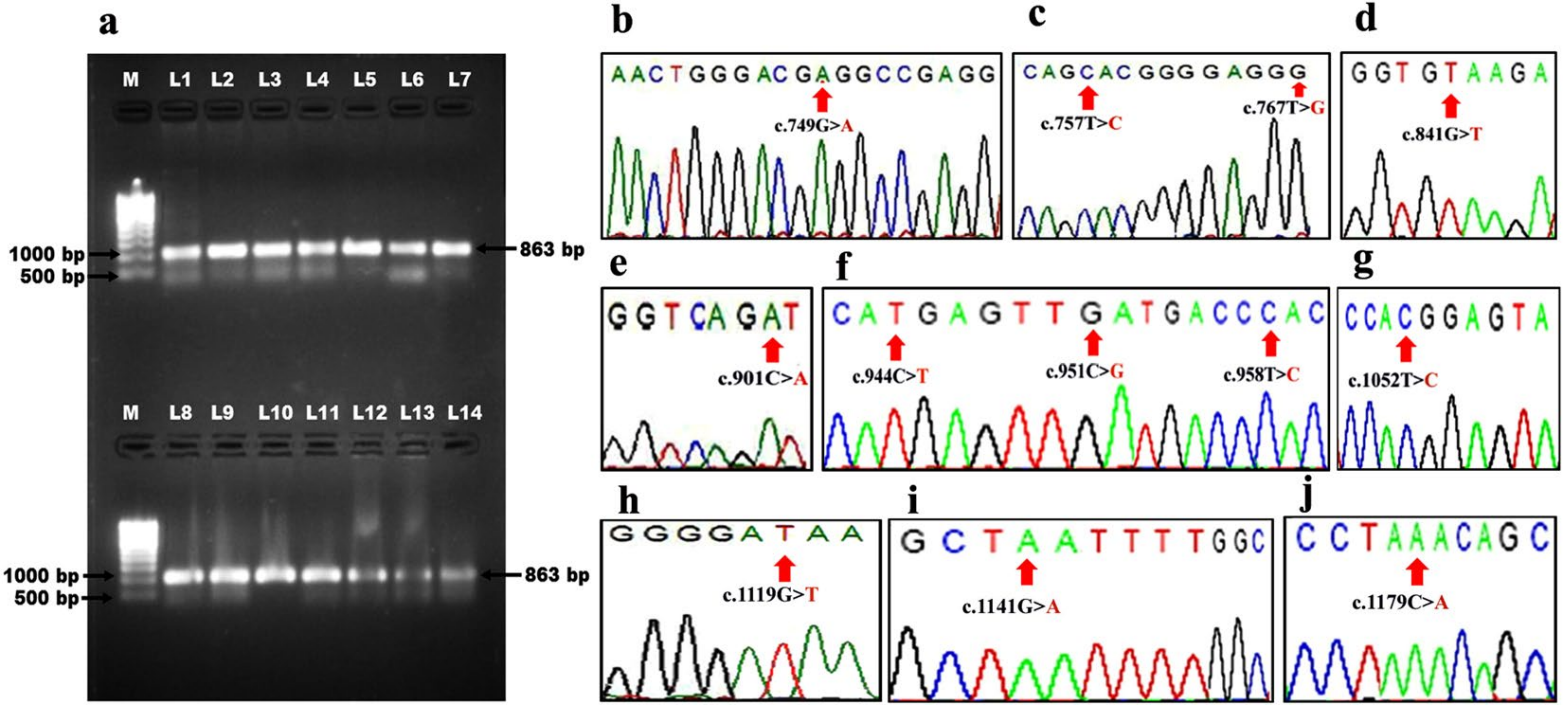
(**a**) PCR amplification of KD of BCR-ABL gene, Lane M: Super mix DNA ladder (Merck biosciences Pvt Ltd), Lane L1-L14, 863 bp PCR product of BCR-ABL kinase domain. The chromatogram showing the variations in the kinase domain of BCR-ABL gene depicting (**b**) c.749G > A mutation, (**c**) c.757T > C and c.767T > G mutations, (**d**) c.841G > T mutation, (**e**) c.901C > A mutation, (**f**) c.944C > T, c.951C > G and c.958T > C mutations, (**g**) c.1052T > C mutation, (**h**) c.1119G > T mutation, (**i**) c.1141G > A mutation and (**j**) c.1179 C > A mutation.

It is very well known that mutations in the kinase domain of BCR-ABL gene confer resistance to imatinib^12^. In all the 41 CML patients of our study, an 863 base pair fragment containing KD of BCR-ABL gene was PCR amplified in a semi-nested PCR (Fig. 3a) and sequenced. The sequence analysis showed mutations in 21/41 CML patients (Accession numbers MG641938 to MG641958). The sequence analysis of BCR-ABL gene in 13 out of these 21 patients (61.90%) unveiled 6 novel mutations, which were not reported so far in any database while, 8/21 (38.09%) patients indicated 6 known reported mutations (Table 5).

**Table 5:** Pathogenic mutations identified in BCR-ABL gene in CML patients.

Patient	Age	Sex	Disease Status	Mutation Base Change	Protein Consequence	GenBank ID	Reference
P1	40	M	CP	c.749G > A	p.(Gly250Glu)	MG641938	^3^
P2	66	M	CP	c.757T > C, c.767T > G	p.(Tyr253His), p.(Val256Gly)	MG641939	^3, 14^
P3	39	F	CP	c.767T > G	p.(Val256Gly)	MG641940	^14^
P4	68	F	CP	c.841G > T	p.(Glu281*)	MG641941	Novel
P5	39	F	CP	c.901C > A	p.(Leu301Ile)	MG641942	Novel
P6	56	M	CP	c.944C > T, c.951C > G, c.958T > C	p.(Thr315Ile), p.(Phe317Leu), p.(Tyr320His)	MG641943	Novel,^3,11^
P7	55	M	MBC	c.951C > G	p.(Phe317Leu)	MG641944	^11^
P8	52	F	CP	c.958T > C	p.(Tyr320His)	MG641945	Novel
P9	41	F	CP	c.1052 T	p.(Met351Thr)	MG641946	^3, 11^
P10	44	F	LBC	c.1119G > T	p.(Glu373Asp)	MG641947	Novel
P11	49	M	CP	c.1141 G > A	p.(Asp381Asn)	MG641948	Novel
P12	29	M	AP	c.1179C > A	p.(Tyr393*)	MG641949	Novel
P13	32	M	CP	—	—	—	—
P14	58	M	AP	—	—	—	—
P15	50	F	AP	c.901C > A	p.(Leu301Ile)	MG641950	Novel
P16	45	M	AP	—	—	—	—
P17	30	M	AP	—	—	—	—
P18	50	M	AP	c.841G > T	p.(Glu281*)	MG641951	Novel
P19	58	M	MBC	c.944C > T	p.(Thr315Ile)	MG641952	^3, 11^
P20	35	M	LBC	—	—	—	—
P21	44	F	CP	c.1179C > A	p.(Tyr393*)	MG641953	Novel
P22	45	F	CP	—	—	—	—
P23	41	F	MBC	c.944 C > T	p.(Thr315Ile)	MG641954	^3, 11^
P24	46	F	AP	c.841G > T	p.(Glu281*)	MG641955	Novel
P25	39	M	AP	—	—	—	—
P26	44	F	CP	—	—	—	—
P27	56	M	CP	c.1179C > A	p.(Tyr393*)	MG641956	Novel
P28	56	M	CP	—	—	—	—
P29	35	M	CP	c.901C > A	p.(Leu301Ile)	MG641957	Novel
P30	37	M	CP	c.944C > T	p.(Thr315Ile)	MG641958	^3, 11^
P31	55	M	CP	—	—	—	—
P32	49	F	CP	—	—	—	—
P33	62	F	CP	—	—	—	—
P34	58	F	CP	—	—	—	—
P35	57	M	AP	—	—	—	—
P36	61	M	AP	—	—	—	—
P37	57	M	CP	—	—	—	—
P38	47	F	CP	—	—	—	—
P39	59	F	CP	—	—	—	—
P40	63	M	AP	—	—	—	—
P41	48	M	CP	—	—	—	—

P: Patient, M: Male, F: Female, CP: Chronic Phase, AP: Accelerated Phase, MBC: Myeloid Blast Crisis, LBC: Lymphoid Blast Crisis.

Among the 21/41 TKD mutation positive cases, b3a2 fusion transcript type was observed in 71.42% (15/21) and b2a2 transcript variant in 28.57% (6/21) of patients. The TKD mutations indicating imatinib resistance were detected in 52.0% (13/25) of all patients in CP, 27.27% (3/11) of all patients in AP and 80.0% (4/5) of all patients in BC. The occurrence of mutations was higher in the advanced phase of the disease (BC) than in the CP, indicating an increase in genomic instability as the disease progresses. Patients with the initial high Sokal scores had the higher incidence of mutations when compared to patients with the low or intermediate Sokal scores (Table 4).

In detail, the sequence analysis of BCR-ABL gene in patient P1 (CP) showed one reported c.749G > A mutation resulting in p.(Gly250Glu)^3^ amino acid variation (GenBank accession number: MG641938) (Fig. 3b), patient P2 (CP) had two reported mutationc.757T > C, c.767T > G resulting in p.(Tyr253His)^3^ and p.(Val256Gly)^14^ amino acid variation respectively (GenBank accession number: MG641939) (Fig. 3c) and patient P3 (CP) revealed one novel mutation c.767T > G resulting in p.(Val256Gly) amino acid variation (GenBank accession number: MG641940). Three patients P4 (CP), P18 (AP) and P24 (AP) expressed novel c.841G > T mutation (3/21, 14.2%) corresponding to Glu281* stop codon resulting in the truncated protein formation (GenBank accession numbers: MG641941, MG641951, and MG641955) (Fig. 3d). Similarly, patients P5 (CP), P15 (AP) and P29 (CP) demonstrated novel c.901C > A missense mutation (3/21, 14.2%) leading to the replacement of Leu with Ile at 301 position p.(Leu301Ile) in KD of BCR-ABL gene (GenBank accession numbers: MG641942, MG641950, and MG641957) (Fig. 3e and Table 5).

Patient P6 (CP) presented with 2 reported mutations c.944C > T, c.951C > G and 1 novel c.958 T > C mutation resulting in p.(Thr315Ile)^3,11^, p.(Phe317Leu)^3,11^ and p.(Tyr320His) amino acid variations respectively (GenBank accession number: MG641943) (Fig. 3f and Table 5).

Patients P19 (MBC), P23 (MBC) and P30 (CP) showed widely reported mutation c.944C > T resulting in p.(Thr315Ile)^3,11^ amino acid variation (GenBank accession numbers: MG641952, MG641954, and MG641958). Patient P7 (MBC) revealed one reported mutation c.951C > G resulting in p.(Phe317Leu) amino acid variation (GenBank accession number: MG641944)11 (Fig. 3f and Table 5).

The P8 (CP) patient exhibited novel mutation c.958T > C (1/21, 4.7%) corresponding to p.(Tyr320His) amino acid variation (GenBank accession number: MG641945), patient P9 (CP) showed one reported c.1052T > C mutation resulting in p.(Met351Thr)^3,11^ amino acid variation (GenBank accession number: MG641946) (Fig. 3g) while, P10 (LBC) patient brought out a novel mutation c.1119G > T (1/21, 4.7%) corresponding to p.(Glu373Asp) amino acid substitution (GenBank accession number: MG641947) (Fig. 3h). Patient P11 (CP) unwrapped a novel mutation c.1141G > A (1/21, 4.7%) resulting in p.(Asp381Asn) amino acid variation (GenBank accession number: MG641948) (Fig. 3i). It was visible in patients P12 (AP), P21 (CP) and P27 (CP) a novel c.1179C > A variation (14.2%) leading to the p.(Tyr393*) stop codon resulting in truncated protein formation (GenBank accession numbers: MG641949, MG641953, and MG641956) (Fig. 3j and Table 5).

### Molecular dynamic (MD) simulation of wild-type and mutated ABL structures

**Figure 4:**
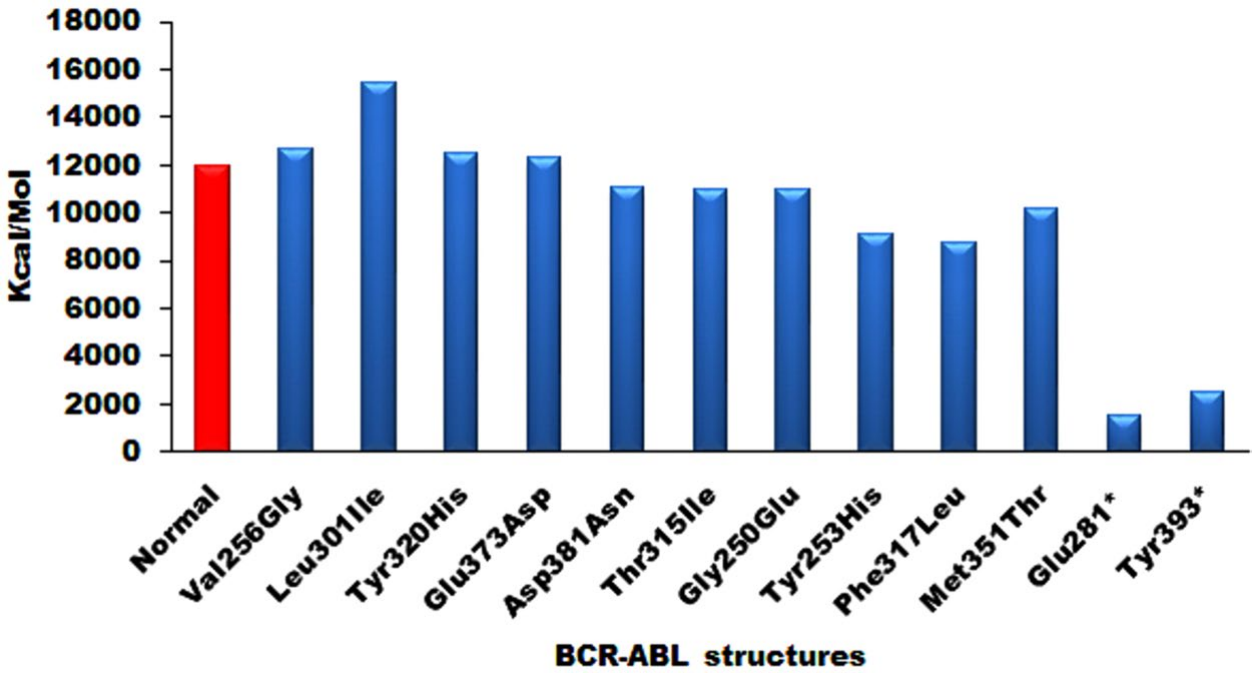
The energy variations of normal and mutated ABL structures generated after 10 ns of MD simulations.

The normal and mutant ABL structures were subjected to MD simulations to know the energy variations. MD Simulations up to 10 ns revealed 20,000 conformations for each ABL structure and the structures showing lowest conformational energy values were taken to correlate with normal energies (Fig. 4). The normal  ABL structure was stabilized at around the 12000 kcal/mol. The mutated structures formed with Val256Gly, Leu301Ile, Tyr320His, and Glu373Asp showed increased energy values compared with normal ABL structure, whereas mutated structures Asp381Asn, Thr315Ile, Gly250Glu, Tyr253His, Phe317Leu, and Met351Thr showed reduced energy values. Interestingly, Glu281* and Tyr393* frame shift mutated structures showed very low energy compared with normal ABL energy values. All these results were suggestive of the presence of non-functional protein in these patients.

### *PDBSum* analysis of wild-type and mutated ABL structures

The *PDBSum* conformational fluctuations in all the mutated ABL structures were measured and compared with the normal ABL structure. The normal ABL structure had 7 sheets, 1 β-α-β unit, 11 beta hairpins, 9 β-bulges, 23 strands, 19 helices, 16 helix-helix interactions, 48 β-turns, and 5 γ-turns (Table 6 and Supplementary Fig. S1). The number of sheets, beta hairpins, β-bulges, and strands was reduced in p.(Glu281*) and p.(Tyr393*) mutated structures while decreased number of helices and β-turns were observed in p.(Glu281*), p.(Tyr393*), p.(Asp381Asn), p.(Glu373Asp), p.(Phe317Leu), p.(Leu301Ile), p.(Met351Thr), p.(Thr315Ile), p.(Val256Gly), p.(Tyr253His) and p.(Tyr320His) mutated protein structures (Table 6 and Supplementary Figs S2–S12). The p.(Glu281*) mutated structure had no helix-helix interactions and p.(Tyr393*) mutated structure had only 2 helix-helix interactions compared with normal ABL structure. The p.(Tyr393*), p.(Asp381Asn), p.(Glu373Asp), p.(Leu301Ile), p.(Met351Thr) and p.(Tyr320His) mutated structures showed an increased number of γ-turns, whereas, p.(Phe317Leu) and p.(Thr315Ile) mutant structures showed a reduced number of γ-turns (Table 6 and Supplementary Figs S2–S12).

**Table 6:** The *PDBSum* analysis of wild-type ABL structure with mutated ABL structures.

BCR-ABL Structure	Sheets	Beta alpha beta units	Beta heparins	Beta bulges	Strands	Helices	Helix-Helix interactions	Beta turns	Gamma turns
Wild-type	7	1	11	9	23	19	16	48	5
p.(Glu281*)	4	1	8	7	15	5	0	29	5
p.(Tyr393*)	6	1	10	9	21	10	2	38	6
p.(Asp381Asn)	7	1	11	9	23	18	16	46	6
p.(Glu373Asp)	7	1	11	9	23	18	16	46	7
p.(Phe317Leu)	7	1	11	9	23	18	16	47	4
p.(Gly250Glu)	7	1	11	9	23	19	16	48	5
p.(Leu301Ile)	7	1	11	9	23	18	16	46	6
p.(Met351Thr)	7	1	11	9	23	18	16	47	6
p.(Thr315Ile)	7	1	11	9	23	18	16	47	4
p.(Val256Gly)	7	1	11	9	23	17	16	49	5
p.(Tyr253His)	7	1	11	9	23	19	16	47	5
p.(Tyr320His)	7	1	11	9	23	18	16	46	8

### Structural superimposition

All the mutated ABL structures when superimposed with the wild-type ABL structure, highly variable root-mean-square deviation (RMSD) values were observed, ranging from a minimum of 0.867 Å for p.Gly250Glu mutant to a maximum of 5.036 Å for p.(Glu281*) mutant (Supplementary Table 1).

### Molecular docking with imatinib, nilotinib, bosutinib, and bafetinib

**Figure 5:**
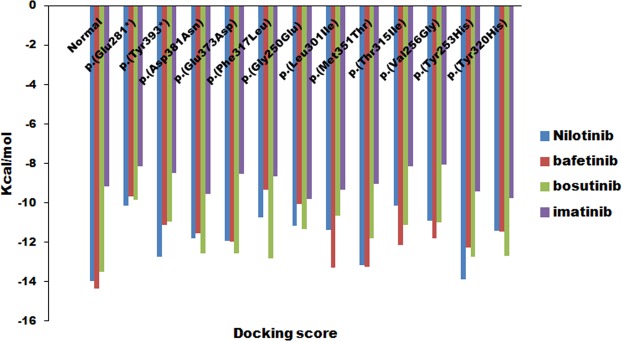
Molecular docking of imatinib, nilotinib, bosutinib, and bafetinib into the active site cavity of normal and mutated ABL structures. The docking score indicates the binding affinity of imatinib, nilotinib, bosutinib, and bafetinib into the active site. The lowest docking the score indicates the highest binding affinity to the enzyme.

**Figure 6:**
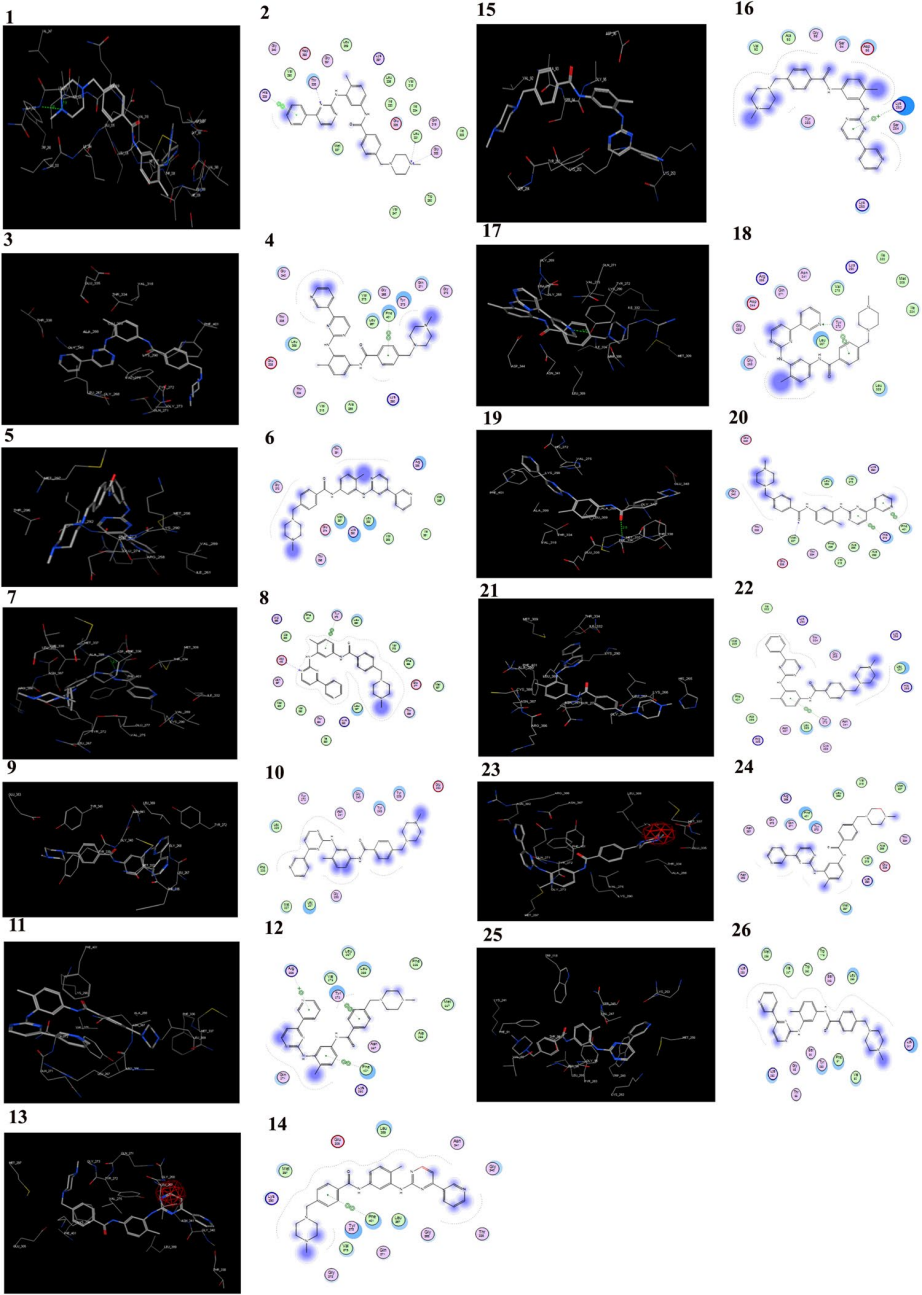
Molecular docking of imatinib into the active site of wild-type (1, 2) and 3,4 p.(Asp381Asn), 5,6 p.(Glu281), 7,8 p.(Glu373Asp), 9,10 p.(Gly250Glu), 11,12 p.(Leu301Ile), 13,14 p.(Met351Thr), 15,16 p.(Phe317Leu), 17,18 p.(Thr315Ile), 19,20 p.(Tyr253His), 21, 22 p.(Tyr320His), 23, 24 p.(Tyr393) and 25, 26 p.(Val256Gly) mutant ABL structures respectively.

The molecular docking analysis of wild-type ABL structure with nilotinib showed the docking score of-13.9508 kcal/mol and was found to be interacting with Asp392 and Thr338 forming a total of two hydrogen bonds (Supplementary Fig. S13). The bafetinib showed docking score of −14.3507 kcal/mol and was seen to interact with Glu335 by forming one hydrogen bond (Supplementary Fig. S14). The bosutinib docking score was −13.5014 kcal/mol and was interacting with His339 by forming one hydrogen bond (Supplementary Fig. S15) and the imatinib showed docking score of −9.1593 kcal/mol by interacting with Leu321, Gly322 by forming two hydrogen bonds (Table 7, Fig. 5 and Supplementary Fig. S16). All the mutated structures including p.(Glu281*) and p.(Tyr393*) mutated structures when docked individually, showed the highest docking score with imatinib with distorted binding due to the disruption of van der Waals interactions and hydrogen bonds (Fig. 6). The other mutated ABL structures when docking with nilotinib, bosutinib and bafetinib drugs showed the lowest docking score compared with normal (Table 7 and Supplementary Figs S17–S64).

**Table 7:** Molecular docking interaction of nilotinib, bafetinib, bosutinib and imatinib with the active site residues in wild-type and mutated ABL structures.

BCR-ABL Structure	Drug	Docking Score	No. of H-bonds	Interacting residues	H-bond length (Å)
Wild-type	Nilotinib	−13.9508	2	Asp392	2.6
				Thr338	1.5
	Bafetinib	−14.3507	1	Glu335	1.7
	Bosutinib	−13.5014	1	His339	2.7
	Imatinib	−9.1593	2	Leu321	1.9
				Gly322	2.6
p.(Glu281*)	Nilotinib	−10.1331	1	Glu274	3.1
	Bafetinib	−9.6812	1	Lys266	2.6
	Bosutinib	−9.8423	3	Arg258	2.8
				Thr291	2.8
				Thr291	3.3
	Imatinib	−8.1682			
p.(Tyr393*)	Nilotinib	−12.7442			
	Bafetinib	−11.1293	1	Lys290	2.9
	Bosutinib	−10.937	1	Try272	2.1
	Imatinib	−8.4852			
p.(Asp381Asn)	Nilotinib	−11.7852			
	Bafetinib	−11.559			
	Bosutinib	−12.5556	1	Met337	2.9
	Imatinib	−9.5735			
p.(Glu373Asp)	Nilotinib	−11.9278	1	Lys290	1.7
	Bafetinib	−11.9758	1	Gln271	2.3
	Bosutinib	−12.572	1	Tyr334	2.7
	Imatinib	−8.5554	1	Asp400	2.7
p.(Phe317Leu)	Nilotinib	−10.7352			
	Bafetinib	−9.335	3	Thr98	2.8
				Val247	2.9
				Tyr283	2.3
	Bosutinib	−12.8439	2	Trp280	3.4
				Tyr283	3.2
	Imatinib	−8.6684			
p.(Gly250Glu)	Nilotinib	−11.1557			
	Bafetinib	−10.0738	1	Lys290	2.7
	Bosutinib	−11.3189	2	Thr334	3.2
				Met337	2.5
	Imatinib	−9.8071			
p.(Leu301Ile)	Nilotinib	−11.3638			
	Bafetinib	−13.2823			
	Bosutinib	−10.6507	1	Arg386	3.2
	Imatinib	−9.3564			
p.(Met351Thr)	Nilotinib	−13.1617	1	Tyr272	2.8
	Bafetinib	−13.2658	1	Lys290	3.1
	Bosutinib	−11.8079	1	Tyr234	2.5
	Imatinib	−9.0595			
p.(Thr315Ile)	Nilotinib	−10.1496			
	Bafetinib	−12.1359	1	Tyr272	3.2
	Bosutinib	−11.1358	1	Lys290	2.5
	Imatinib	−8.1452	1	Tyr272	1.7
p.(Val256Gly)	Nilotinib	−10.9289	1	Ser94	1.9
	Bafetinib	−11.8051			
	Bosutinib	−8.0136	2	Ser94	2.6
				Tyr283	2.4
	Imatinib	−10.0668			
p.(Tyr253His)	Nilotinib	−13.8685	1	Glu274	2.6
	Bafetinib	−12.2542			
	Bosutinib	−12.7471	1	Tyr334	2.9
	Imatinib	−9.4287	1	Met337	2.5
p.(Tyr320His)	Nilotinib	−11.4102			
	Bafetinib	−11.4813			
	Bosutinib	−12.7085	3	Met337	2.4
				Asn341	2.1
				Asp400	3.3
	Imatinib	−9.7669

The lowest docking score indicated the elevated strength and affinity of the ligand with the binding site. The enlarged docking score with the binding site explained the reduced affinity for and poor binding with its ligands in the mutated ABL structures. The highest docking score was observed in all the mutated structures when docked with imatinib explaining very low affinity towards the enzyme (Table 7 and Supplementary Figs S17–S64). Overall, the imatinib was found to be showing high variations in the binding orientation with all mutant structures leading to the disruptive interactions between imatinib and the kinase domain (Fig. 6). All these results overwhelmingly explained that the imatinib resistance in these CML patients was due to the mutations in the ABL kinase domain.

### Polymorphism Phenotyping (PolyPhen) and Sift score calculation

**Figure 7:**
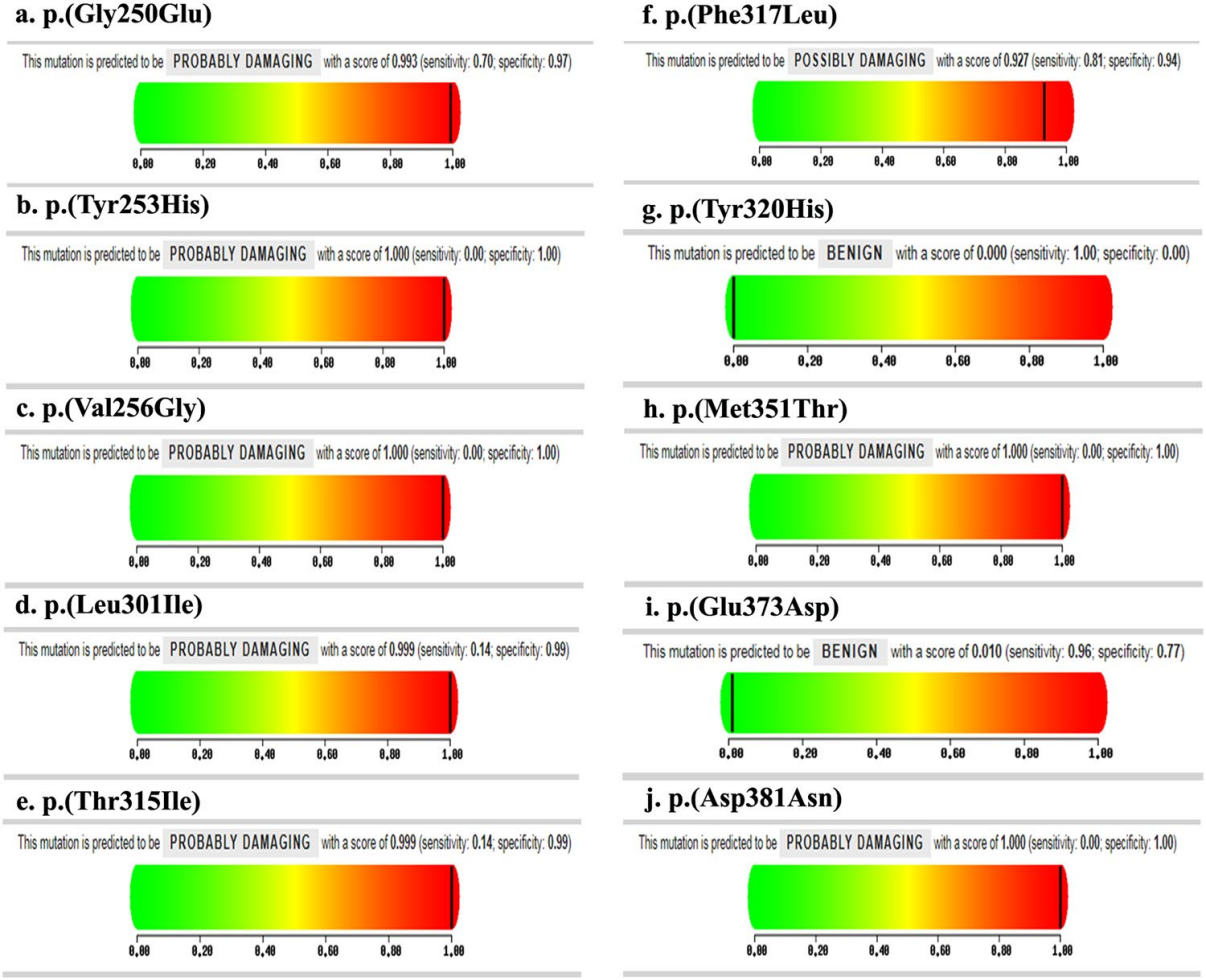
The PolyPhen-2 analysis, the mutant BCR-ABL protein sequences when submitted to the PolyPhen-2 online server (**a**–**j**). Showed PolyPhen score 1.00 in most of the mutations which were probably damaging the ABL structure.

Overall, 21 CML patients showed 12 BCR-ABL gene mutations (6 novel and 6 reported) and these mutated BCR-ABL protein sequences were submitted independently to the SIFT program to check their tolerance. Among the 12 mutant BCR-ABL sequences, 8 mutants were found to be deleterious, having the tolerance index score of <0.05 and the results were given in Table 8. It observed that 8 mutants, the mutants Y253H, V256G, M351T, and D381N were showed to be deleterious with a tolerance index score of 0.00. The mutants L301I, T315I, G250E and F317L showed a tolerance index score of 0.01, 0.01, 0.03 and 0.04 respectively (Table 8 and Fig. 7) indicating a probable pathogenic in nature.

**Table 8:** List of mutants that were predicted to be functional significance by SIFT score and PolyPhen in BCR-ABL gene.

Patient	Mutation Base Change	Amino acid change	Sift score (Tolerance index)	PolyPhen score	Sensitivity	Specificity
P1	c.749G > A	G250E	0.03	0.993	0.7	0.97
P2	c.757T > C	Y253H	0	1	0	1
P3	c.767T > G	V256G	0	1	0	1
P4	c.841G > T	E281*	—	—	—	—
P5	c.901C > A	L301I	0.01	0.999	0.14	0.99
P6	c.944C > T	T315I	0.01	0.999	0.14	0.99
P7	c.951C > G	F317L	0.04	0.927	0.81	0.94
P8	c.958T > C	Y320H	0.24	0	1	0
P9	c.1052T	M351T	0	1	0	1
P10	c.1119G > T	E373D	0.18	0.01	0.96	0.77
P11	c.1141G > A	D381N	0	1	0	1
P12	c.1179C > A	Y393*	—	—	—	—
P15	c.901C > A	L301I	0.01	0.999	0.14	0.99
P18	c.841G > T	E281*	—	—	—	—
P19	c.944C > T	T315I	0.01	0.999	0.14	0.99
P21	c.1179C > A	Y393*	—	—	—	—
P23	c.944C > T	T315I	0.01	0.999	0.14	0.99
P24	c.841G > T	E281*	—	—	—	—
P27	c.1179C > A	Y393*	—	—	—	—
P29	c.901C > A	L301I	0.01	0.999	0.14	0.99
P30	c.944C > T	T315I	0.01	0.999	0.14	0.99

The structural levels of variation were determined by PolyPhen-2 online program server. All the 12 mutant BCR-ABL protein sequences were also submitted to the PolyPhen online server. As shown in Table 8, the mutants Y253H, V256G, M351T, and D381N were found to be causing serious damage to ABL structure with PolyPhen score 1.00, sensitivity 0.00 and specificity 1.00. The mutants L301I, T315I, G250E, and F317L showed PolyPhen score 0.999, 0.999, 0.993 and 0.927 with sensitivity 0.14, 0.14, 0.71, 0.81 and specificity was 0.99, 0.99, 0.97 and 0.94 respectively indicating a probably damaging the ABL structure (Table 8 and Fig. 7).

Among the imatinib resistant patients, only 4 patients with BCR-ABL mutation could be kept on nilotinib under patient assistance programmes and they responded well in terms of CHR. Cytogenetic and molecular responses are to be checked at the end of 12 months.

## Discussion

The episode and evolution of CML have been connected to the presence of the Philadelphia chromosome due to a reciprocal translocation between chromosomes 9 and 22 resulting into BCR-ABL gene fusion^7^. The resistance to drugs or problems in the management of CML is a function of the property and ability of the BCR-ABL oncogene to undergo mutations, develop mutant forms and BCR-ABL phenotypes^1–8,12^.

In this study, all the CML patients were treated with imatinib for 3 months and at the end of the treatment, 33.87% of patients did not achieve CHR, while 8.06% patients showed PHR. Rest of them, 58.06% patients, showed CHR and responded well to imatinib (Table 1). Out of 21 imatinib resistant patients, 61.90% (13 patients) were in CP of which 8 patients were with high Sokal scores and 5 with intermediate Sokal score (Table 2). Patients in AP and BC were 19.04% (4/21) each, had high Sokal scores (Table 2). The response rate to imatinib in our study was lower than earlier worldwide reports. The exact cause was not clear. The other reasons for this apparent discrepancy could possibly be a) partial treatment with Hydroxyurea in some patients before the confirmation of diagnosis could have interfered with the staging of the phases of the disease and with the Sokal scoring system. b) Patient compliance was not up to the mark, particularly in the less educated. The perception in some was that a pill was too inadequate for a cancerous condition. c) It is possible that high initial Sokal scores in our CML-CP patients could possibly be one of the reasons.

In our study, 41/62 CML patients participated in the cytogenetic and molecular studies and remaining 21 patients declined participation. All these 41 CML patients were further subjected to qualitative and quantitative BCR-ABL expression and gene mutations analyses. It is very well known that the progression of CML in the patients is due to the distinct expression of BCR-ABL gene^8^ and drugs like imatinib show decrease in this expression and helps reverse the disease to a greater extent. However, the mutations in the kinase domain of BCR-ABL gene contributed to the imatinib resistance in CML patients^12,15,16^.

In our study, the frequencies of b3a2 and b2a2 fusion transcript variants were found to be 60.97% and 39.02% respectively which corresponded with the studies done in this part of our country (b3a2 56.25%, b2a2 41.25%)^17^. Kagita *et al*., 2018 in their study observed 63.53% of CML patients were having b3a2 and 36.36% b2a2 transcript variants and another Indian study noted the frequencies of b3a2 and b2a2 to be 66.82% and 28.84% respectively^18^. Similarly, a CML study from Lahore, Pakistan showed a frequency of 63.33% for b3a2 and 36.66% for b2a2^19^.

In the present study, males had a higher frequency of b3a2 transcript variant (36.58%) compared to females (24.39%) as was also observed in the earlier reports from India^20^, while b2a2 transcript variant patients did not show male predominance (21.95% males and 17.07% females) as could be noted from the Sudanese study^21^.

The CHR, CCyR and CMoR frequencies in our study were found to be higher in b2a2 (29.26%, 21.95%, and 19.51% respectively) transcript variants compared to b3a2 group (19.51%, 7.31% and 4.87%) indicating the possible difficulty in treating the latter group with imatinib (Table 4). Our results were similar to the earlier reports from the Indian studies on CML^20,22^. Latagliata *et al*., 2011 reported in Italian CML patients the CHR, CCyR and CMoR frequency of 27.8%, 49.1% and 15.57% respectively^23^, which corresponded well with our results.

To know whether mutations in this gene really contributed in the disease progression, the kinase domain region of BCR-ABL gene was PCR amplified and the PCR products were sequenced in all the 41 CML patients who were under imatinib treatment. In our study group, mutations were detected in 21/41 imatinib-treated patients who showed continuous expression of BCR-ABL gene, while no mutations and correspondingly distinct fall in the of BCR-ABL gene expression were noted in patients who achieved CHR. The incidence of mutation frequency was found to be more in b3a2 fusion transcript type (71.42%) compared to b2a2 transcript variant (28.57%). Similar results were observed in other Indian studies on CML patient population^20^. Among 21 patients with mutations, 61.9% (13/21) showed 6 novel mutations of which two novel mutations resulted in the truncation at amino acids Glu281* and Tyr393*. Four novel missense mutations Leu301Ile, Tyr320His, Glu373Asp, and Asp381Asn were found which were not previously reported in any database, thus explaining the uniqueness of these mutations in this ethnic population. Previously reported mutations Val256Gly, p.Thr315Ile, p.Gly250Glu, p.Tyr253His, p.Phe317Leu and p.Met351Thr were observed in 8 CML resistant patients^4,11,12,14^ (Fig. 3).

**Figure 8:**
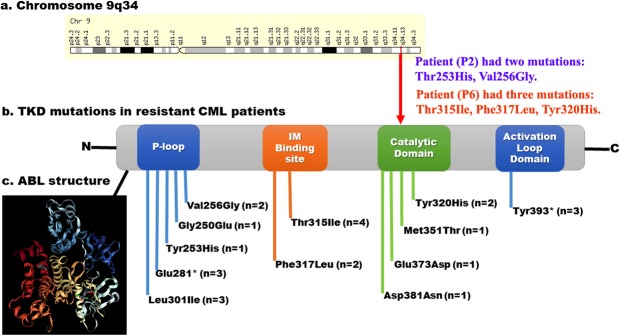
Distribution of mutations detected in BCR-ABL gene in resistant CML patients. (**a**) The ABL gene located on the long arm of the Chromosome 9q34. (**b**) Distribution of TKD mutations in functional domains. (**c**) Secondary structure of ABL tyrosine kinase.

Mutations in the phosphate (P-loop) region of ABL gene, accounted for up to 48% of all mutations in imatinib resistant cases^4^. Interestingly, in our study also, 47.61% (10/21) resistant patients had P-loop mutations such as Val256Gly, Gly250Glu, Tyr253His, Glu281* and Leu301Ile with Sift score of 0.00, PolyPhen score 1.00 (Table 8 and Fig. 7) and causing destabilization of the ABL structural conformation (required for imatinib binding), were noted. Because of these mutations, an increased transforming potential and loss of sensitivity to imatinib were caused leading to worse prognosis^4^ (Fig. 8).

The Gatekeeper mutations (Thr315Ile and Phe317Leu) is one of the most frequent mutations arising in patients on imatinib therapy, occurring between 4–19% of resistant CML cases^12^. In the present study, 33.33% (7/21) resistant patients showed Gatekeeper mutations Thr315Ile and Phe317Leu with a tolerance index score of 0.01 and PolyPhen score 0.999 (Table 8), indicating a probable cause for the damage of the ABL structure. The residue Thr315 forms a fundamental hydrogen bond with imatinib and gets disrupted by the replacement with isoleucine. This prevents the imatinib localization within the ATP binding pocket by consequent stearic hindrance thereby causing resistant to all ABL kinase inhibitors^12^ (Fig. 8).

A series of mutations are reported in the catalytic domain ABL gene which can also affect imatinib binding. In our study, 23.80% (5/21) resistant CML patients had highly deleterious catalytic domain mutations, Tyr320His, Met351Thr, Glu373Asp and Asp381Asn with a tolerance index score of 0.00 and PolyPhen score 1.00 (Table 8). The activation loop domain of the ABL kinase is the major regulatory component which can adopt an open and active or closed and inactive conformation. Inactive and closed configuration is required for imatinib activity. But, the mutations in the activation loop to activate the open and active configuration, thus causing the loss imatinib activity^12^. In this study, 14.28% (3/21) resistant patients had activation loop domain mutation, Tyr393*, activating the open and active configuration and the causing imatinib resistance^4,11,12^ (Fig. 8).

It is known that E255V interacts with imatinib through van der Waals interactions and also forms one hydrogen bond with a bond length of 1.9 Å. At this site, normally, the enzyme also interacts with ATP^4,11^. The replacement of Glu255 with Val causes the formation of inactive BCR-ABL protein with reduced sensitivity to imatinib while retaining the kinase activity^4,11,12^. Interestingly, the molecular docking analysis in our study, revealed incorporation of amino acids Gly250, Tyr253, Glu281 and Leu301 (23.8%) at the P-loop of the kinase domain of the BCR-ABL and the mutations of p.Gly250Glu (1/21 4.7%), p.Tyr253His (1/21 4.7%) (Fig. 3). One recent study, showed substitution of Tyr253 with Phe or His resulted in the increased damaging score^4,11^. Furthermore, the Tyr253Phe mutation, when engineered into BCR-ABL has demonstrated oncogenic activity in rat models^11,24,25^. These results suggested that theTyr253His or Phe played a potential role in increasing the genetic instability and driving the disease to a more advanced phase^11,24^. Interestingly, 4.7% of our study group showed these mutations. The high instability of BCR-ABL protein was observed in Glu281* (3/21 14.2%) and Leu301Ile (3/21 14.2%) mutations in the present study group. The residue Glu281 is highly stable and has an important role in the maintenance and functioning of ABL1 gene^26^. In a study, Barnes *et al*., 2005 identified the Glu281Lys mutation in BCR-ABL cell lines and showed sensitivity to imatinib, ponatinib^26^. In our study, 3 patients (1 CP, 2 AP) showed mutation c.841G > T resulting in Glu281* truncated protein formation. All these three patients exhibited resistance to imatinib treatment due to incomplete protein formation.

Recent studies demonstrated Thr315Ile and Phe317Leu occupied (5/37, 14%) mutations in the gatekeeper region of BCR-ABL gene in CML patients^11^. Our study, also showed mutations p.Thr315Ile (4/21 19%) and p.Phe317Leu (2/21 9.52%) in CML patients. The catalytic domain mutations p.Tyr320His, p.Met351Thr, p.Glu373Asp and p.Asp381Asn (5/21 23.80%) and the activation loop domain mutations Tyr393* (3/21 14.28%) in our study caused conformational changes in the BCR-ABL protein, exhibiting highest docking with imatinib (Fig. 6) compared to nilotinib, bosutinib and bafetinib (Table 7 and Supplementary Figs S17–S64) thus leading to poor imatinib binding.

The comparative structural analysis between normal and mutant ABL structures indicated distinct variations distributed in both domain and non-domain regions as indicated by the RMSD values (Supplementary Table 1). The molecular dynamic simulation studies exhibited variable energy fluctuations and conformational changes in mutant ABL structures clearly explaining that the mutations were responsible for the energy variations in the ABL structures (Fig. 4). The *PDBSum* analysis also revealed variable structural conformations such as decreased sheets, β-α-β units, hairpins, β-bulges, strands, helices, helix-helix interactions, β-turns, and γ-turns in the mutant structures compared with normal (Table 6 and Supplementary Figs S1–S12). Therefore, if resistance to imatinib is found on BCR-ABL gene analysis, as in our study, 2^nd^ or 3^rd^ generation drugs might be needed.

In conclusion, in the present study, intended for identifying mutations in the BCR-ABL gene in imatinib resistant patients, several novel mutations in the KD region of BCR-ABL causing non-functional protein, were identified. These mutations were distributed in the P-loop, Gatekeeper, catalytic and activation loop domains of the enzyme and resulted in poor imatinib binding to the ATP region of BCR-ABL protein causing imatinib resistance in these patients. The results highlight the need for the BCR-ABL gene sequence analysis in the CML patients to understand any variations in the gene for designing therapeutic modalities such as dose elevation or considering second, third and fourth generation drugs, as early as required, for good outcomes and long-term prognosis.

## Materials and Methods

### Patients and Sample Collection

Sixty-two (n = 62) CML patients were treated in the Hematology Department of Sri Venkateswara Institute of Medical Sciences (SVIMS), Tirupati, Andhra Pradesh, India between April 2010 to March 2018. After the confirmation of the diagnosis of CML, 35 patients were in chronic phase (CP), 21 were in accelerated phase (AP) and 6 were in blast crisis (BC). The Sokal score was calculated following the standard method for each patient before starting imatinib therapy^17,20,22^.

The imatinib treatment administration for CP patients was 400 mg/day and for AP/BC was 600 mg/day. The dosage of imatinib was escalated appropriately (600 mg/day to 800 mg/day) in the patients who did not fulfil the response criteria. Characterization of resistance was based on the recommendations by European LeukemiaNet 2009 guidelines^13^. The patients not achieving complete haematological response (CHR) by 3 months, no cytogenetic response by 6 months or Major Molecular Response (MMR) by 12 months were categorised as primary resistant and patients losing the previously achieved hematologic or cytogenetic response or patients exhibiting a consecutive 1 log increase in transcript level after achieving an MMR were categorized as secondary resistant patients^11,27^.

The quantitative BCR-ABL gene expression and kinase domain of BCR-ABL gene mutation analysis was done in 41/62 CML patients and 21 patients declined to participate in the study. 5 mL of peripheral blood was collected from all the CML patients [mean age ± SD = 53.73 ± 6.3] and 30 apparently healthy controls [mean age ± SD = 48.37 ± 8.5] who were Ph-negative. The patients’ clinical features were described in Table 1.

Written informed consents were obtained from all the study subjects. The study has been approved by the Institutional Ethics Committee (IEC No. 709) at Sri Venkateswara Institute of Medical Sciences and University, Tirupati-517507, Andhra Pradesh, India. All the methods in the study were performed in accordance with relevant guidelines and regulations.

### Cytogenetic analysis

The Buffy coat containing peripheral blood mononuclear cells (PBMCs) were separated from the peripheral blood by using Ficol-gradient centrifugation method (GE Healthcare) and the cells were washed with the 1X PBS (phosphate buffer saline). The buffy coat containing PBMCs were cultured in RPMI 1640 tissue culture medium (Himedia, Mumbai, India) along with 10% fetal bovine serum (FBS) (Himedia, Mumbai, India) for 24 h^18^. The chromosomal preparations were obtained using the standard procedure and were subjected to GTG-banding. At least 20 to 30 metaphases were analyzed and karyotyped according to the International System for Cytogenetic Nomenclature (ISCN) 2009^27^.

### Fluorescence ***in situ*** hybridization (FISH) analysis

The FISH analysis was carried out using dual color/dual fusion locus BCR-ABL specific fluorescent probes on cells obtained from unstimulated cultures (Vysis, AbottMolecular Inc., Des Plaines, IL) for detection of BCR-ABL fusion gene (t(9;22)(q34;q11) translocation) according to the manufacturer’s instructions. The analysis was done on an Olympus BX61 fluorescent microscope with appropriate filters using the Applied Spectral Imaging Software^28^.

### RNA isolation, cDNA synthesis

Total RNA was extracted from the CML patient’s peripheral blood using MEDOX-Easy Spin Column Total RNA Minipreps Super Kit (Medox Biotech India Pvt. Ltd, MX-0617-03). The total RNA was reverse transcribed to cDNA using High-Capacity cDNA Reverse Transcription Kit (Applied Biosystems, PN4375222)^29^.

### BCR-ABL (b3a2 and b2a2) fusion transcript analysis

The qualitative RT-PCR was used to amplify the b3a2 and b2a2 transcripts from the above CML patients’ cDNA by using the primers of standardized PCR protocol of BIOMED1^30^. The sequences of the primers were given below:

BCR-b1-A 3086 (22): GAAGTGTTTCAGAAGCTTCTCC

ABL-a3-B 458 (21): GTTTGGGCTTCACACCATTCC

BCR-b2-C 3126 (21): CAGATGCTGACCAACTCGTGT

ABL-a3-D 441 (23): TTCCCCATTGTGATTATAGCCTA.

The PCR reaction mixture in a final volume 50 µl consisting of 100 ρmoles of each primer, 10 mM dNTP mix, 1 U/ml Taq DNA Polymerase (Merck Biosciences), and 10X PCR assay buffer with 1.5 mM MgCl_2_ was used. The initial denaturation was performed at 95 °C for 3 min followed by 35 cycles of denaturation at 94 °C for 20 seconds; annealing at 55 °C for 15 seconds; extension at 72 °C for 15 seconds with a total 35 cycles by using Master cycler gradient Thermal cycler (Eppendorf). Final extension was performed at 72 °C for 5 min. After performing the PCR reaction, the amplified products were analyzed on 1.5% agarose gel electrophoresis^30,31^.

### Quantitative Real-time PCR (qRT-PCR) analysis

BCR-ABL transcript levels in peripheral blood were assessed using qRT-PCR. Patients with typical BCR-ABL transcripts b3a2 and b2a2 were eligible for molecular response analysis according to the suggested procedures and recommendations and the results were expressed as BCR-ABL/ABL ratio normalized according to international scale (IS)^32,33^.

### Mutation analysis

#### Kinase domain (KD) of BCR-ABL gene amplification

The KD of BCR-ABL gene was PCR amplified by using BCR forward primer: 5′-TGACCAACTCGTGTGTGAAACTC-3′ and ABL kinase reverse primer 5′-TCCACTTCGTCTGAGATACTGGATT-3′. A second-stage PCR was done by using ABL kinase forward primer: 5′-CGCAACAAGCCCACTGTCTATGG-3′ and ABL kinase reverse primer 5′-TCCACTTCGTCTGAGATACTGGATT-3′^3,4,8^. The PCR reaction mixture consisted of 100 ρmoles of each primer, 100 μmol of the dNTPs mix, 10 mM Tris- HCl (pH 8.8), 1.5 mM MgCl_2_, 1 U of Hot start Taq DNA Polymerase (Merck Biosciences Pvt, Ltd) and 2.5 μl of cDNA in a final volume of 25 μl. Amplification parameters included an initial denaturation step at 94 °C for 5 min and 35 cycles at 94 °C for 40 sec of denaturation, annealing at 56 °C for 40 sec, amplification at 72 °C for 60 sec, and final extension step at 72 °C for 5 min in a Master cycler gradient Thermal cycler (Eppendorf). The amplified PCR products were analyzed on 1.5% agarose gel electrophoresis^31^.

### Sequence analysis

#### Screening and identification of mutations

The amplified PCR products were purified and sequenced by dideoxy chain terminating method at Eurofins Genomics India Pvt Ltd^34^. The obtained nucleotide sequences were translated into protein sequence and compared with the BCR-ABL protein sequence from the NCBI database (ID: NM_005157.5). In order to identify the mutations, multiple sequence alignment was carried by using ClustalX software tool^35^.

### Structural analysis

#### Preparation and processing of wild-type and mutated ABL structures

All the *in silico* studies were carried out using the Molecular Operating Environment software tool (MOE 2011.10. Chemical Computing Group Inc). The ABL structure was retrieved from the Protein Data Bank (PDB ID: 1OPL) (http://www.rcsb.org/pdb/explore/explore.do?structureId=1OPL) with the resolution of 3.42 Å and was loaded into the MOE working environment ignoring all water molecules and heteroatoms. The structure was subjected to protonation followed by energy minimization under MMFF94x force field (Merck Molecular Force Field)^36,37^ to an RMSD of 0.05 where the implicit solvated environment was specified and the stabilized conformation was saved in PDB format. The mutations identified in the ABL gene were introduced individually in the wild-type energy minimized ABL protein, the resultant structures was again subjected to energy minimization. The energy minimization was done with the same parameter set and the stabilized conformation of the mutated ABL structures was saved individually in PDB file^36,37^.

### Molecular Dynamics Simulations (MD)

The stabilized conformations of both wild-type and mutated ABL structures were subjected to molecular dynamics simulations^36,38^ individually. To bring the system to perfect density or equilibration, 20 picoseconds (ps) and MMFF94x force field were applied for the MD simulations. The initial temperature was set to 30 K and increased to a runtime temperature of 300 K with a heat time of 10 ps and the pH of the system was adjusted to 7.0. The constraints were applied to the light bonds of the protein and the constraints on water molecules were set to be rigid^36,38^.

A final MD simulations run was carried out for 10 ns under NPT (Number of particles, Pressure, and Temperature) statistical ensemble where pressure and temperature were held fixed. The runtime temperature of 300 K and pressure of 100 kPa were set. NPA (Nose-Poincare-Anderson) algorithm was specified to solve the equations of motion, which was the most accurate and most sensitive method to generate true ensemble trajectories^36,38^. A constant temperature was maintained during the simulations with a bath relaxation time of 0.1 ps. A time step of 0.002 ps was specified to the equations of motion. A temperature response of 0.2 ps and a pressure response of 5 ps were specified to enforce the constant temperature and pressure respectively during MD simulations. At the end of simulations, the temperature was brought to 1 K with a cool time of 10 ps which was an additional duration to the main simulation time^36,38^.

### Molecular Docking study

Comparative molecular docking was performed for wild-type and mutated ABL structures to know the binding mode of the ligand and energy variations in the docking complexes. The optimized conformation of ligands- imatinib, nilotinib, bosutinib, and bafetinib was docked into the active site of ABL structure. The stabilized conformation of the substrate was used for flexible docking process and a systematic search was conducted covering all combinations of angles on the active site grid. Poses were generated by superposition of ligand atom triplets and triplets of receptor site points by triangle matcher docking placement methodology^36,38^. The receptor site points were alpha sphere centres, which represent locations of tight packing. A random conformation was selected at end of the docking, a random triplet of ligand atoms and a random triplet of alpha sphere centres were used to determine the pose. The resultant final docking conformation set was limited to 50. These conformations were ranked according to London dG scoring function which estimates the free energy of binding of the substrate from a given pose^36,38^. The conformation with the lowest docking score was chosen from each docking process for the study of binding mode orientations of imatinib, nilotinib, bosutinib and bafetinib in the active site cavity^36,38^.

### *PDBSum* conformational analysis

The energy stabilized wild-type and mutant ABL structures were analyzed for comprehensive structural variations through *PDBSum* analysis^39^. The conformational variations were measured and correlated for wild-type and mutated ABL structures in terms of helices, helix-helix interactions, β-turns, sheets, β-α-β units, hairpins, β-bulges, strands, and γ-turns.

### Structural superimposition of wild-type and mutated ABL structures

The PyMOL program was used to predict the wild-type and mutant ABL structural variations by superimposing to identify the structural variations especially in the domain and non-domain regions^40^. The wild-type and mutant ABL sequences were aligned and run in CLUSTALX software to know the sequence variations.

### Polymorphism Phenotyping (PolyPhen) and Sift score calculation

The Bioinformatics tools like PolyPhen-2 (Polymorphism Phenotyping V2) (http://genetics.bwh.harvard.edu/pph2/)^41^ and the Sift (Sorting Intolerant From Tolerant) score (http://sift.bii.a-star.edu.sg/)^42^ online web servers were used to predict the impact of the stability and function of novel mutations identified on the BCR-ABL protein in CML patients.

### Statistical analysis

The statistical significance was performed using MS office excel and ANOVA where p < 0.05^43^.

## Supplementary information

Supplementary Information

## References

[1] Ben-Neriah Y (1986). The chronic myelogenous leukemia- specific P210 protein is the product of the bcr/abl hybrid gene. Science..

[2] Elias MH (2014). BCR-ABL kinase domain mutations, including 2 novel mutations in imatinib resistant Malaysian chronic myeloid leukemia patients-Frequency and clinical outcome. Leuk Res..

[3] Branford S (2002). High frequency of point mutations clustered within the adenosine triphosphate-binding region of BCR/ABL in patients with chronic myeloid leukemia or Ph-positive acute lymphoblastic leukemia who develop imatinib (STI571) resistance. Blood..

[4] Branford S (2003). Detection of BCR-ABL mutations in patients with CML treated with imatinib is virtually always accompanied by clinical resistance, and mutations in the ATP phosphate-binding loop (P-loop) are associated with a poor prognosis. Blood..

[5] Jackson P, Baltimore D (1989). N-terminal mutations activate the leukemogenic potential of the myristoylated form of c-abl. EMBO J..

[6] Nam HJ (1996). Intra molecular interactions of the regulatory domains of the Bcr-Abl kinase reveal a novel control mechanism. Structure..

[7] Daley GQ, Van Etten RA, Baltimore D (1990). Induction of chronic myelogenous leukemia in mice by the P210bcr/abl gene of the Philadelphia chromosome. Science..

[8] Willis SG (2005). High-sensitivity detection of BCR-ABL kinase domain mutations in imatinib-naive patients: correlation with clonal cytogenetic evolution but not response to therapy. Blood..

[9] Musacchio A, Saraste M, Wilmanns M (1994). High-resolution crystal structures of tyrosine kinase SH3 domains complexed with proline rich peptides. Nat Struct Biol..

[10] Overduin M (1992). Three-dimensional solution structure of the Src homology 2 domain of c-Abl. Cell..

[11] Vaidya S (2015). Evolution of BCR/ABL gene mutation in CML is time dependent and dependent on the pressure exerted by tyrosine kinase inhibitor. PLoS One..

[12] Milojkovic D, Apperley J (2009). Mechanisms of Resistance to Imatinib and Second-Generation Tyrosine Inhibitors in Chronic Myeloid Leukemia. Clin Cancer Res..

[13] Baccarani M (2009). Chronic myeloid leukemia: an update of concepts and management recommendations of European LeukemiaNet. J Clin Oncol..

[14] Stagno F (2012). Hyperdiploidy associated with a high BCR-ABL transcript level may identify patients at risk of progression in chronic myeloid leukemia. Acta Haematol..

[15] Ma W (2009). BCR-ABL truncation due to premature translation termination as a mechanism of resistance to kinase inhibitors. Acta Haematol..

[16] Kimura S, Ando T, Kojima K (2014). BCR-ABL Point Mutations and TKI Treatment in CML Patients. J Hematol Transfus..

[17] Deb P (2014). Incidence of BCR-ABL transcript variants in patients with chronic myeloid leukemia: Their correlation with presenting features, risk scores and response to treatment with imatinib mesylate. Indian J Med Paediatr Oncol..

[18] Anand MS (2012). Cytogenetic & molecular analyses in adult chronic myelogenous leukaemia patients in north India. Indian J Med Res..

[19] Iqbal Z (2014). A comprehensive analysis of breakpoint cluster region-abelson fusion oncogene splice variants in chronic myeloid leukemia and their correlation with disease biology. Indian J Hum Genet..

[20] Kagita S (2018). Assessment of BCR-ABL1 fusion transcripts and their association with response to imatinib treatment in chronic myeloid leukemia patients. Indian J Med Paediatr Oncol..

[21] Osman EA (2010). Frequencies of BCR-ABL1 fusion transcripts among Sudanese chronic myeloid leukaemia patients. Genet Mol Biol..

[22] Sharma P (2010). Response to Imatinib mesylate in chronic myeloid leukemia patients with variant BCR-ABL fusion transcripts. Ann Hematol..

[23] Latagliata R (2011). Dasatinib is safe and effective in unselected chronic myeloid leukaemia elderly patients resistant/intolerant to imatinib. Leuk Res..

[24] Allen PB, Wiedemann LM (1996). An activating mutation in the ATP binding site of the ABL kinase domain. J Biol Chem..

[25] Yuan H (2010). BCR-ABL gene expression is required for its mutations in a novel KCL-22 cell culture model for acquired resistance of chronic myelogenous leukemia. J Biol Chem..

[26] Barnes DJ (2005). Bcr-Abl expression levels determine the rate of development of resistance to imatinib mesylate in chronic myeloid leukemia. Cancer Res..

[27] Shaffer, L. G., Slovak, M. L. & Campbell, L. J. ISCN 2009: An International System for Human Cytogenetic Nomenclature. *S*. *Karger AG*, *Basel* (2009).

[28] Joshi D (2014). Down-regulation of miR-199b associated with imatinib drug resistance in 9q34.1 deleted BCR/ABL positive CML patients. Gene..

[29] Kumar PS (2018). *In vitro* large scale production of megakaryocytes to functional platelets from human hematopoietic stem cells. Biochem Biophys Res Commun..

[30] van Dongen JJ (1999). Standardized RT-PCR analysis of fusion gene transcripts from chromosome aberrations in acute leukemia for detection of minimal residual disease. Report of the BIOMED-1 Concerted Action: investigation of minimal residual disease in acute leukemia. Leukemia..

[31] Pasupuleti SK (2014). Novel frame shift mutations (‘A’ deletion) observed in exon 9 of Wilms’ tumor (WT1) gene in a patient reported with glomerulosclerosis. Gene..

[32] Branford S (2008). Desirable performance characteristics for BCR-ABL measurement on an international reporting scale to allow consistent interpretation of individual patient response and comparison of response rates between clinical trials. Blood..

[33] Hughes T (2006). Monitoring CML patients responding to treatment with tyrosine kinase inhibitors: review and recommendations for harmonizing current methodology for detecting BCR-ABL transcripts and kinase domain mutations and for expressing results. Blood..

[34] Babu PP (2017). Novel mutations in the exon 5, intron 2 and 3′ UTR regions of IL-12B gene were observed in clinically proven tuberculosis patients of south India. Cytokine..

[35] Thompson JD, Gibson TJ, Plewniak F (1997). The CLUSTAL_X windows interface: flexible strategies for multiple sequence alignment aided by quality analysis tools. Nucleic Acids Res..

[36] Yellapu NK (2014). Identification and analysis of novel R308K mutation in glucokinase of type 2 diabetic patient and its kinetic correlation. Biotechnol Appl Biochem..

[37] Kumar PS (2014). *In silico* designing and molecular docking of a potent analog against *Staphylococcus aureus* porphobilinogen synthase. J Pharm Bioallied Sci..

[38] Kumar PS (2015). Mutations in exons 3 and 7 resulting in truncated expression of human ATP6V1B1 gene showing structural variations contributing to poor substrate binding-causative reason for distal renal tubular acidosis with sensorineural deafness. J Biomol Struct Dyn..

[39] Laskowski RA (2001). PDBsum: Summaries and analyses of PDB structures. Nucleic Acids Res..

[40] Bramucci E (2012). PyMod: sequence similarity searches, multiple sequence-structure alignments, and homology modeling within PyMOL. BMC Bioinformatics..

[41] Adzhubei, I., Jordan, D. M., & Sunyaev, S. R. Predicting functional effect of human missense mutations using PolyPhen-2. Curr Protoc Hum Genet, Chapter 7: Unit7.20 (2013). 10.1002/0471142905.hg0720s76 PMC4480630 23315928

[42] Ng PC, Henikoff S (2003). SIFT: Predicting amino acid changes that affect protein function. Nucleic Acids Res..

[43] Kumar BS (2016). Identification of novel mutations in CD2BP1 gene in clinically proven rheumatoid arthritis patients of south India. Eur J Med Genet..

