# Supplementary material for: Novel mutations in the kinase domain of BCR-ABL gene causing imatinib resistance in chronic myeloid leukemia patients
Source: Sci Rep. 2019 Feb 20;9:2412. doi: 10.1038/s41598-019-38672-x (PMC6382822; doi:10.1038/s41598-019-38672-x)
Supplement: Supplementary file 1 — Supplementary Information [file 41598_2019_38672_MOESM1_ESM.pdf]

Supplementary Information

Novel mutations in the kinase domain of BCR-ABL gene cause imatinib resistance in chronic myeloid leukemia patients

Chodimella Chandrasekhar, Pasupuleti Santhosh Kumar, Potukuchi Venkata Gurunadha Krishna Sarma

Inventory of Supplementary Information

Supplementary Table 1

Supplementary Figures 1-64

Supplementary Table 1. Superimposition of wild-type ABL structure (Green color) and mutated (Different colors) ABL structures.

Mutated ABL	Superimposed structure	RMSD (Å)
p.(Glu281*)	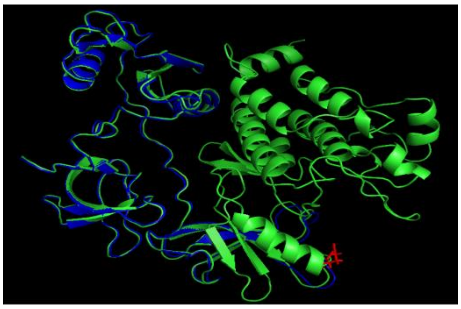	5.036
p.(Tyr393*)	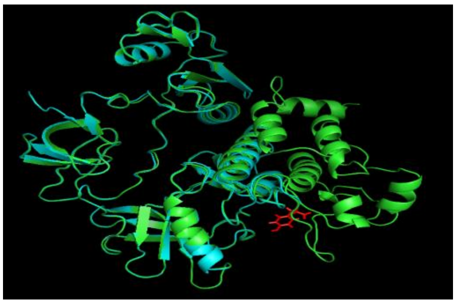	2.459
p.(Asp381Asn)	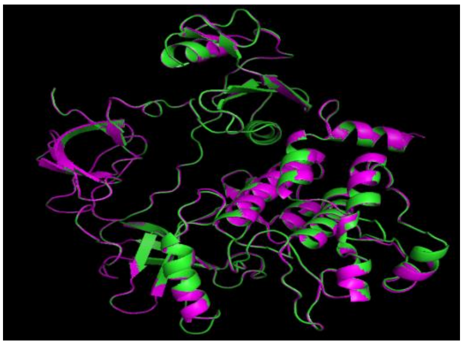	1.395
p.(Glu373Asp)	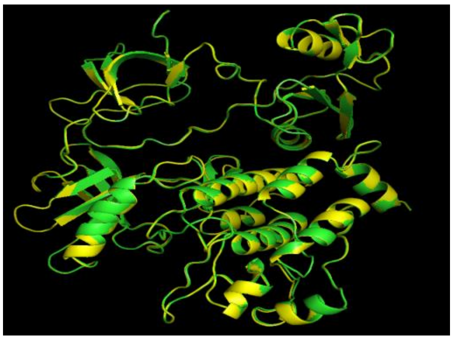	1.489
p.(Phe317Leu)	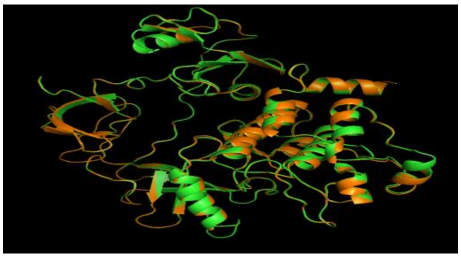	1.852

p.(Gly250Glu)	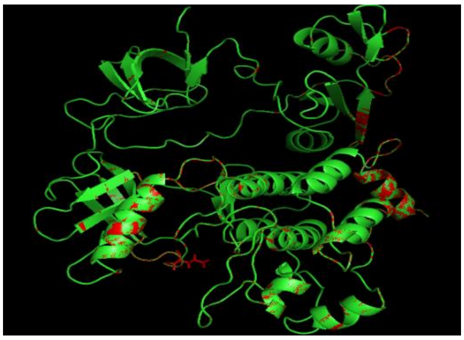	0.867
p.(Leu301Ile)	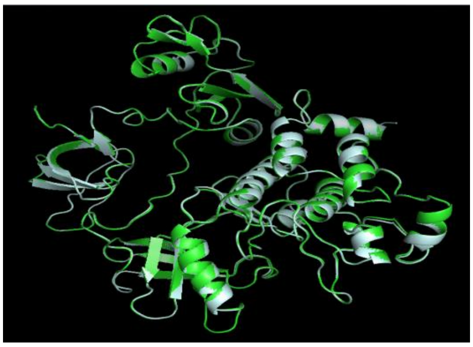	1.539
p.(Met351Thr)	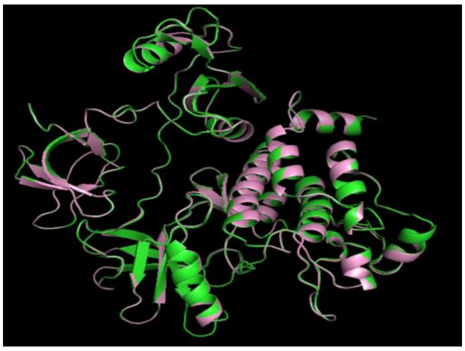	3.142
p.(Thr315Ile)	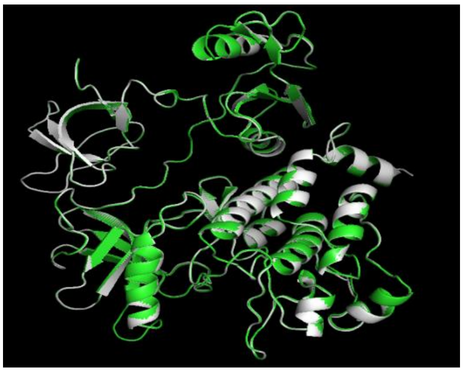	3.146
p.(Val256Gly)	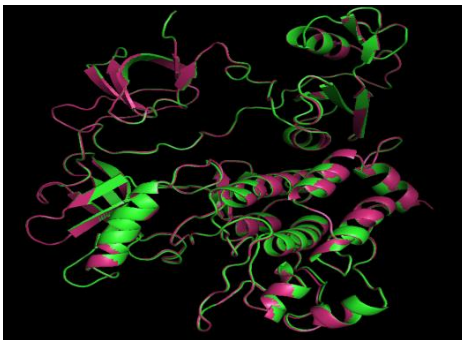	4.145

p.(Tyr253His)	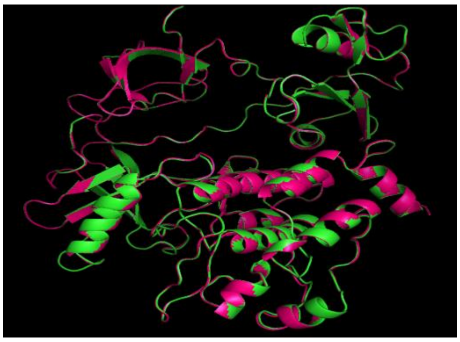	1.538
p.(Tyr320His)	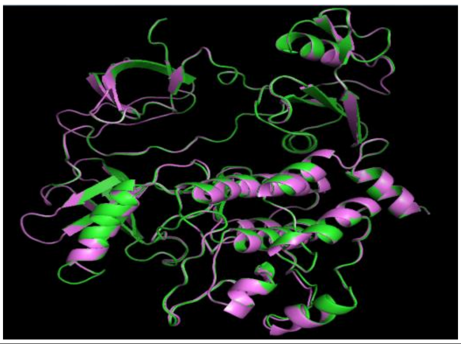	1.615

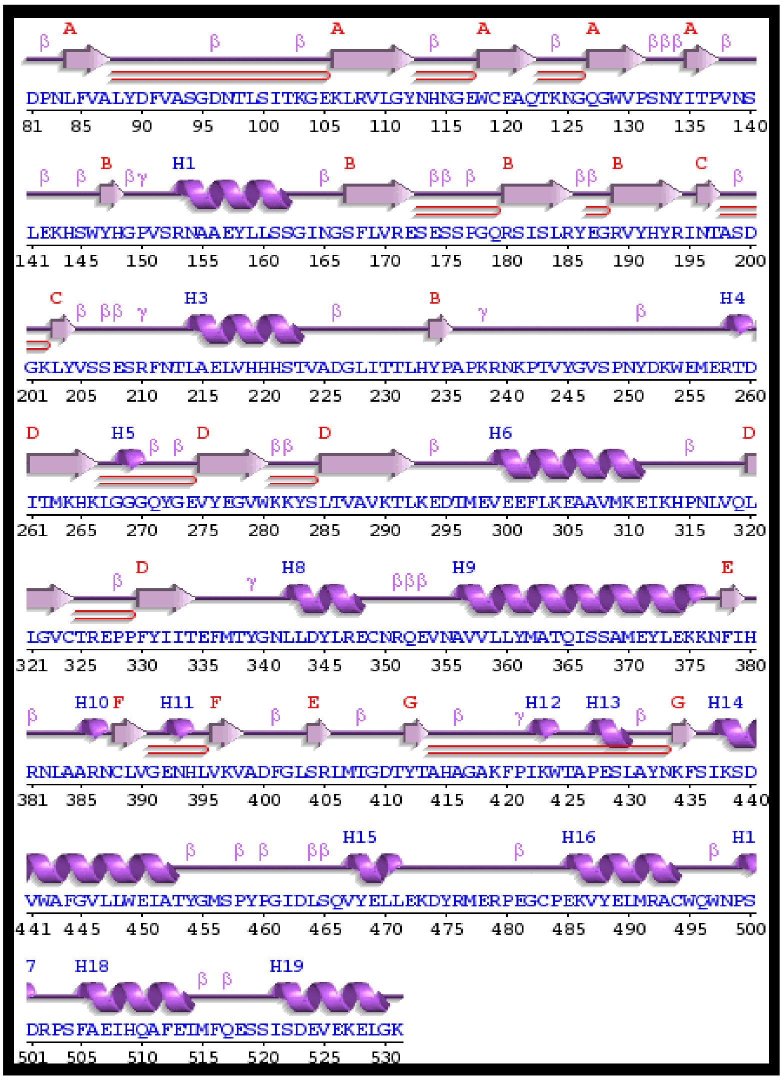

Supplementary Figure S1: Secondary structural conformation of wild-type ABL structure.

Keys:

Secondary structure: Helices labeled as H1, H2, and strands by their sheets A, B.

HelixStrand

Motifs: β beta turn γ gamma turn beta hairpin

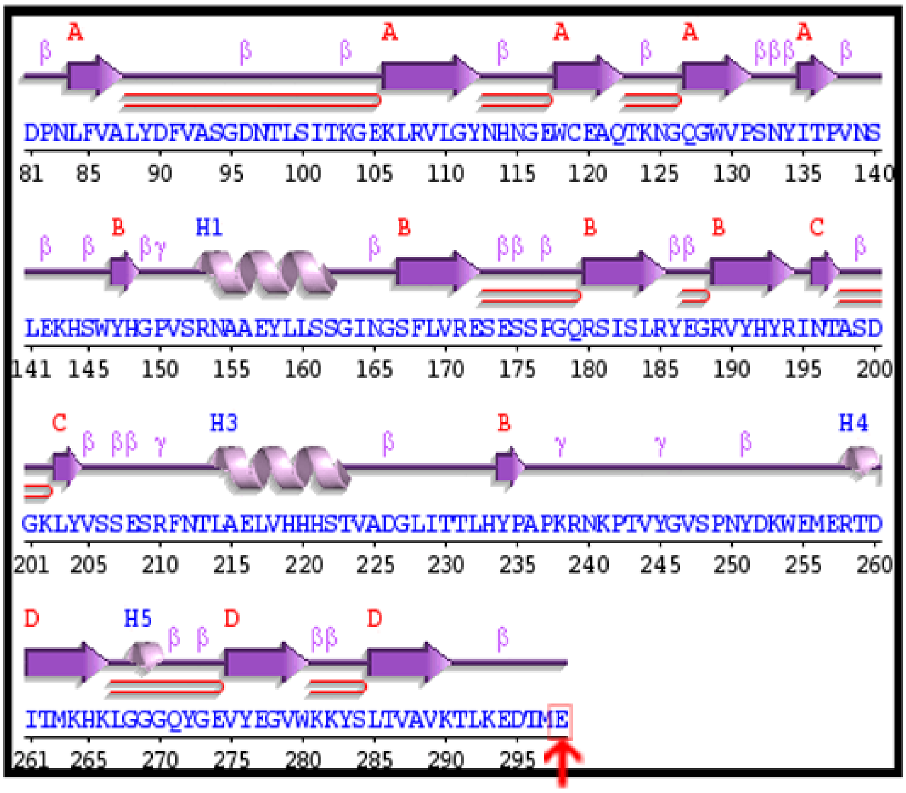

Supplementary Figure S2: Secondary structural conformation of mutant p.(Glu281*) ABL structure. The mutated region p.(Glu281*) was found to be localized in H5 helix (indicated in red arrow).

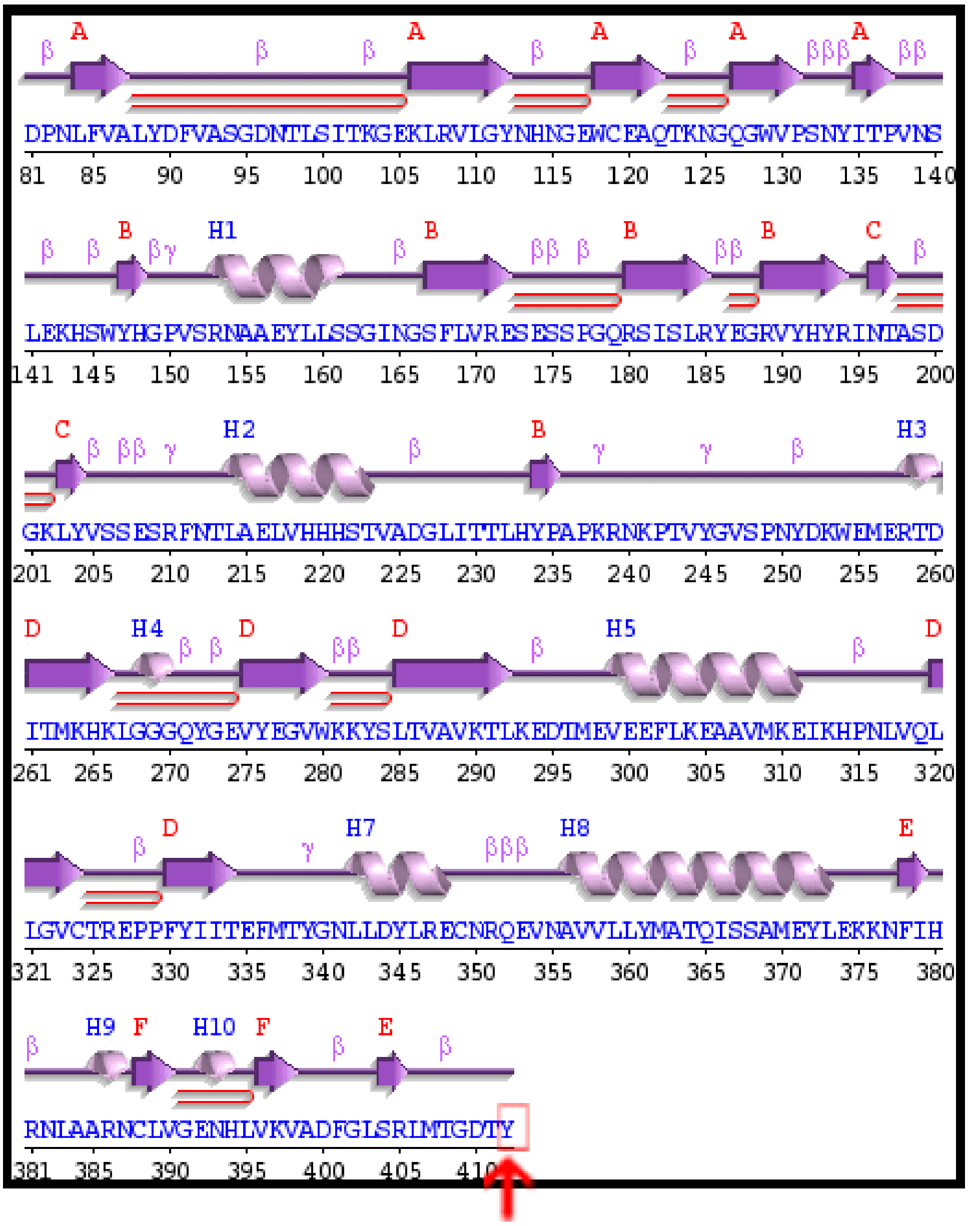

Supplementary Figure S3: Secondary structural conformation of mutant p.(Tyr393*) ABL structure. The mutated region p.(Tyr393*) was found to be localized in H10 helix (indicated in red arrow).

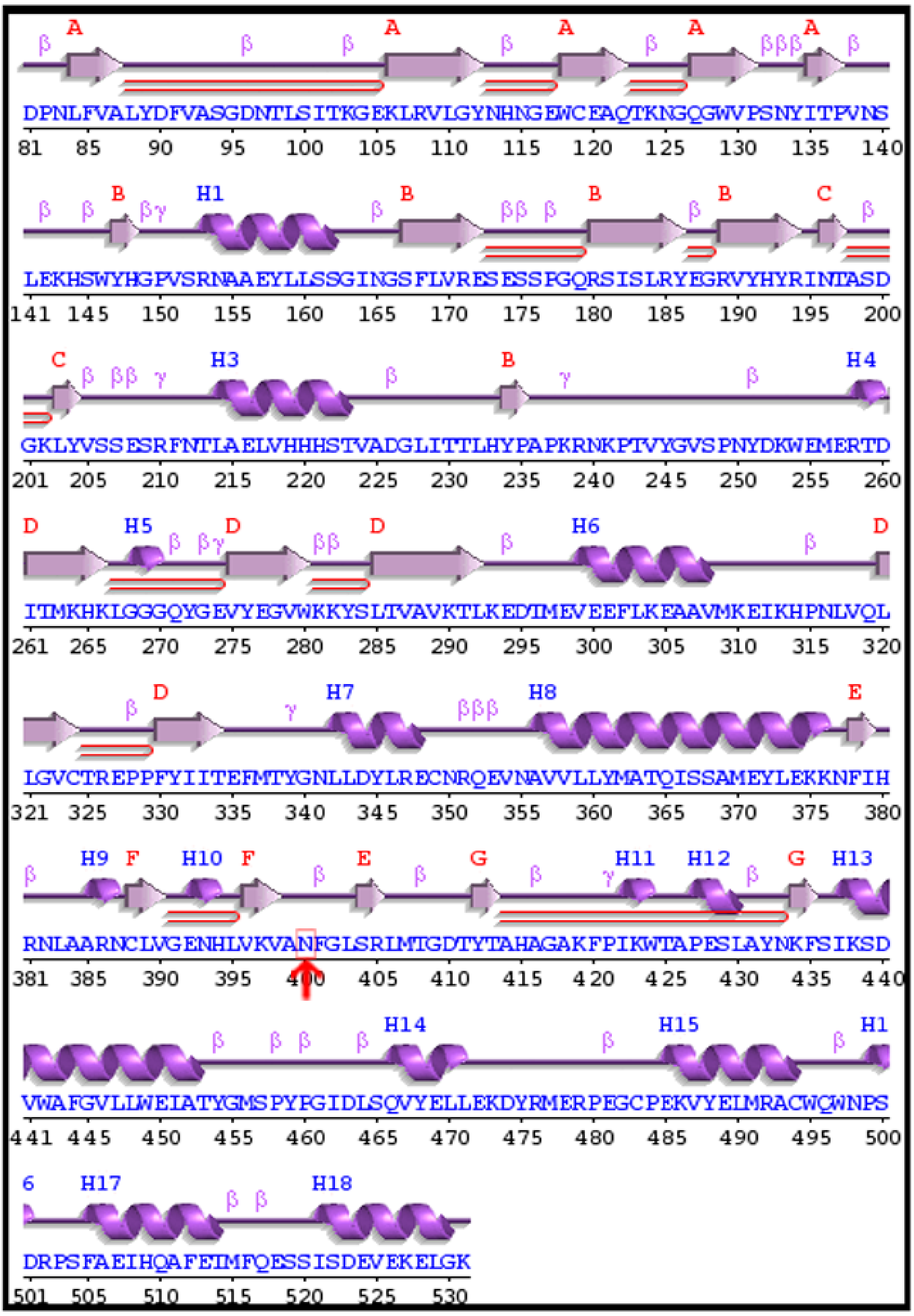

Supplementary Figure S4: Secondary structural conformation of mutant p.(Asp381Asn) ABL structure. The mutated region p.(Asp381Asn) was found to be localized in H10 helix (indicated in red arrow).

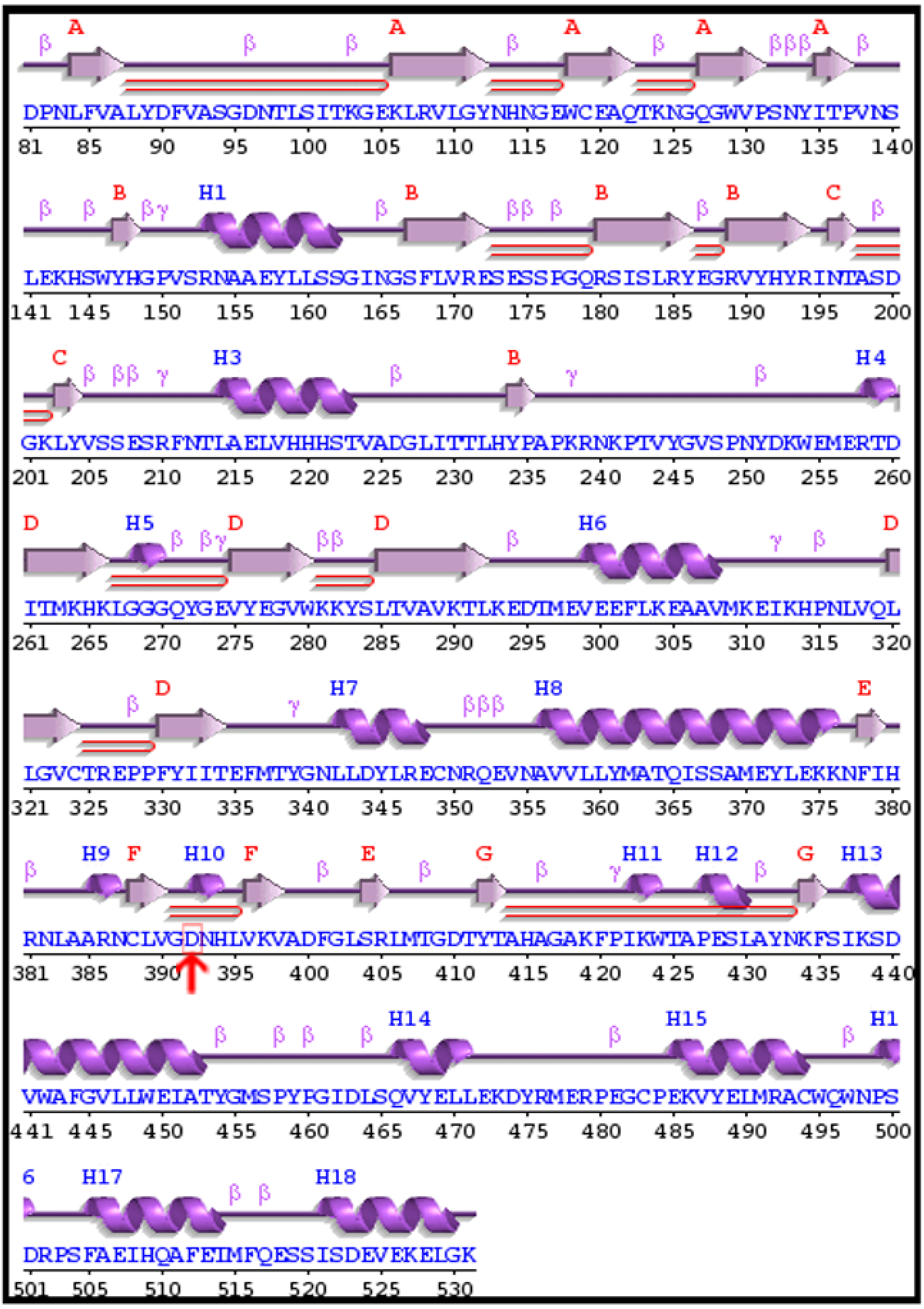

Supplementary Figure S5: Secondary structural conformation of mutant p.(Glu373Asp) ABL structure. The mutated region p.(Glu373Asp) was found to be localized in H10 helix (indicated in red arrow).

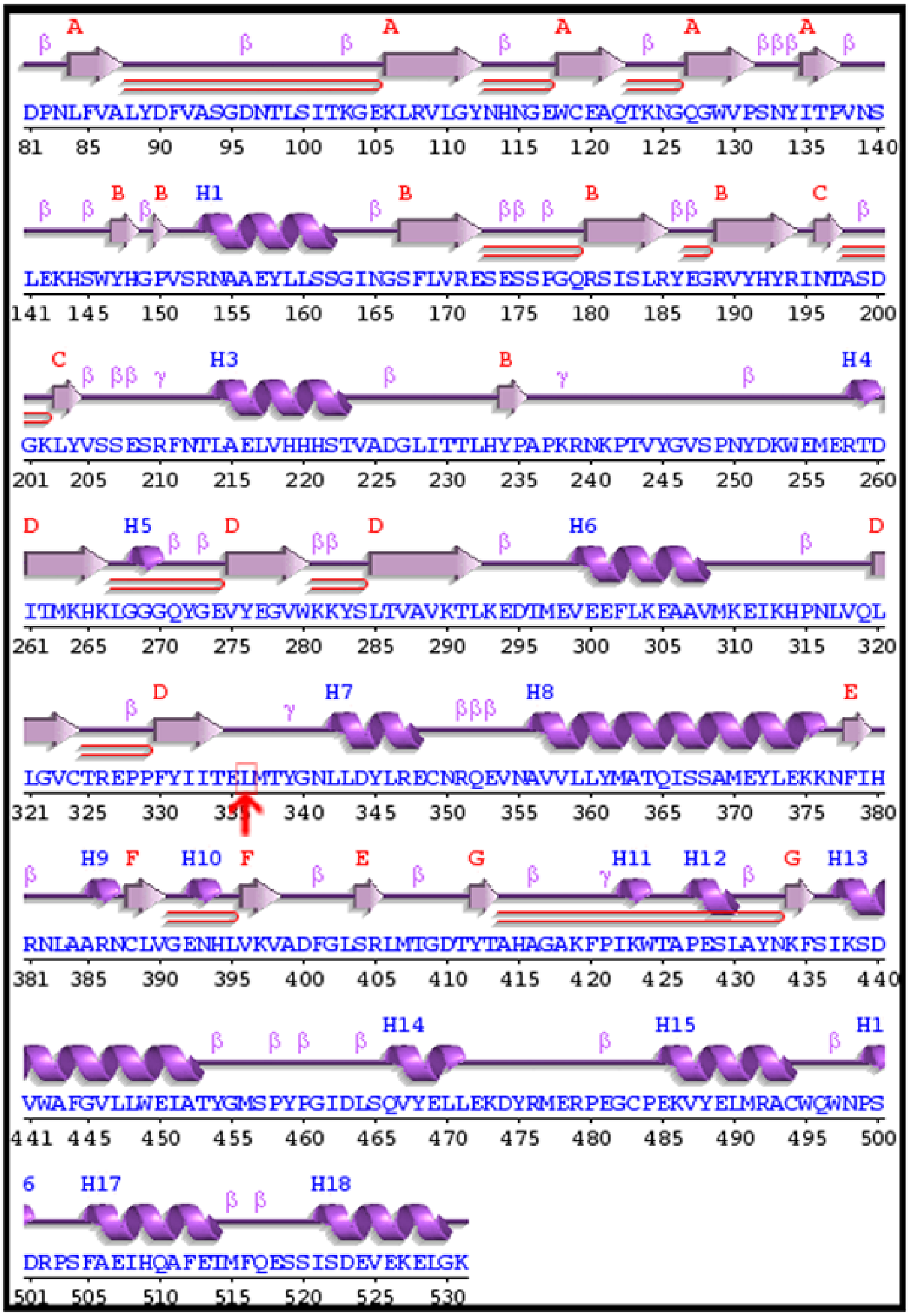

Supplementary Figure S6: Secondary structural conformation of mutant p.(Phe317Leu) ABL structure. The mutated region p.(Phe317Leu) was found to be localized in H6 helix (indicated in red arrow).

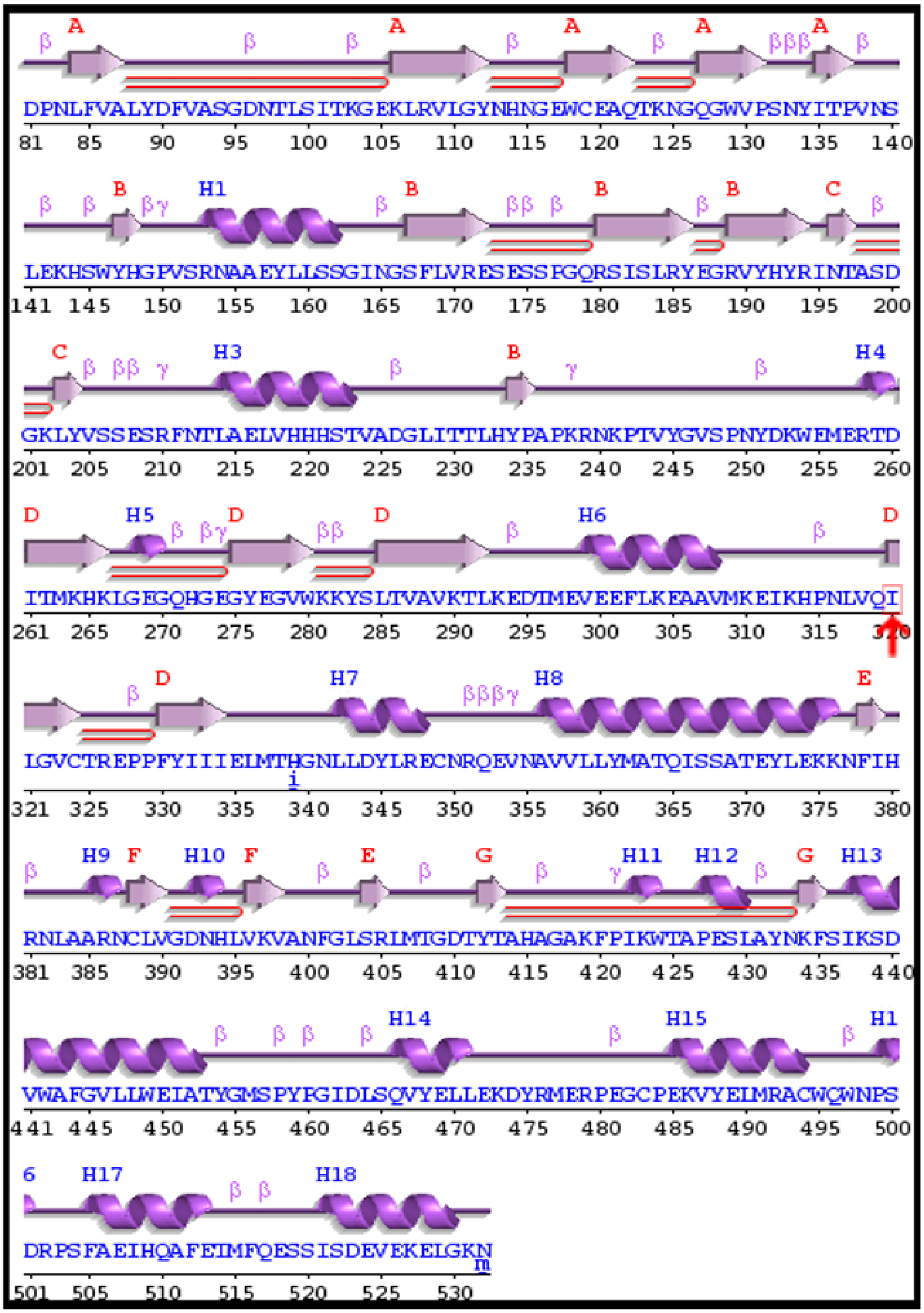

Supplementary Figure S7: Secondary structural conformation of mutant p.(Leu301Ile) ABL structure. The mutated region p.(Leu301Ile) was found to be localized in H6 helix (indicated in red arrow).

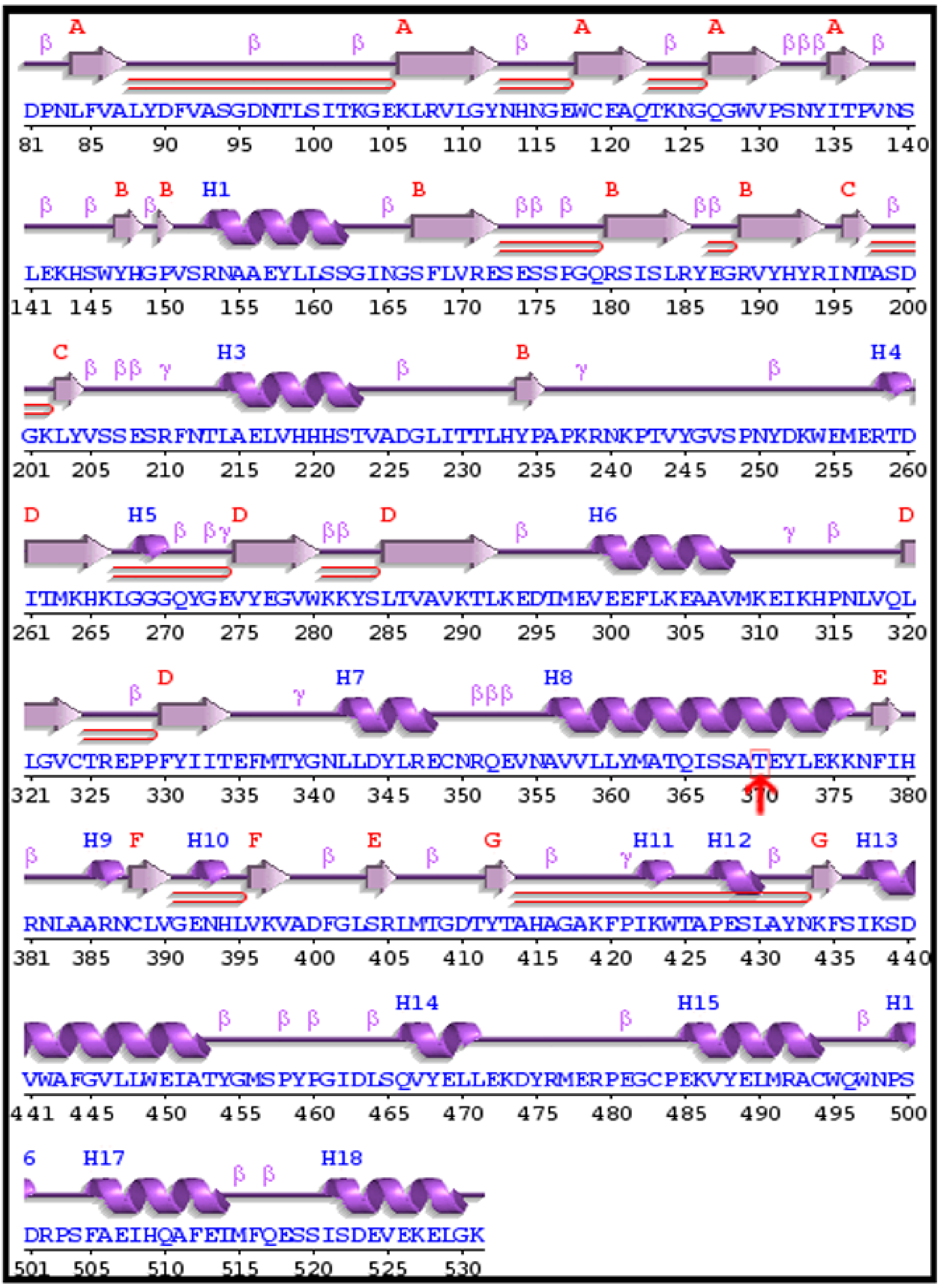

Supplementary Figure S8: Secondary structural conformation of mutant p.(Met351Thr) ABL structure. The mutated region p.(Met351Thr) was found to be localized in H8 helix (indicated in red arrow).

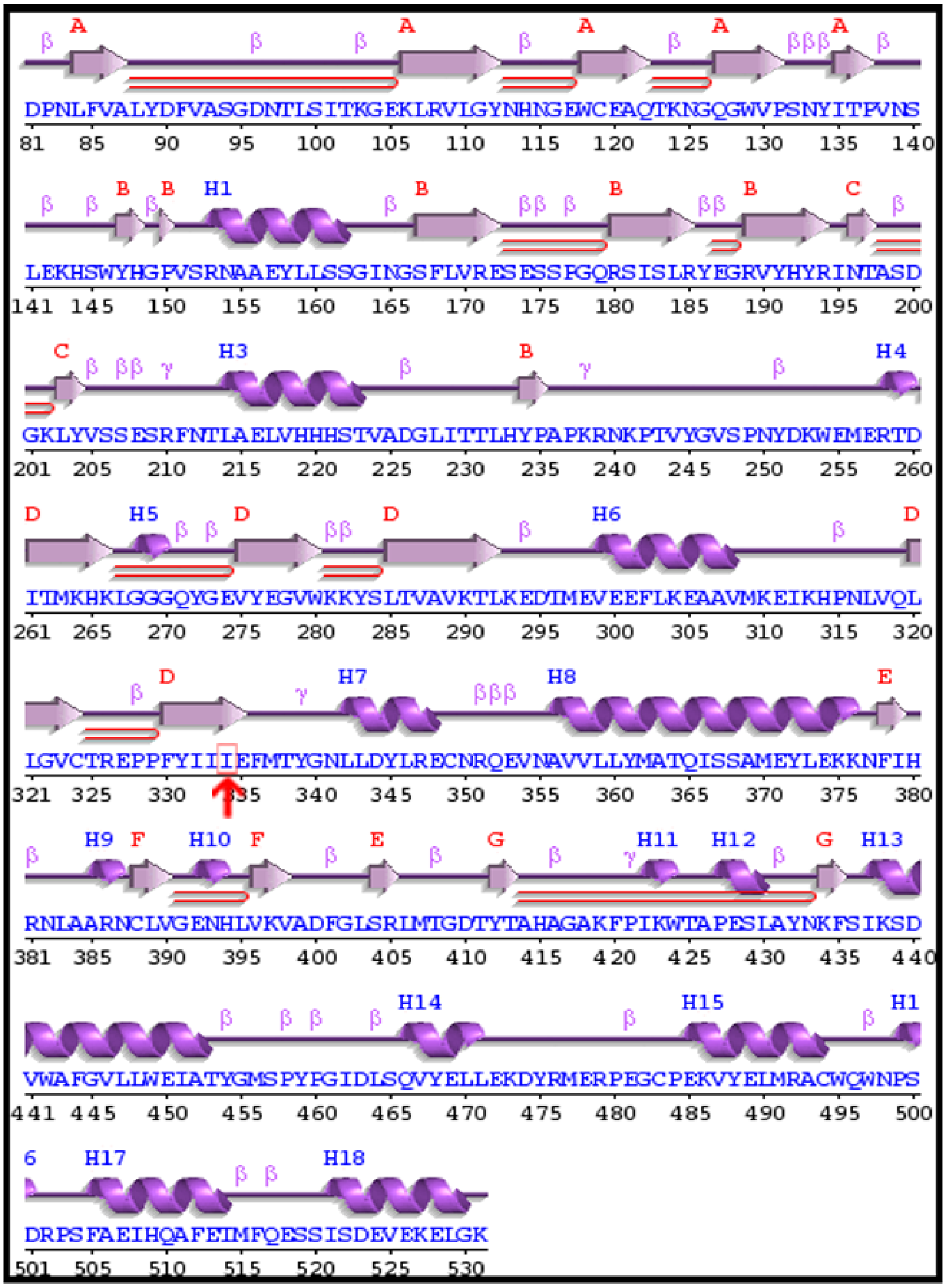

Supplementary Figure S9: Secondary structural conformation of mutant p.(Thr315Ile) ABL structure. The mutated region p.(Thr315Ile) was found to be localized in H6 helix (indicated in red arrow).

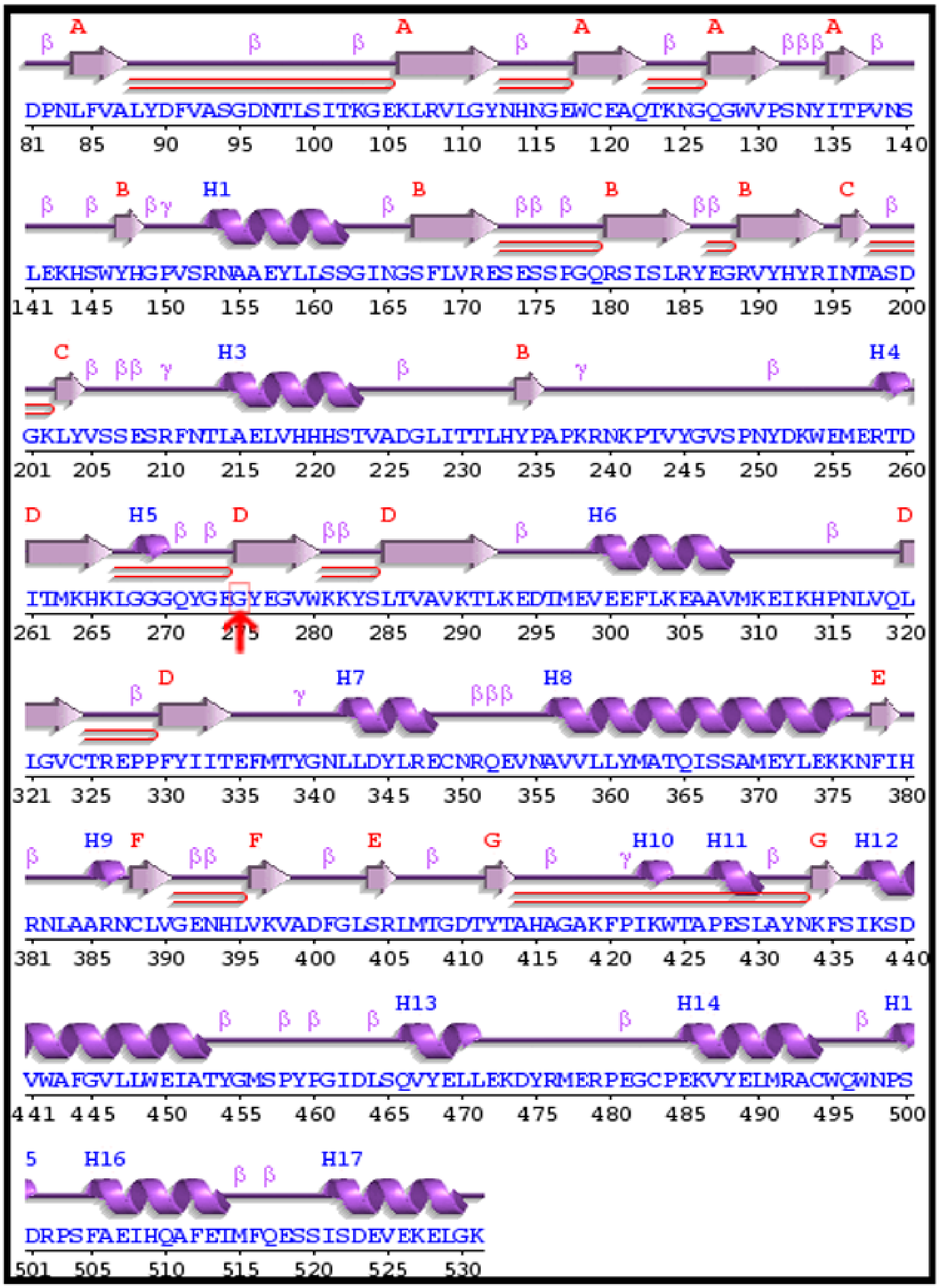

Supplementary Figure S10: Secondary structural conformation of mutant p.(Val256Gly) ABL structure. The mutated region p.(Val256Gly) was found to be localized in H5 helix (indicated in red arrow).

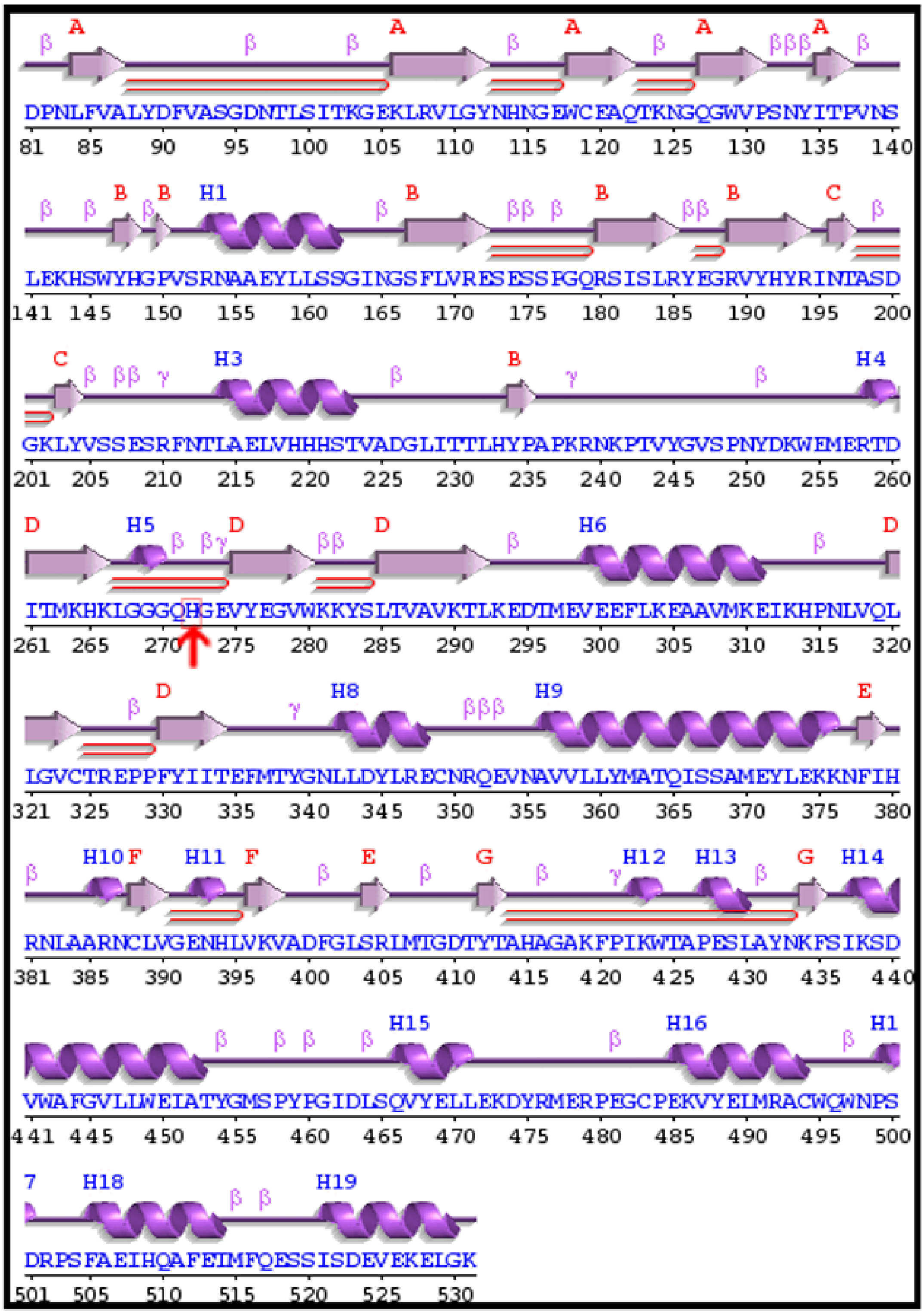

Supplementary Figure S11: Secondary structural conformation of mutant p.(Tyr253His) ABL structure. The mutated region p.(Tyr253His) was found to be localized in H5 helix (indicated in red arrow).

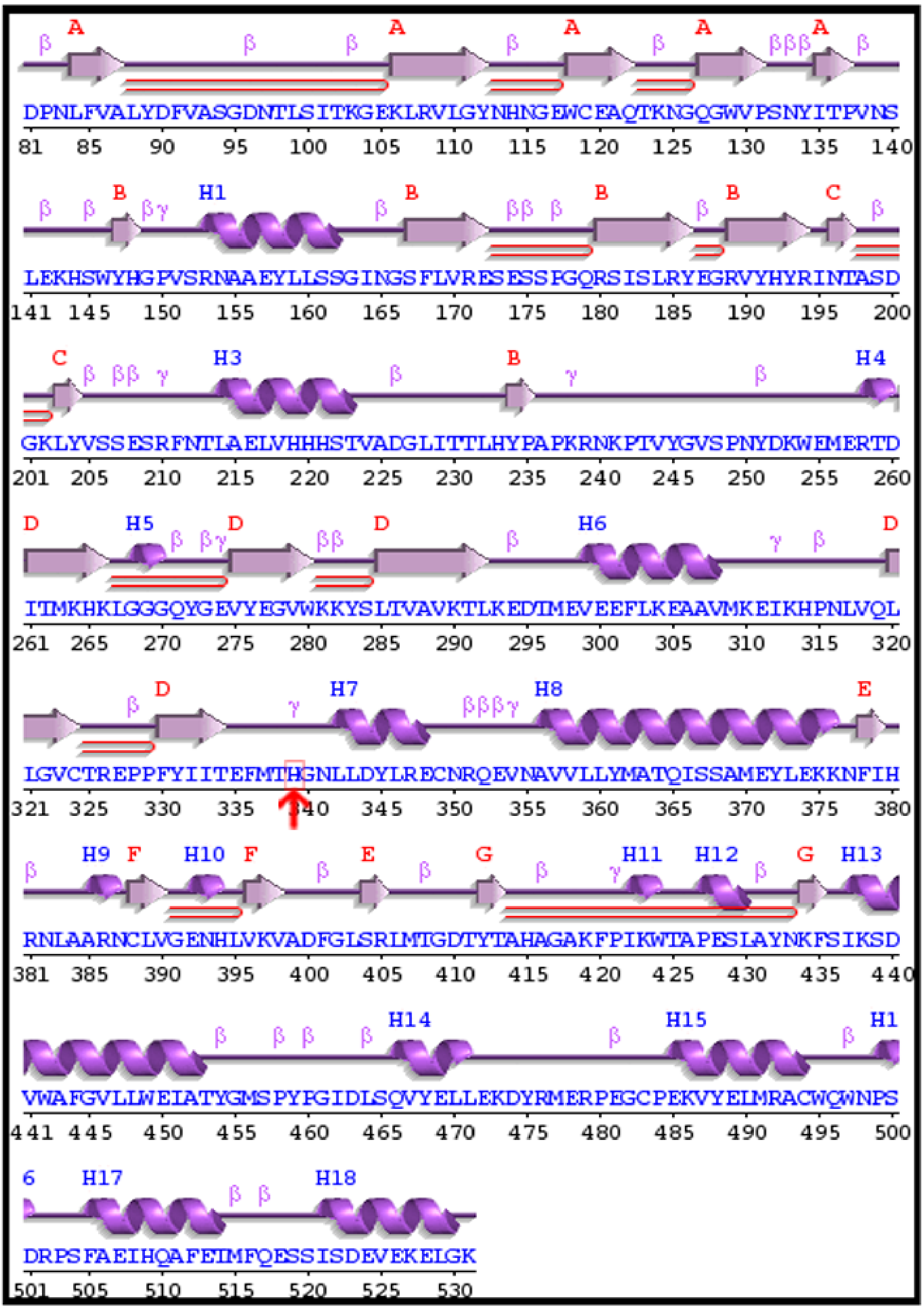

Supplementary Figure S12: Secondary structural conformation of p.(Tyr320His) ABL structure. The mutated region p.(Tyr320His) was found to be localized in H6 helix (indicated in red arrow).

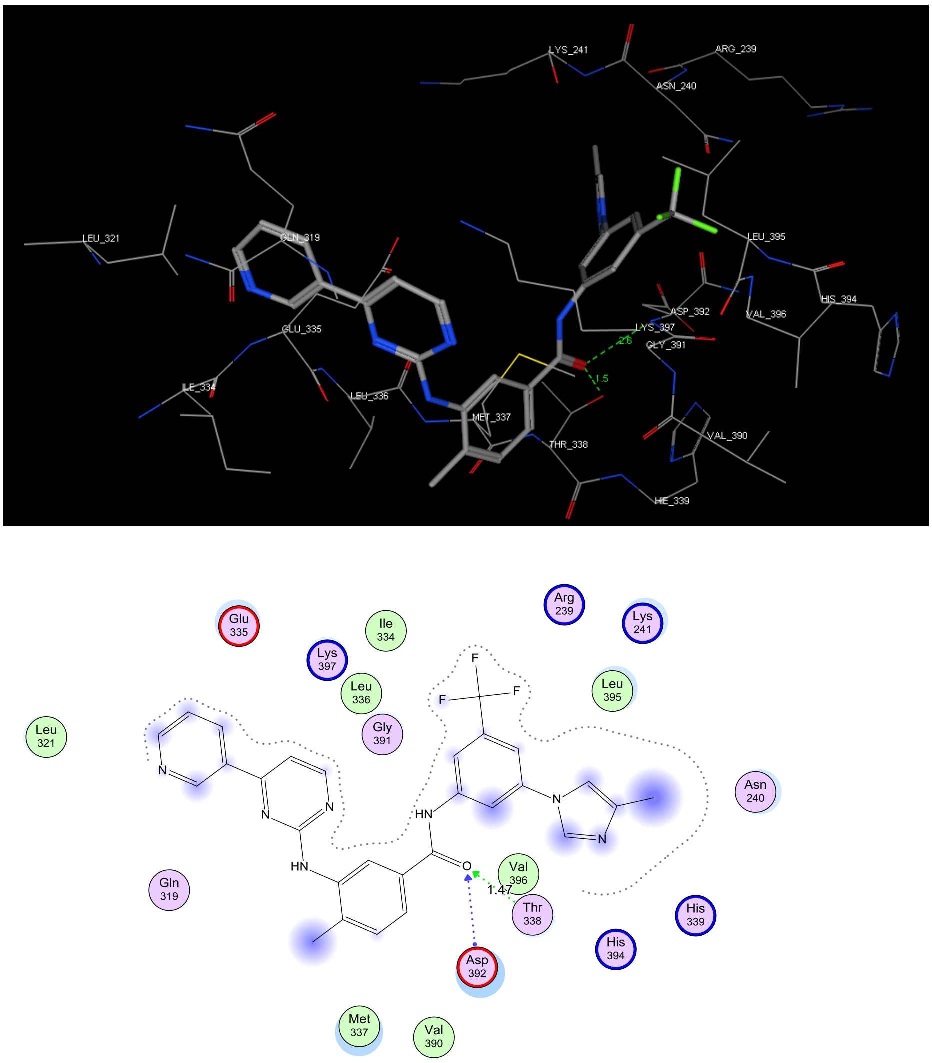

Supplementary Figure S13. Docking of Wild-type ABL structure with Nilotinib.

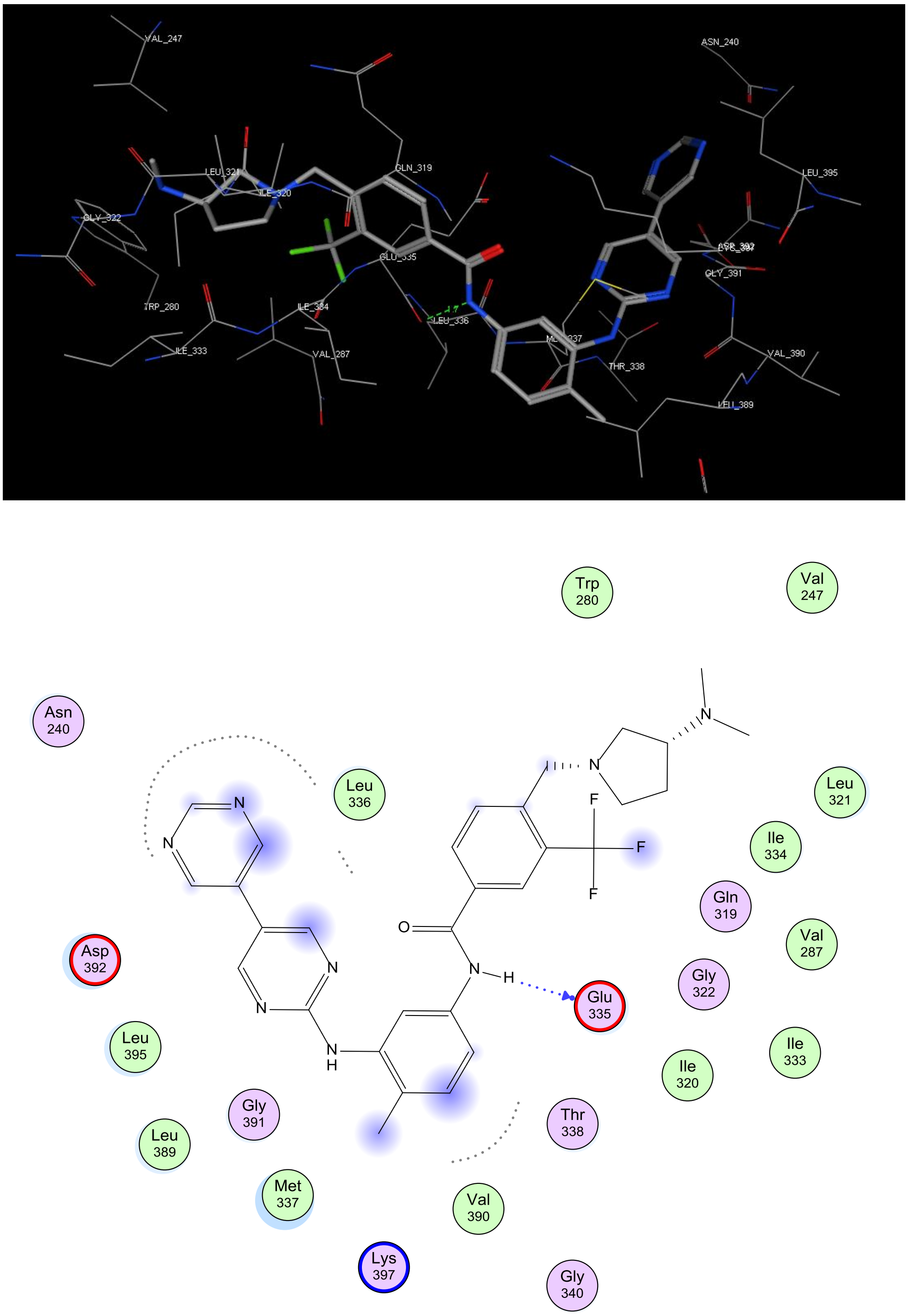

Supplementary Figure S14. Docking of Wild-type ABL structure with Bafetinib.

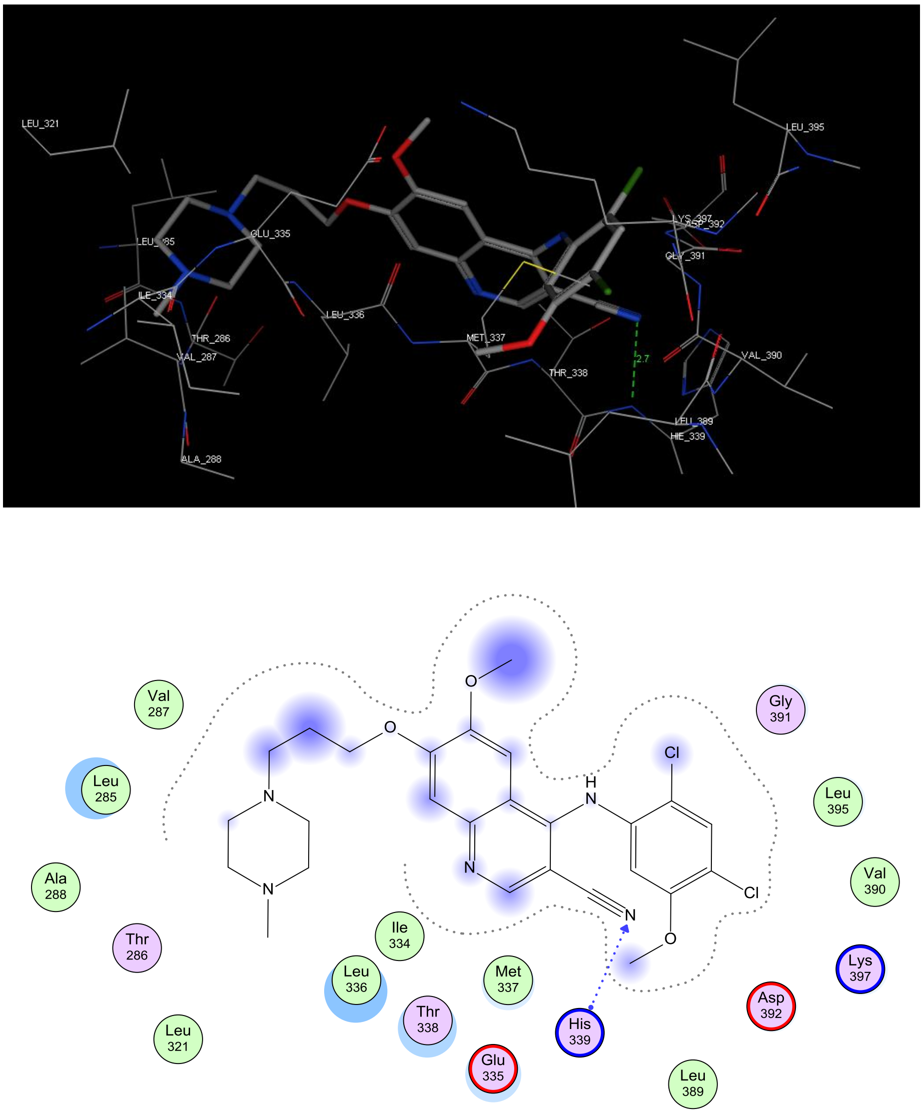

Supplementary Figure S15. Docking of Wild-type ABL structure with Bosutinib.

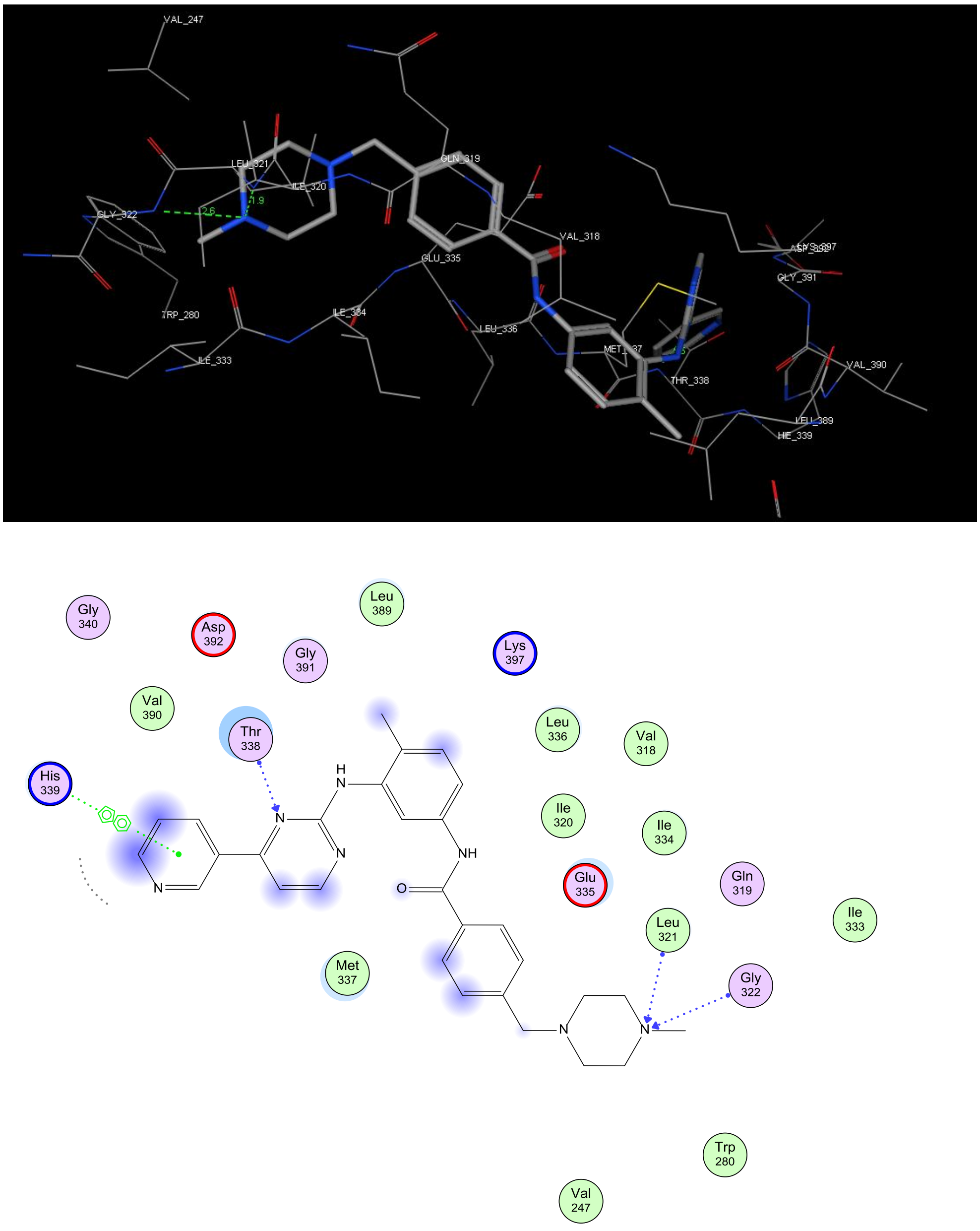

Supplementary Figure S16. Docking of Wild-type ABL structure with Imatinib.

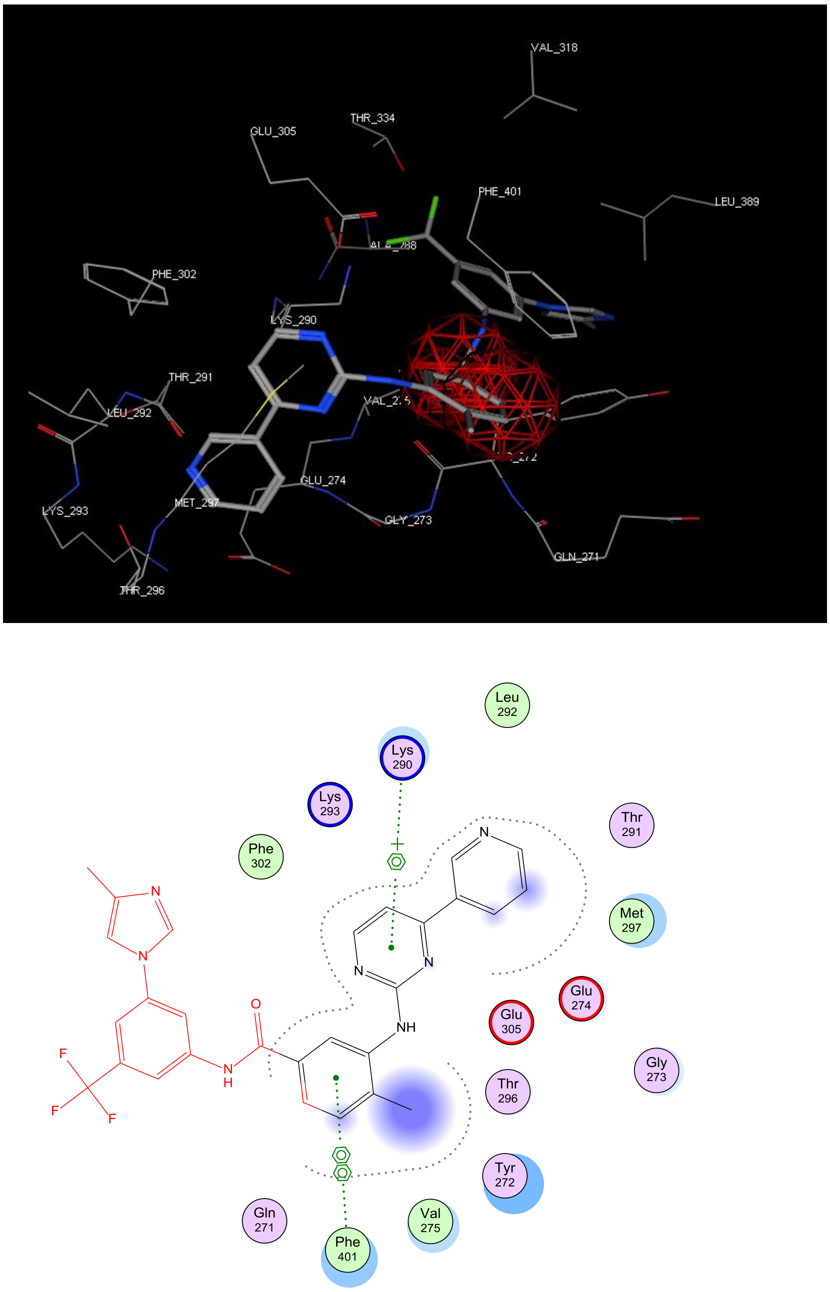

Supplementary Figure S17. Docking of p.(Asp381Asn) mutant ABL structure with Nilotinib.

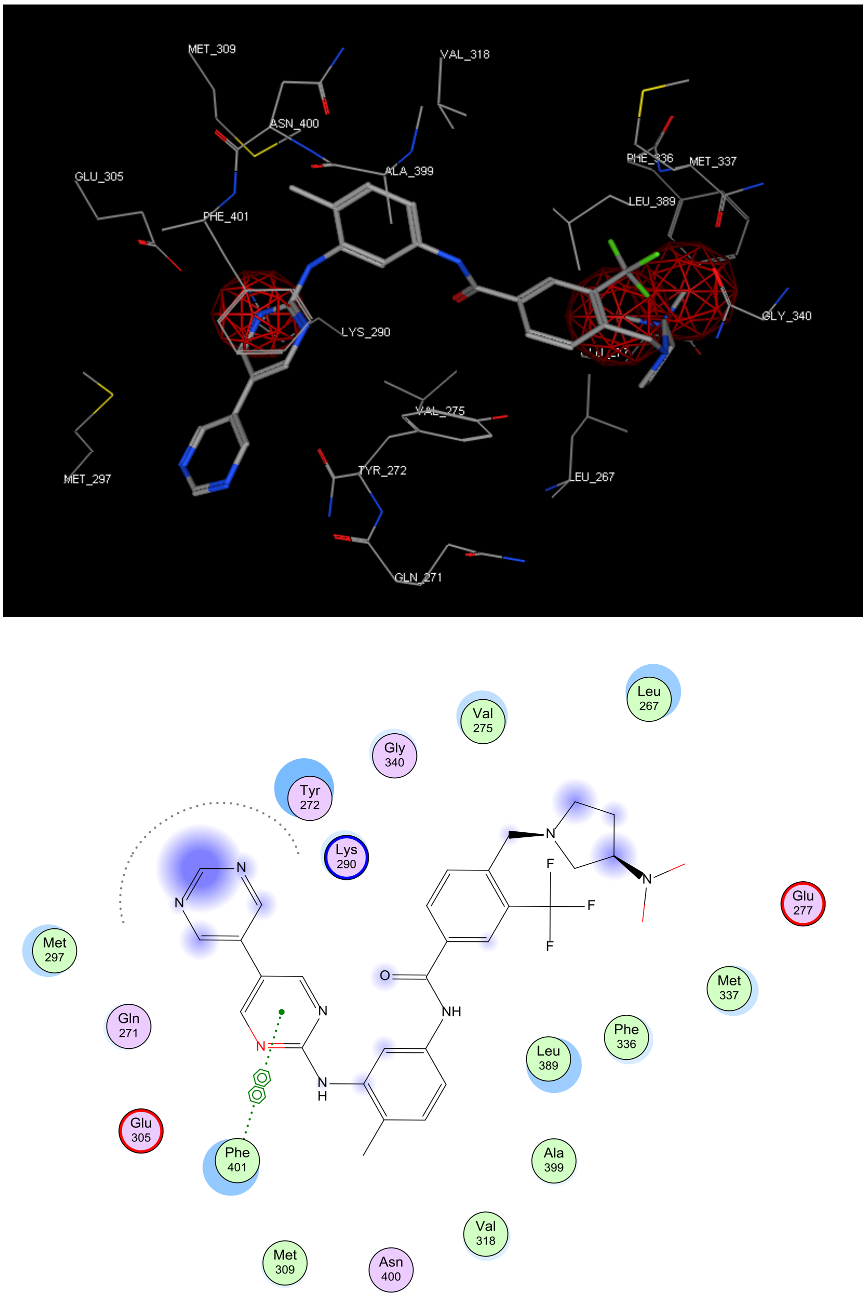

Supplementary Figure S18. Docking of p.(Asp381Asn) mutant ABL structure with Bafetinib.

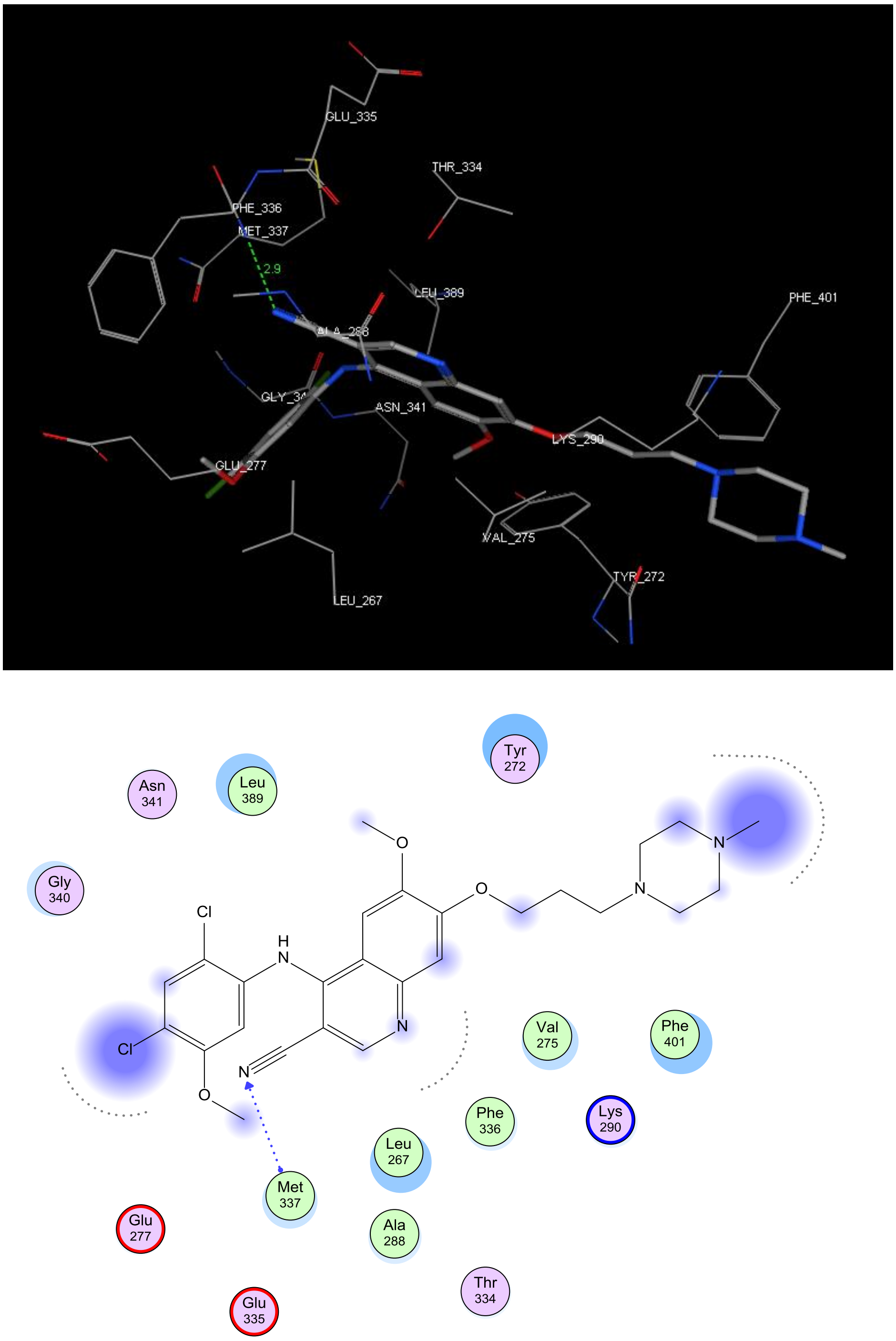

Supplementary Figure S19. Docking of p.(Asp381Asn) mutant ABL structure with Bosutinib.

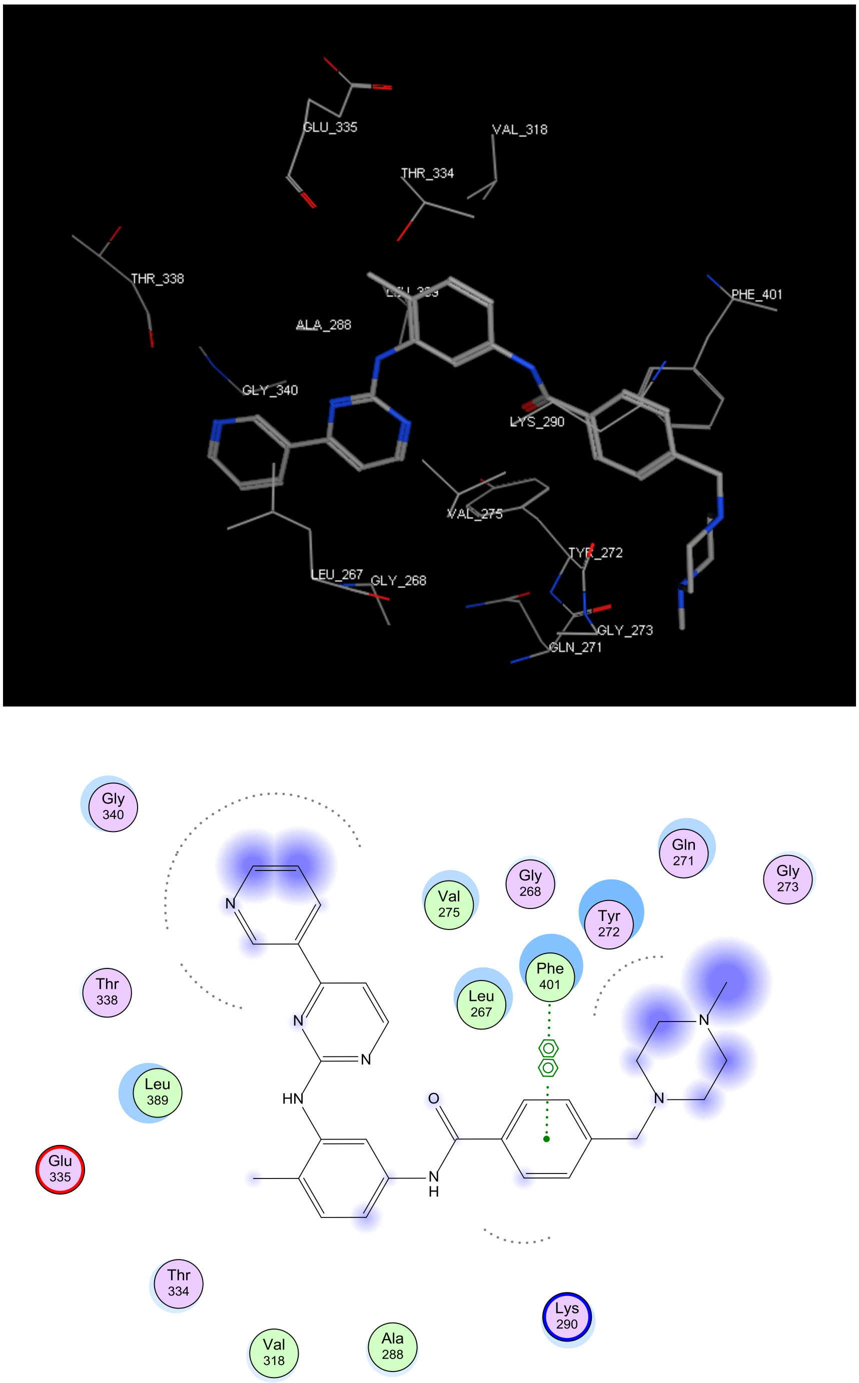

Supplementary Figure S20. Docking of p.(Asp381Asn) mutant ABL structure with Imatinib.

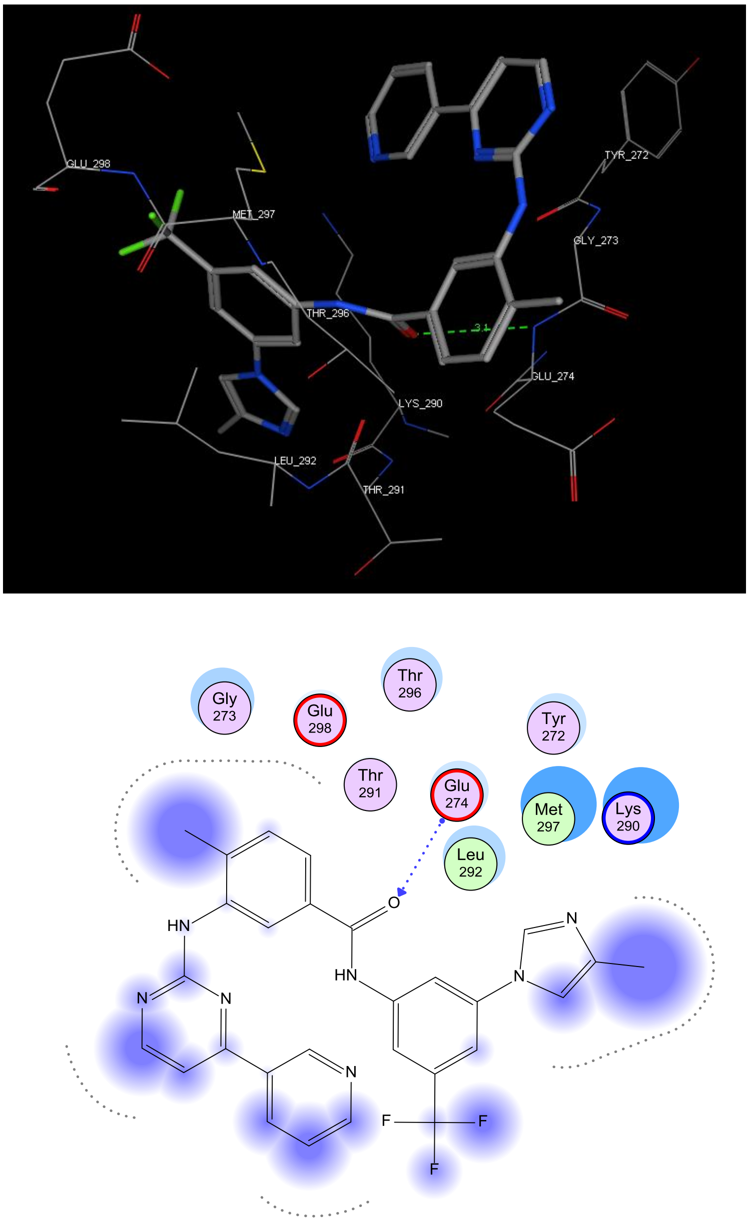

Supplementary Figure S21. Docking of p.(Glu281*) mutant ABL structure with Nilotinib.

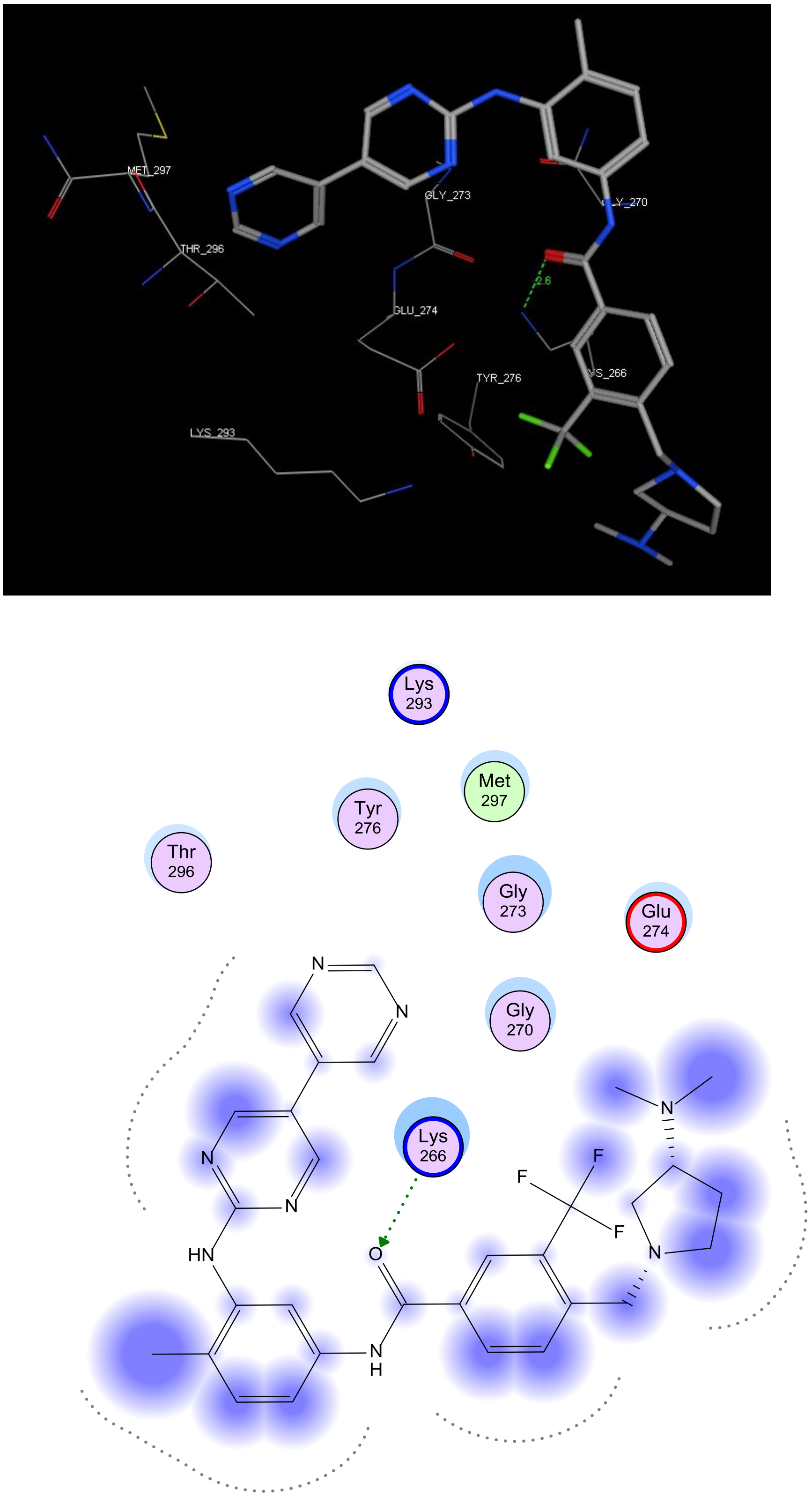

Supplementary Figure S22. Docking of p.(Glu281*) mutant ABL structure with Bafetinib.

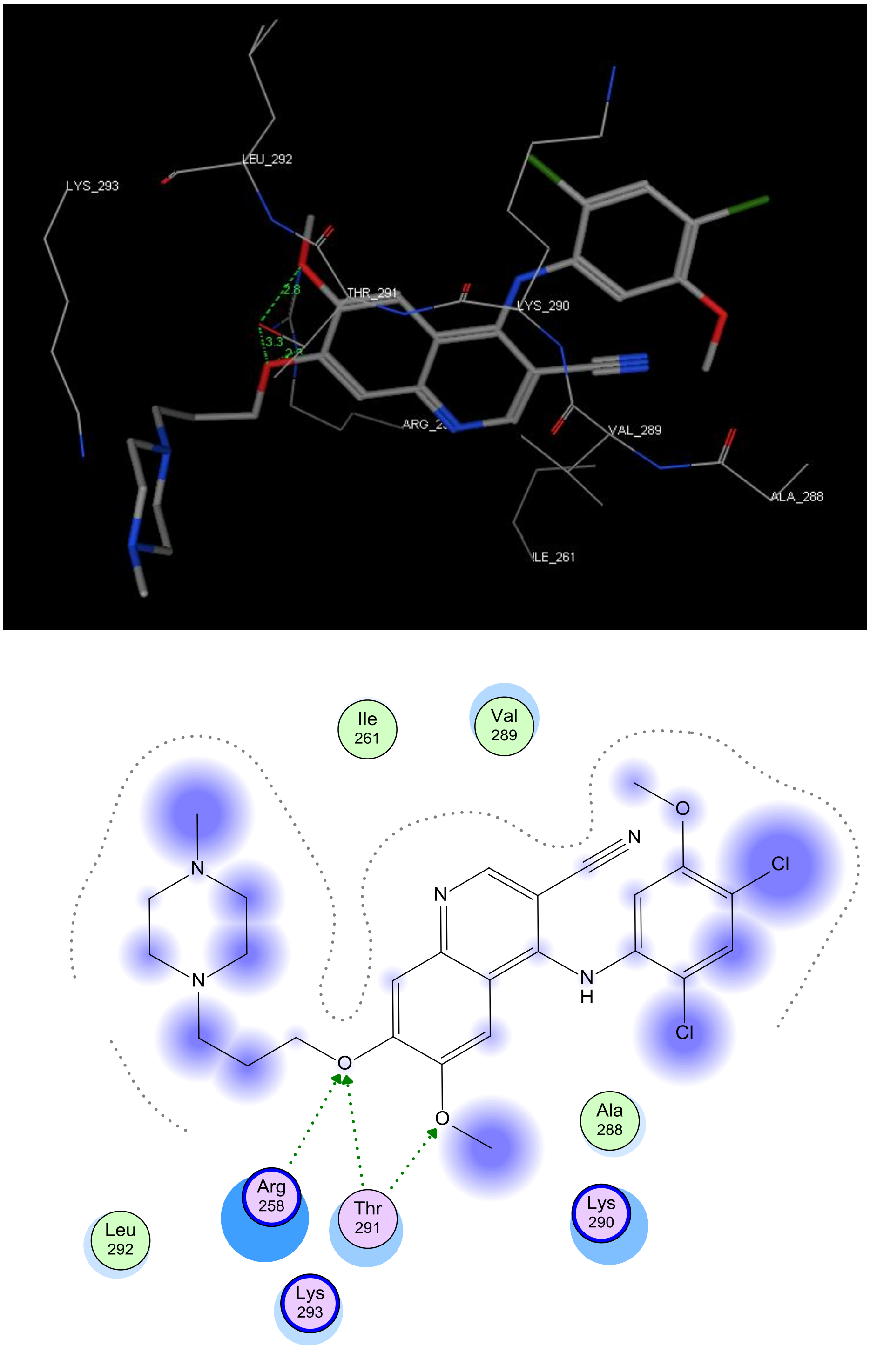

Supplementary Figure S23. Docking of p.(Glu281*) mutant ABL structure with Bosutinib.

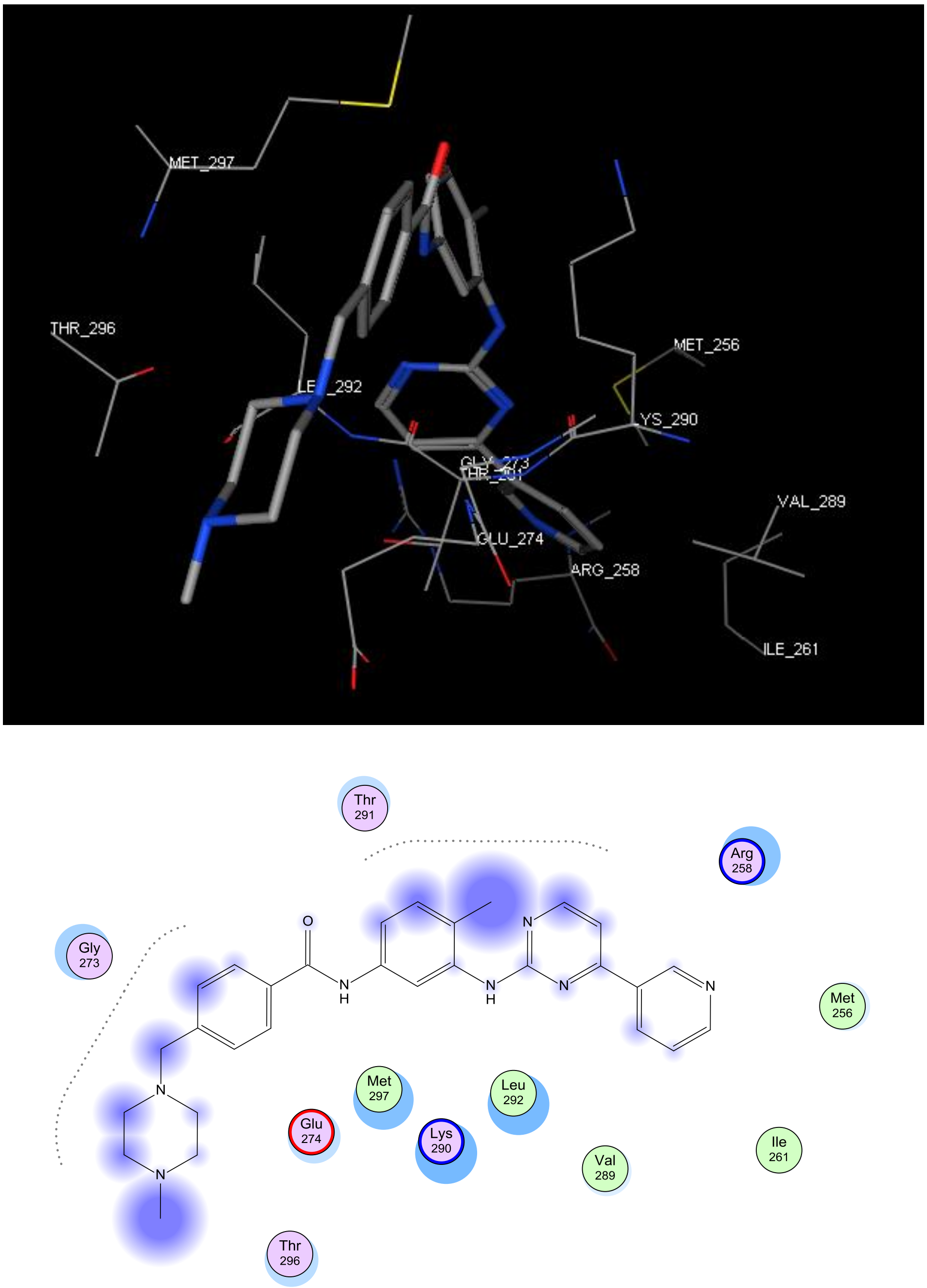

Supplementary Figure S24. Docking of p.(Glu281*) mutant ABL structure with Imatinib.

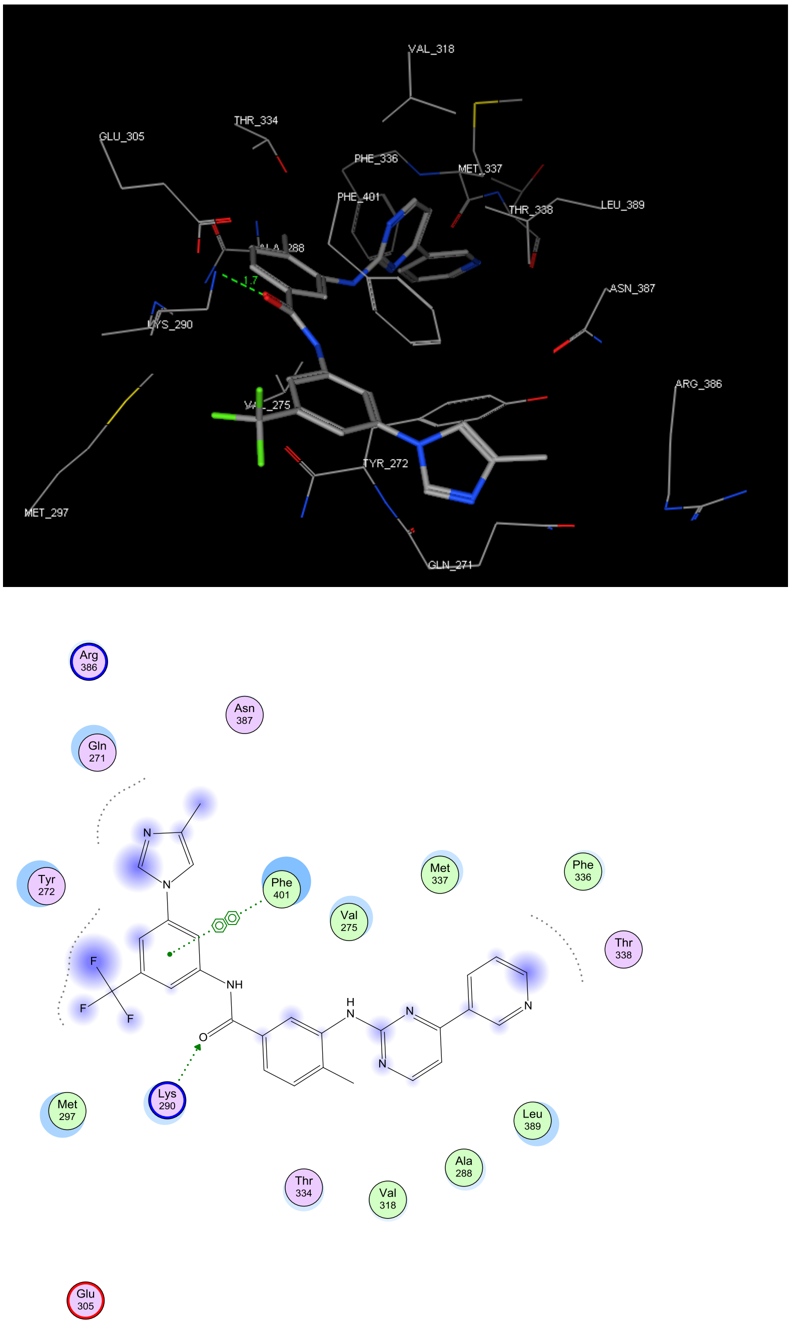

Supplementary Figure S25. Docking of p.(Glu373Asp) mutant ABL structure with Nilotinib.

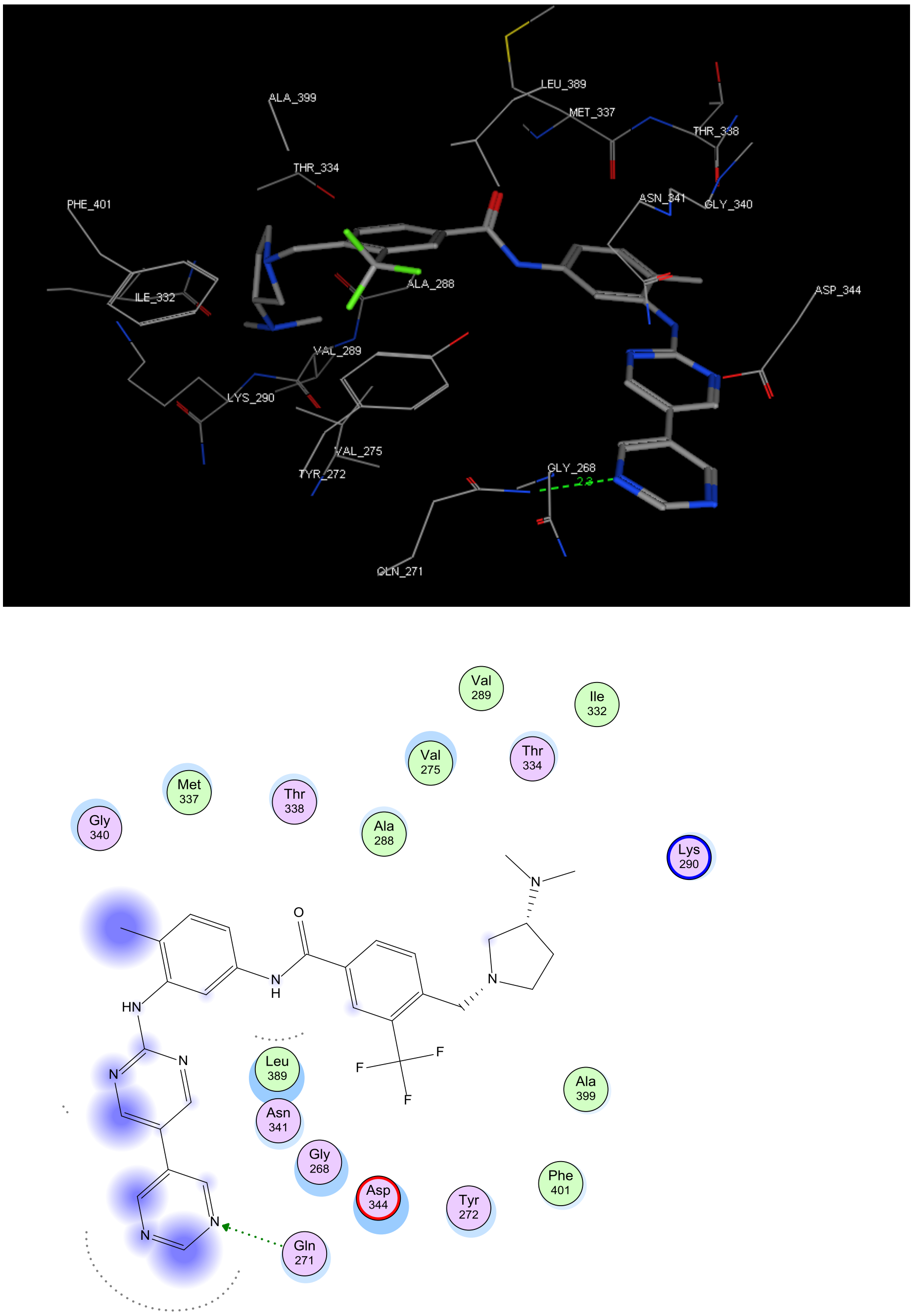

Supplementary Figure S26. Docking of p.(Glu373Asp) mutant ABL structure with Bafetinib.

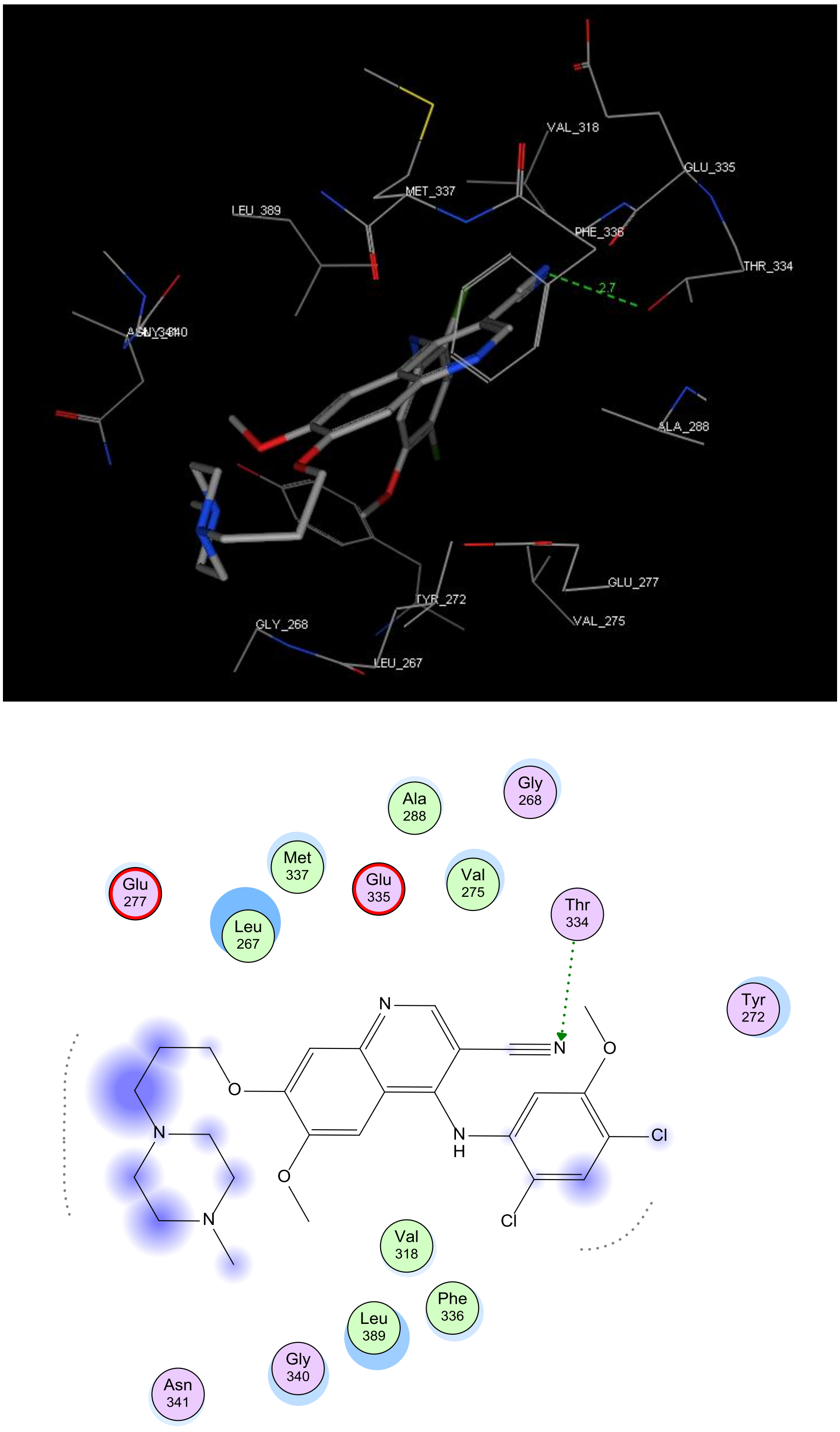

Supplementary Figure S27. Docking of p.(Glu373Asp) mutant ABL structure with Bosutinib.

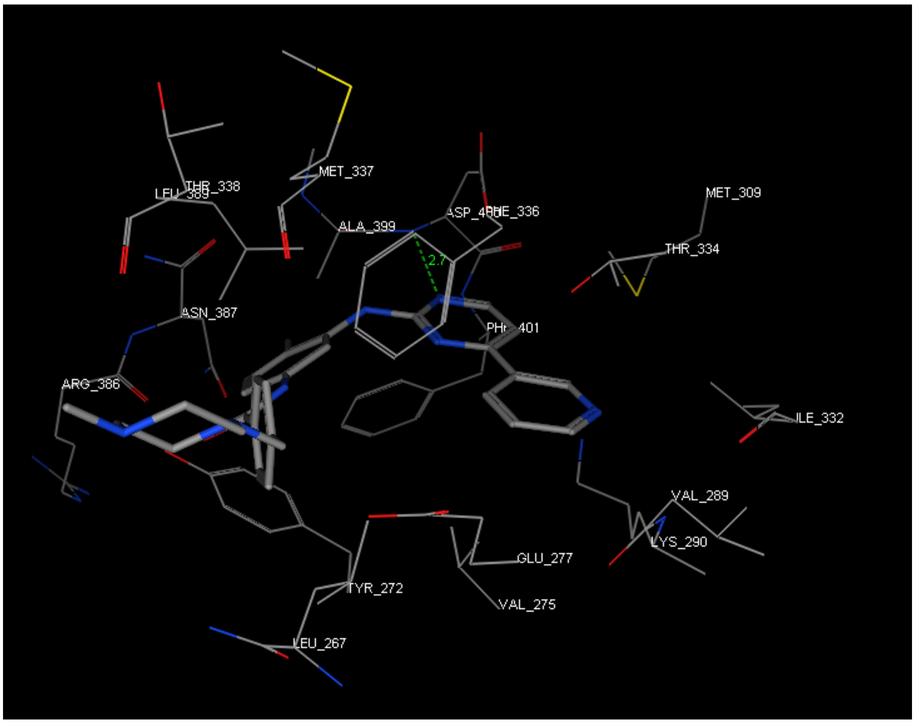

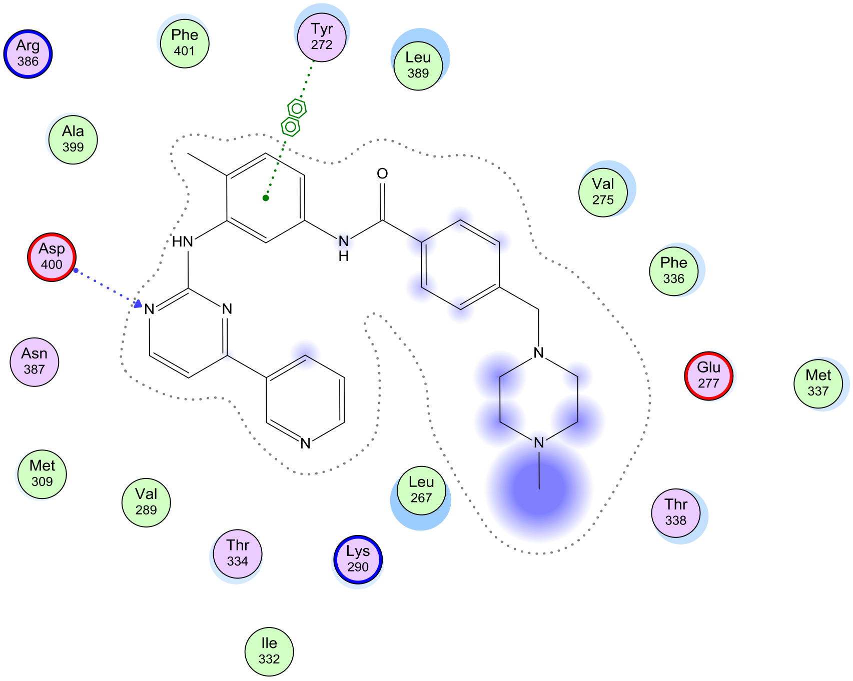

Supplementary Figure S28. Docking of p.(Glu373Asp) mutant ABL structure with Imatinib.

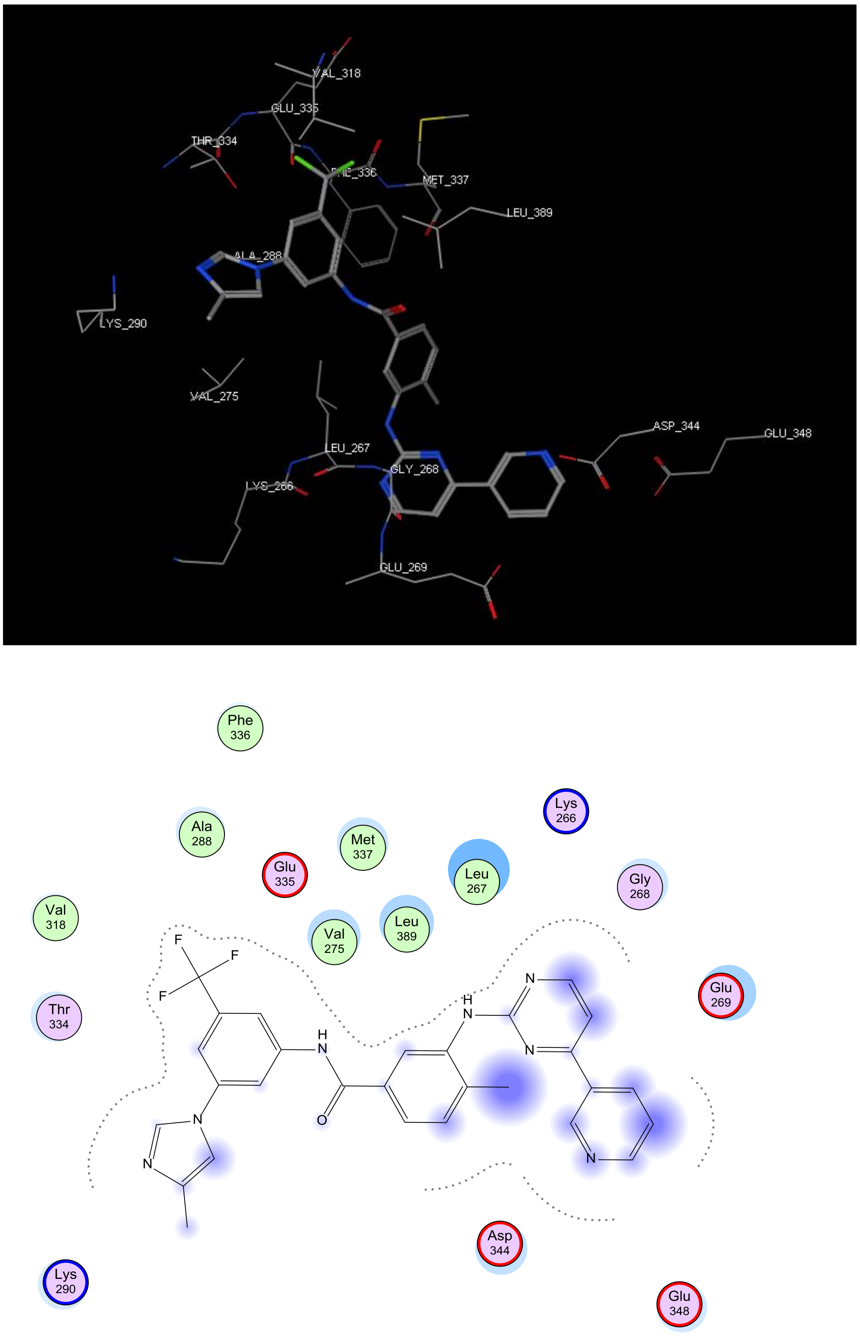

Supplementary Figure S29. Docking of p.(Gly250Glu) mutant ABL structure with Nilotinib.

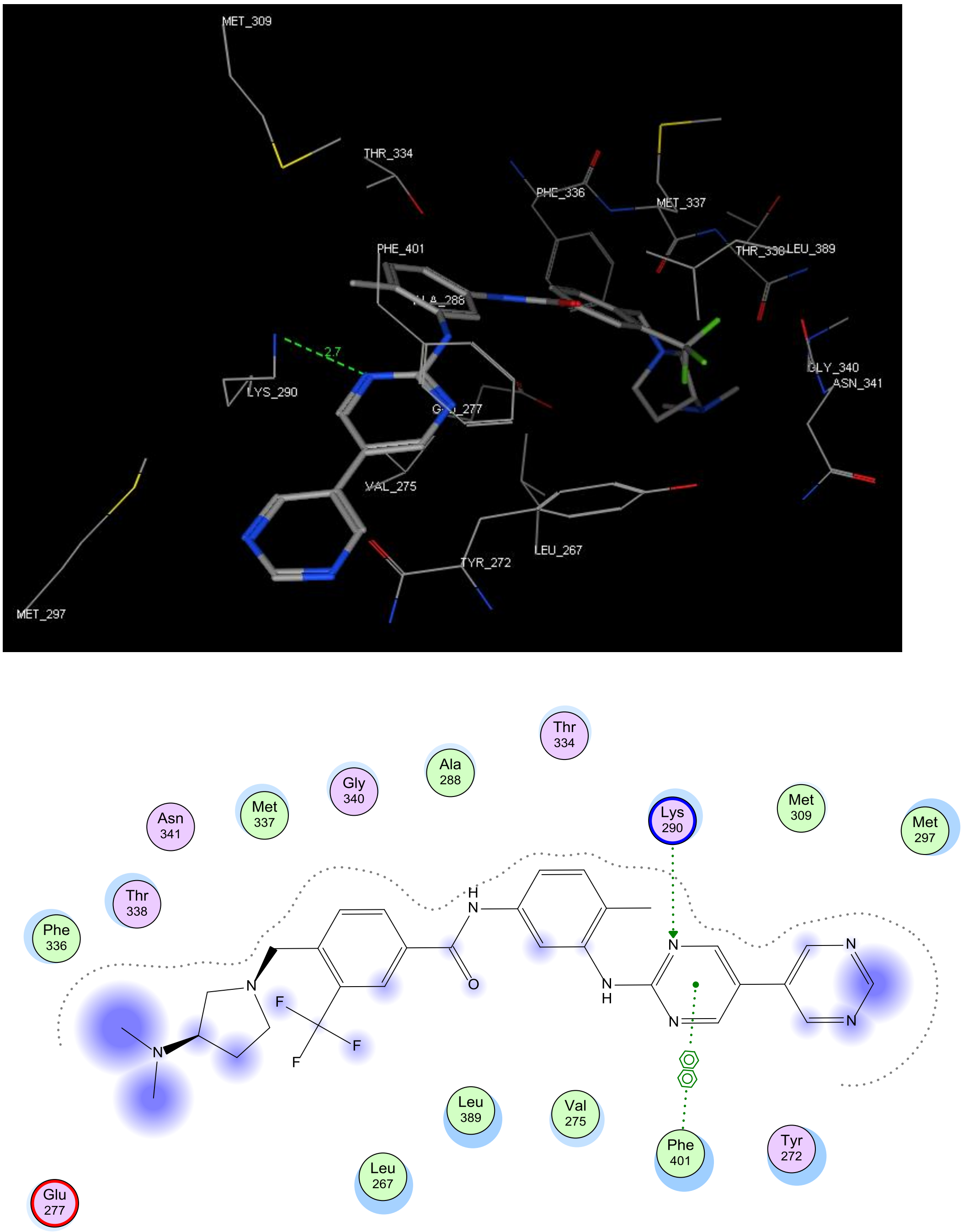

Supplementary Figure S30. Docking of p.(Gly250Glu) mutant ABL structure with Bafetinib.

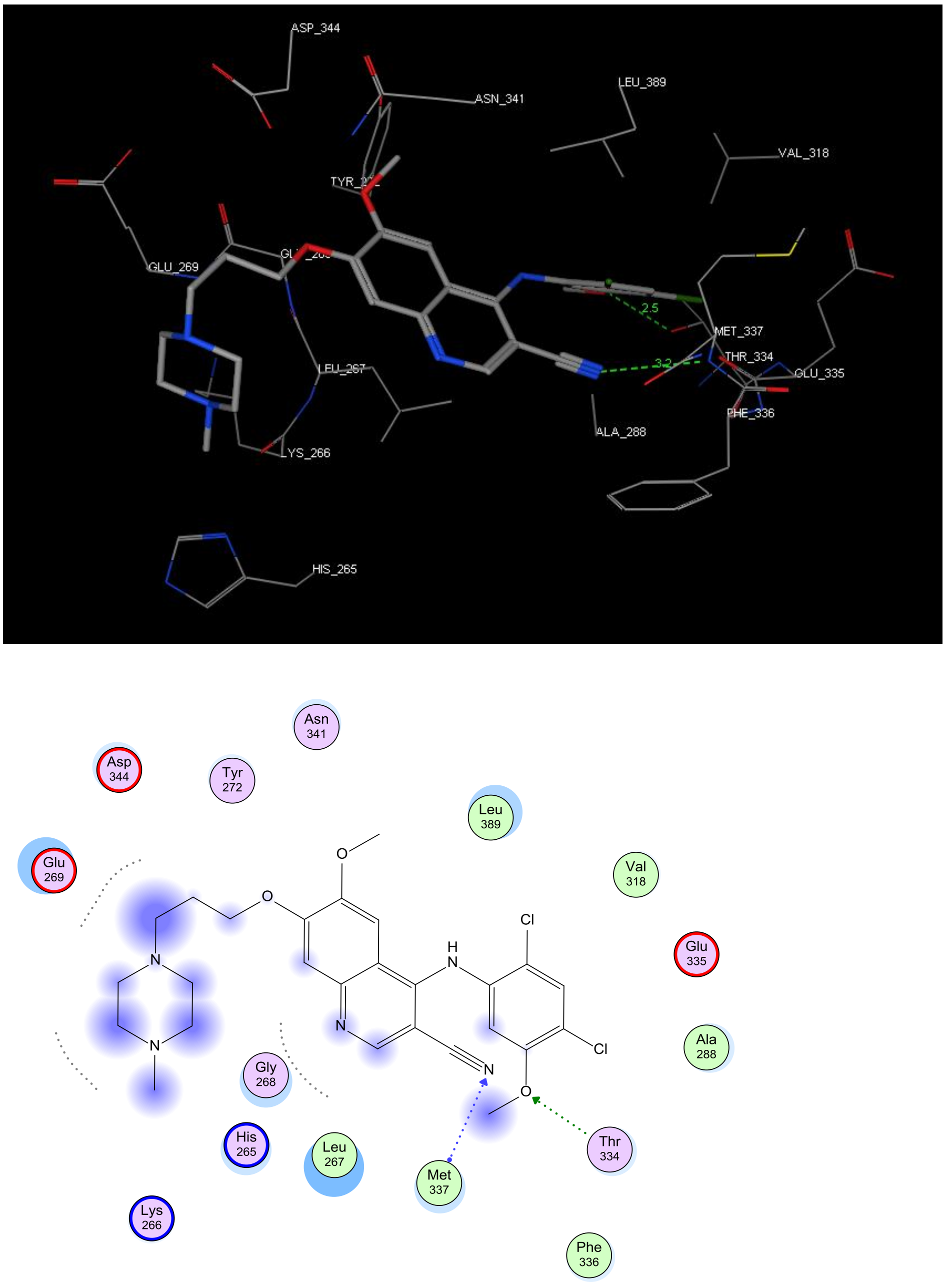

Supplementary Figure S31. Docking of p.(Gly250Glu) mutant ABL structure with Bosutinib.

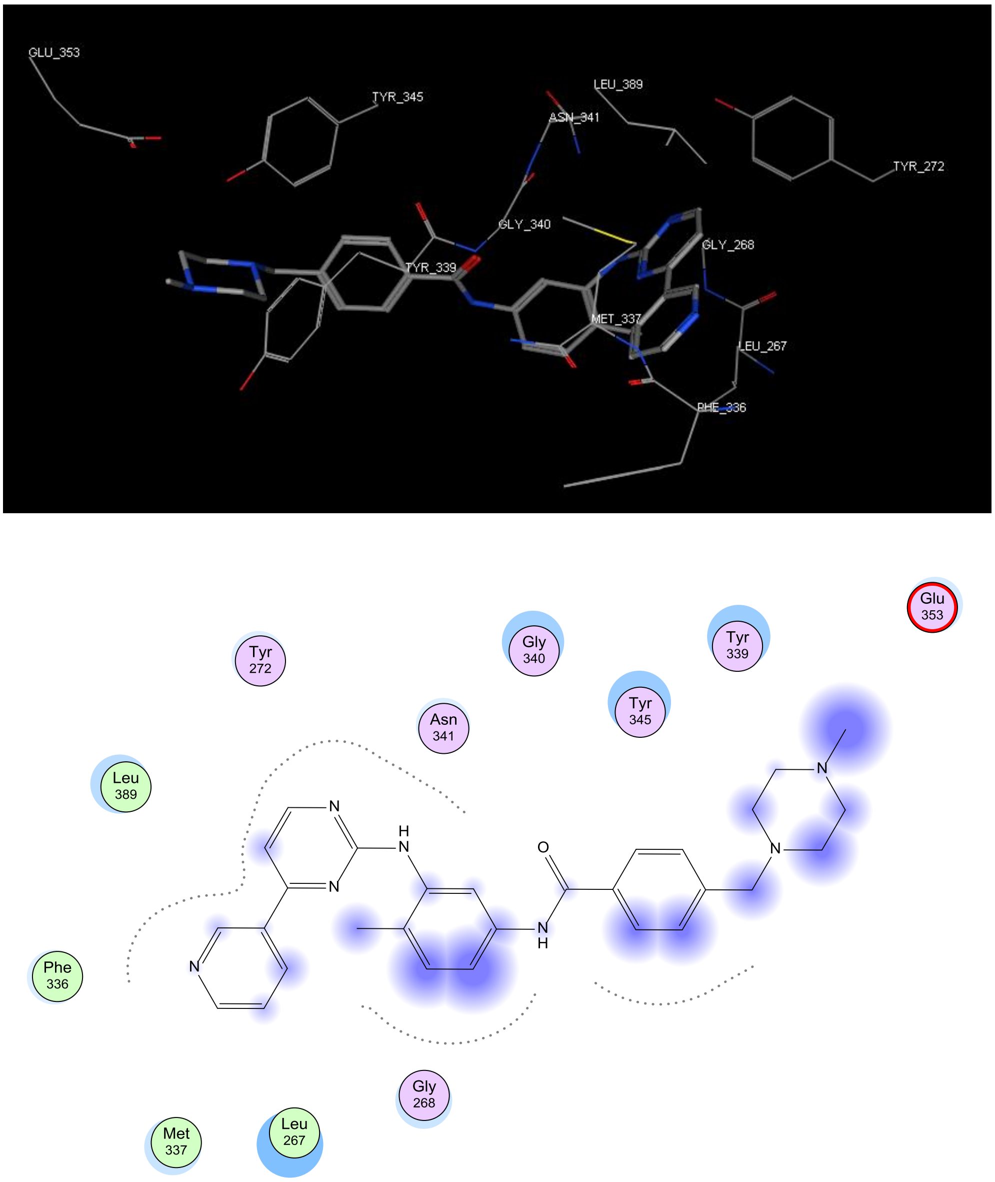

Supplementary Figure S32. Docking of p.(Gly250Glu) mutant ABL structure with Imatinib.

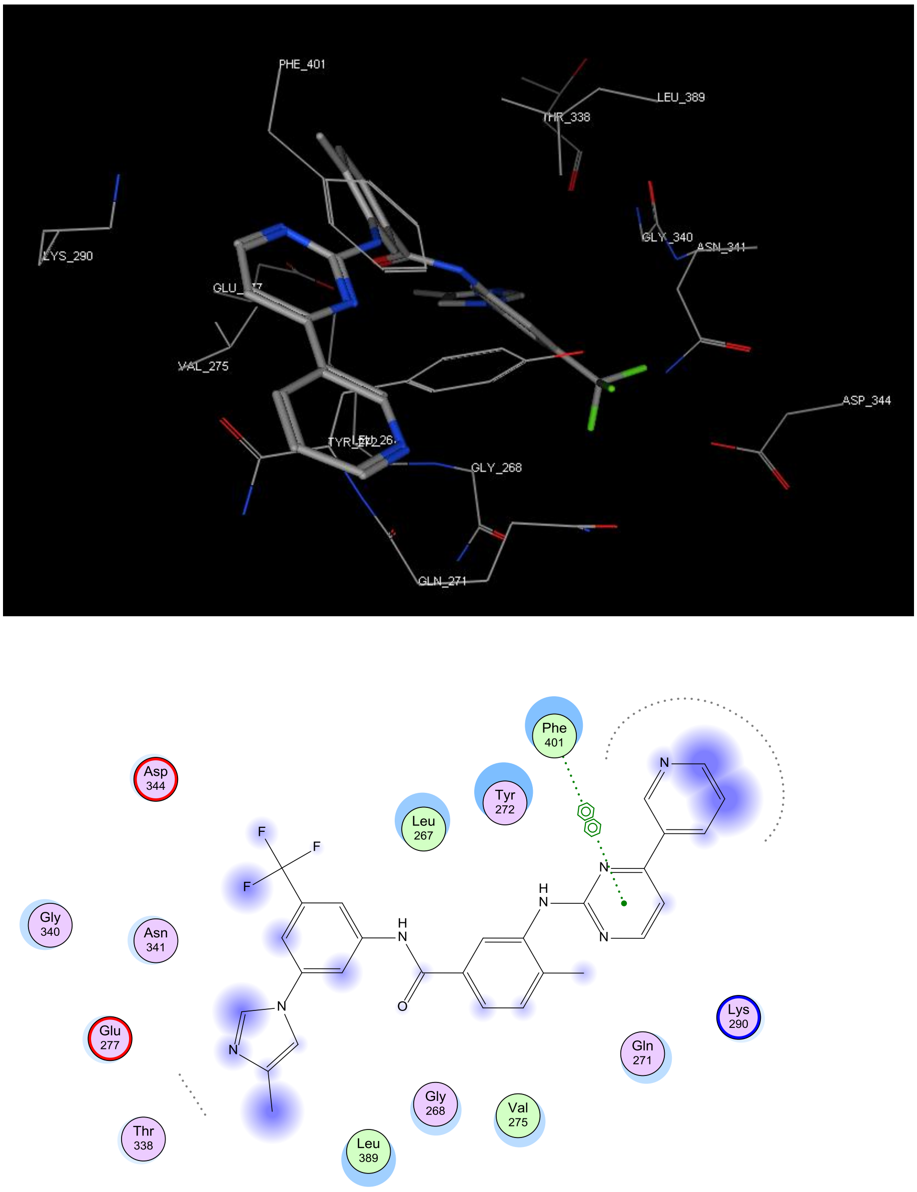

Supplementary Figure S33. Docking of p.(Leu301Ile) mutant ABL structure with Nilotinib.

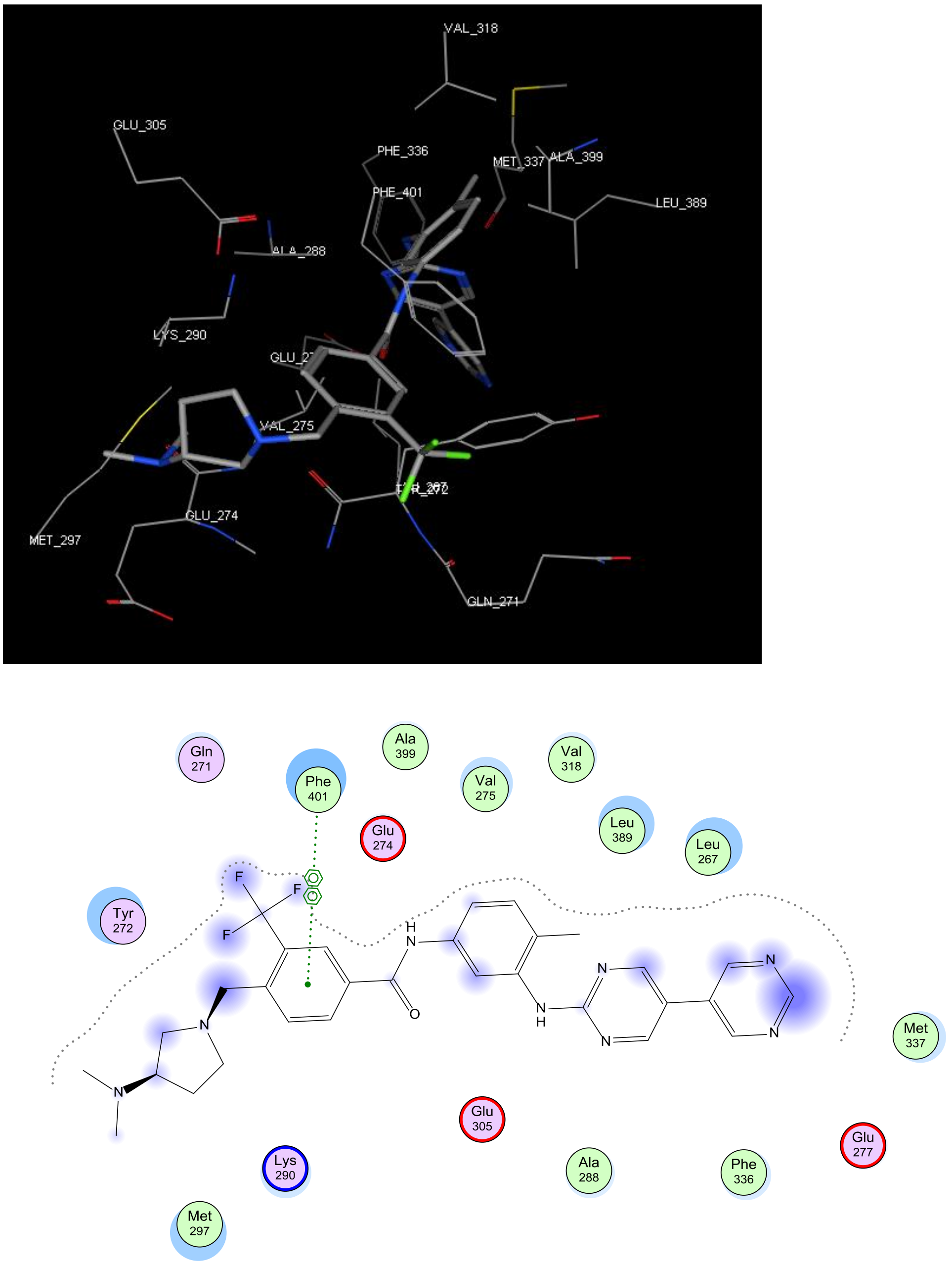

Supplementary Figure S34. Docking of p.(Leu301Ile) mutant ABL structure with Bafetinib.

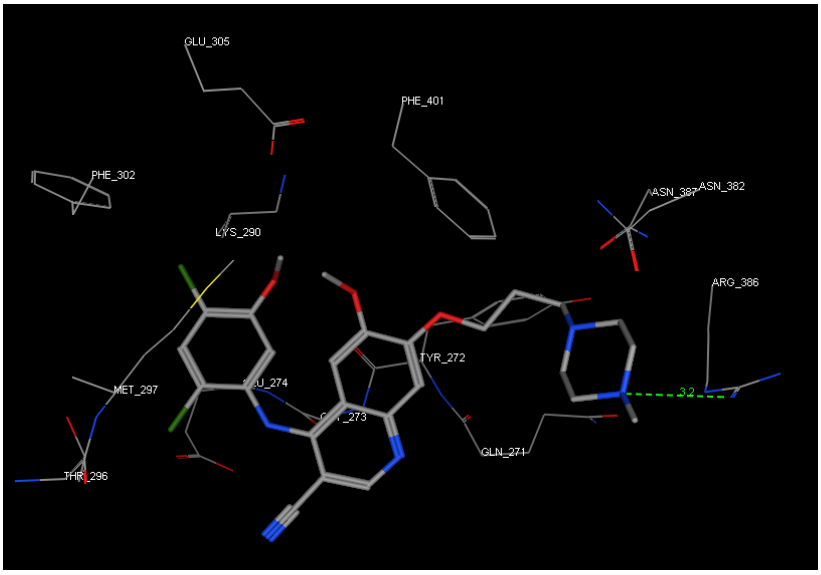

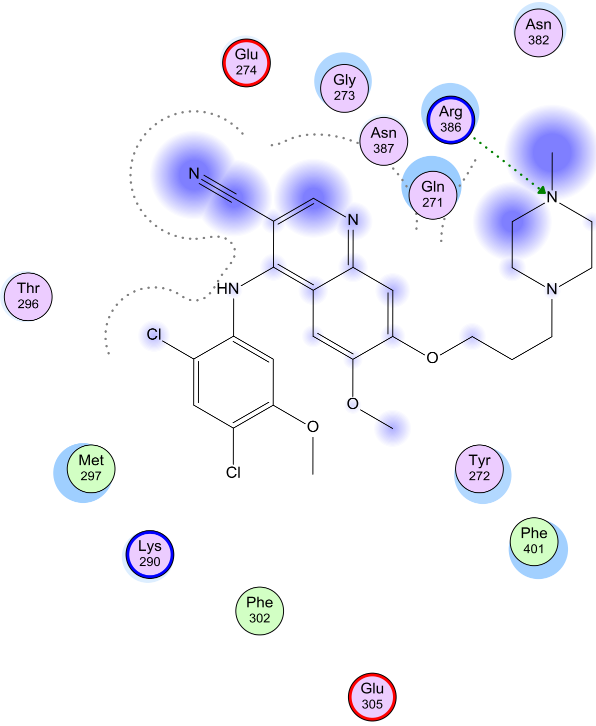

Supplementary Figure S35. Docking of p.(Leu301Ile) mutant ABL structure with Bosutinib.

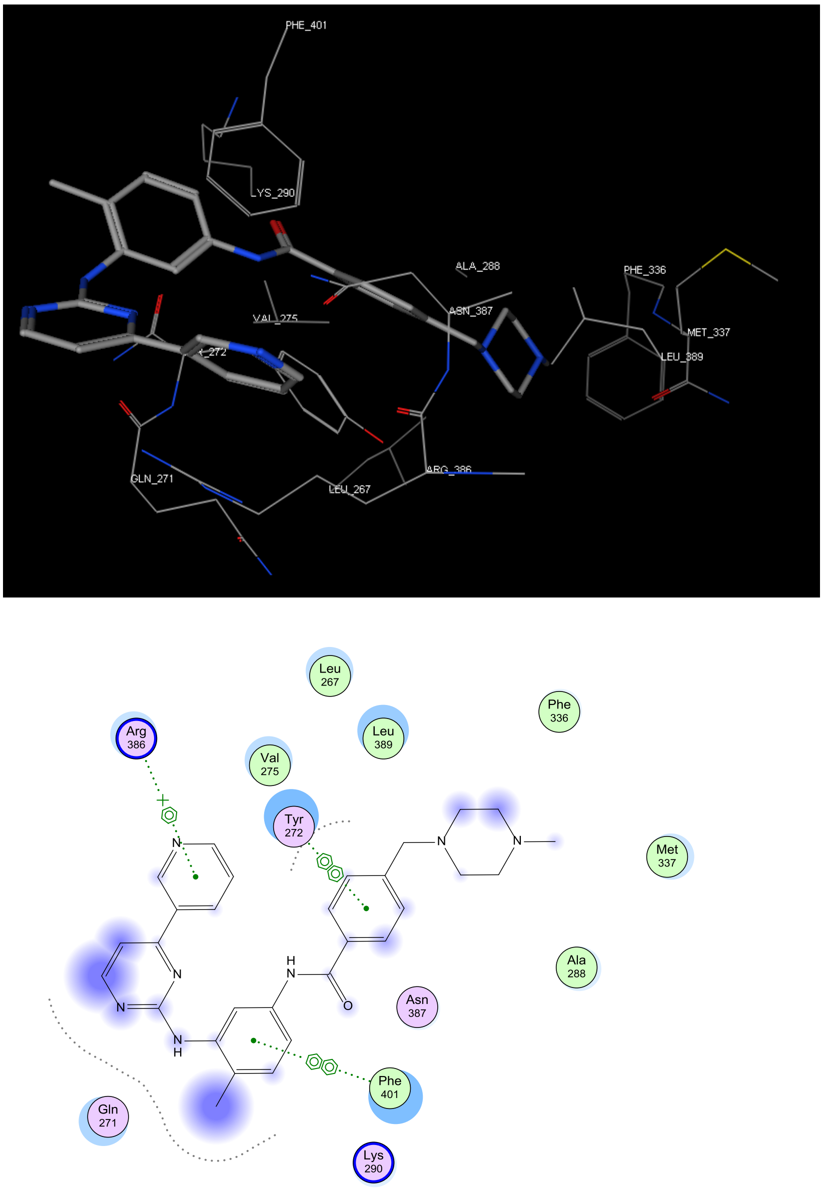

Supplementary Figure S36. Docking of p.(Leu301Ile) mutant ABL structure with Imatinib.

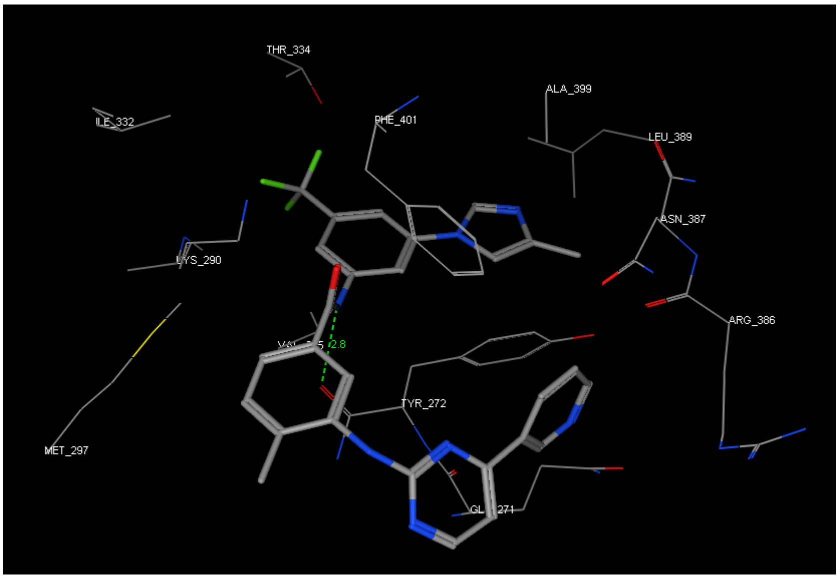

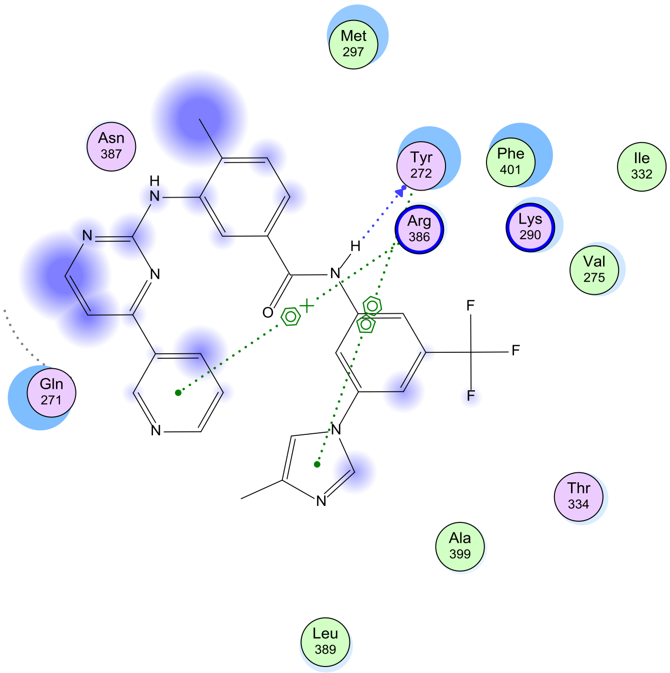

Supplementary Figure S37. Docking of p.(Met351Thr) mutant ABL structure with Nilotinib.

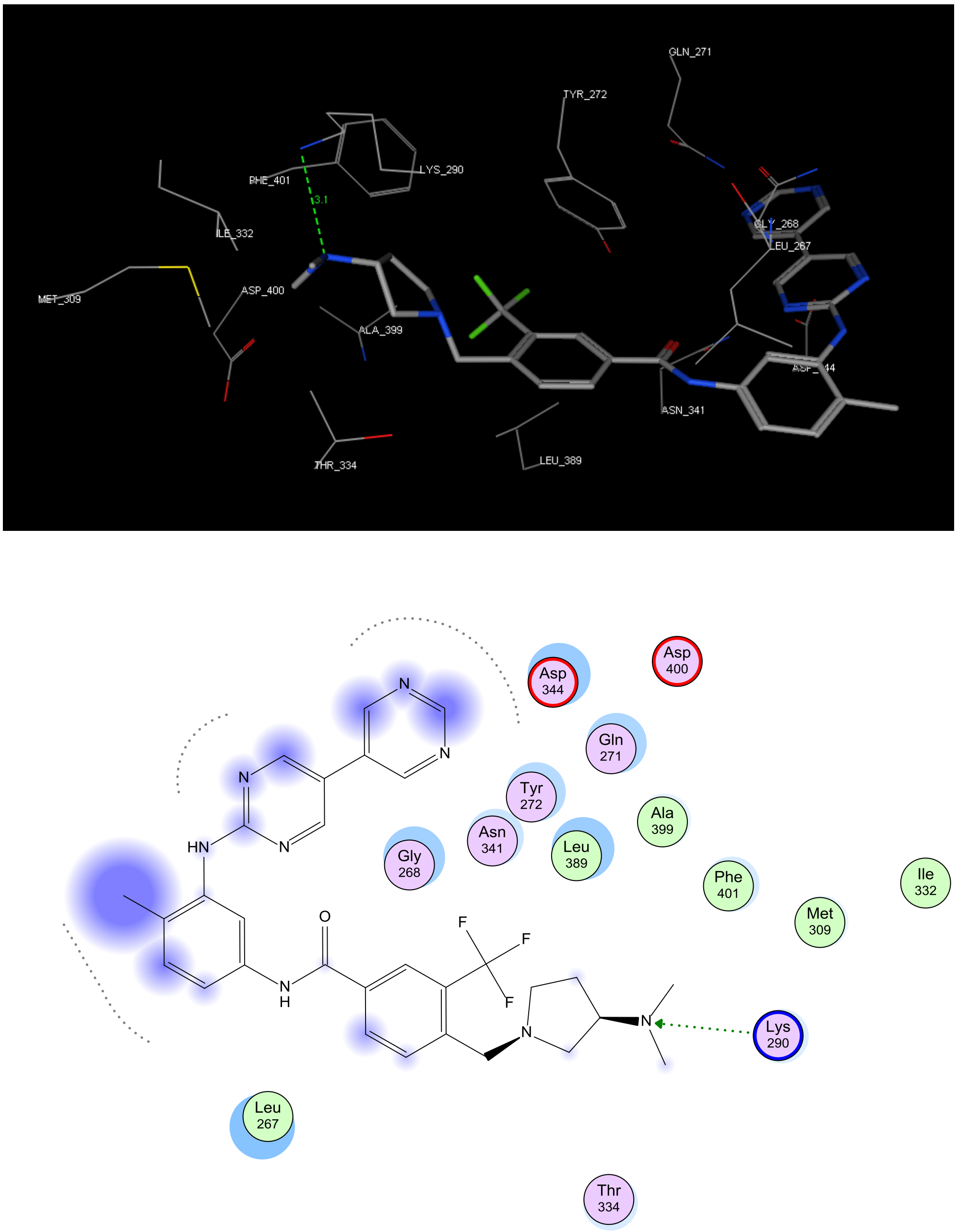

Supplementary Figure S38. Docking of p.(Met351Thr) mutant ABL structure with Bafetinib.

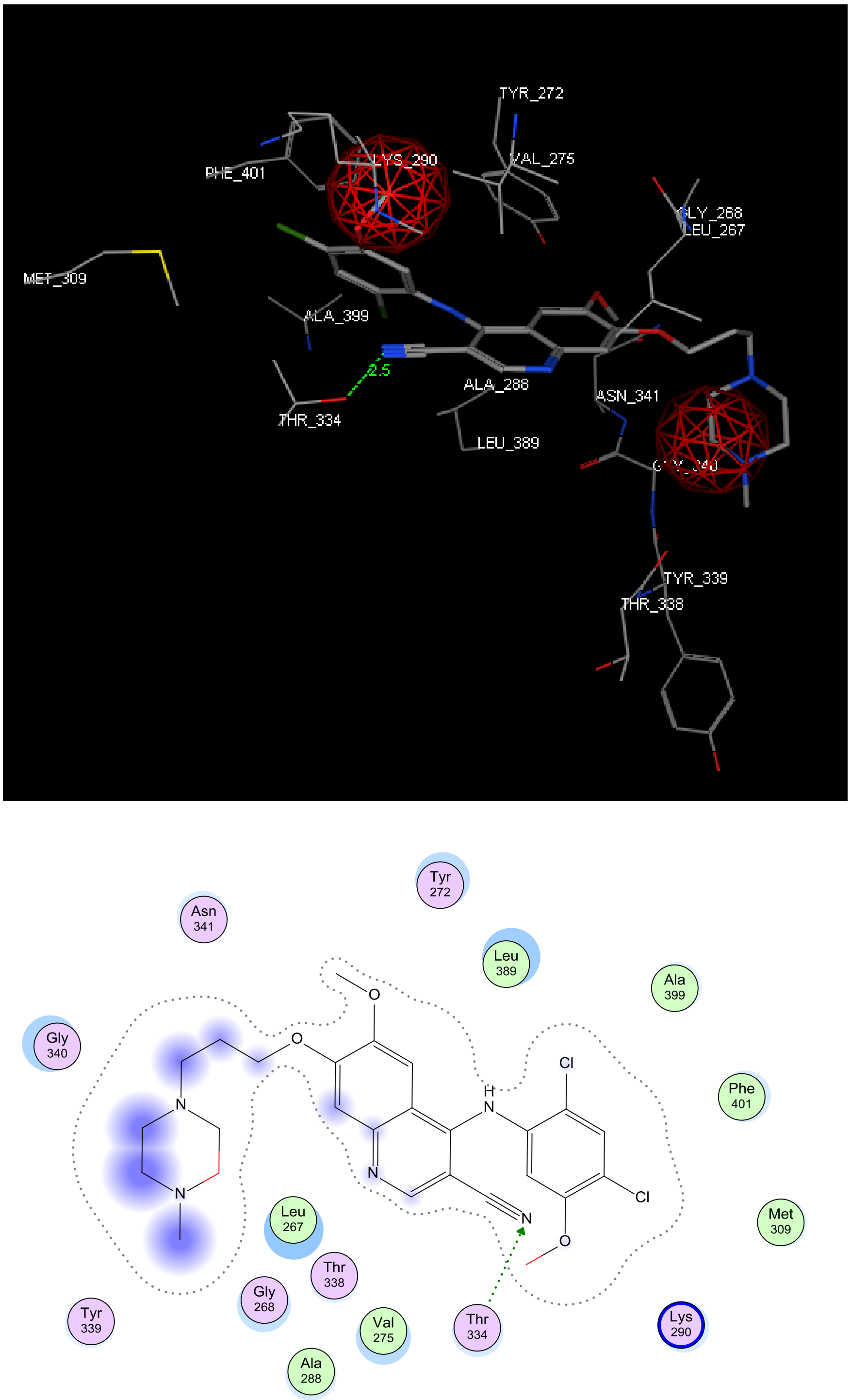

Supplementary Figure S39. Docking of p.(Met351Thr) mutant ABL structure with Bosutinib.

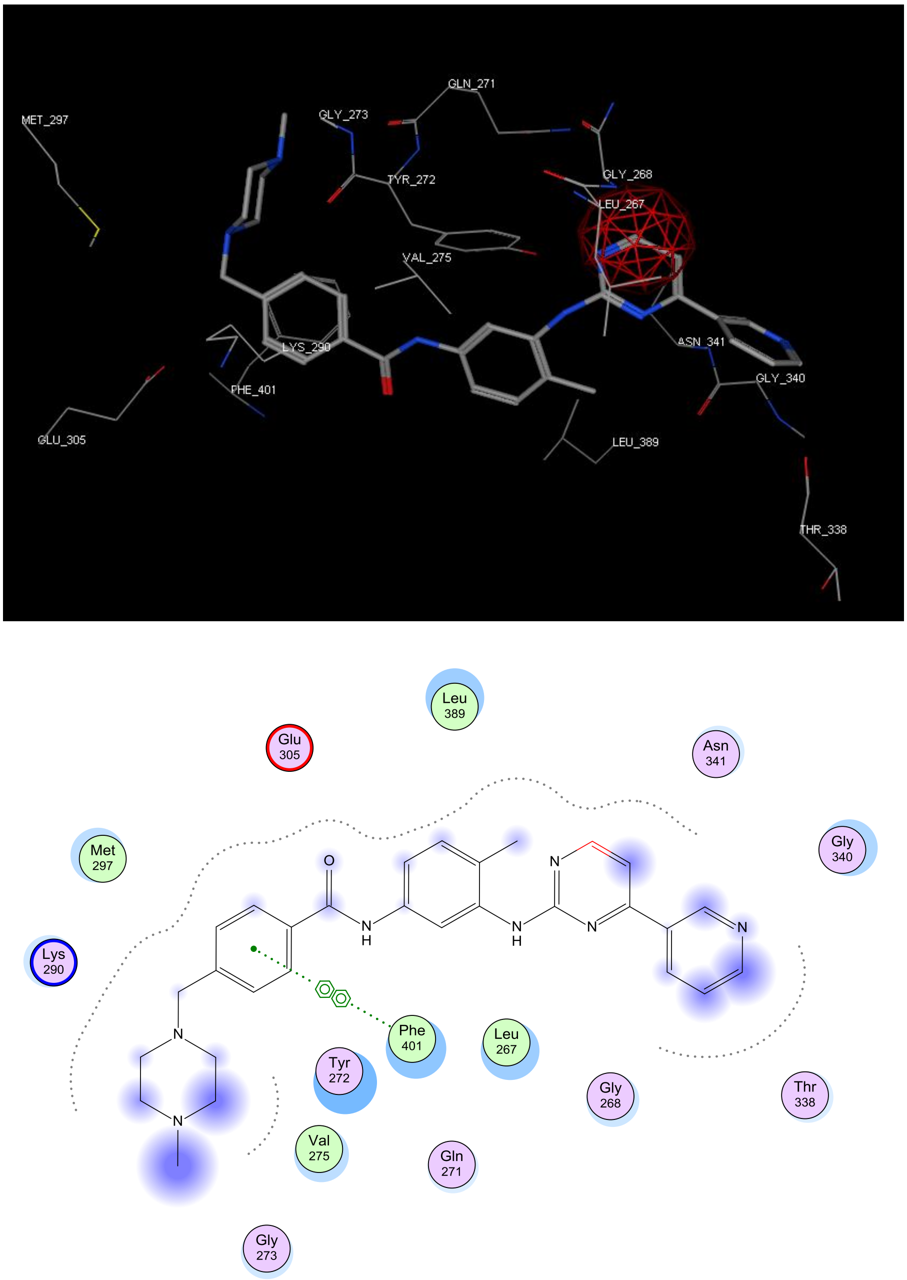

Supplementary Figure S40. Docking of p.(Met351Thr) mutant ABL structure with Imatinib.

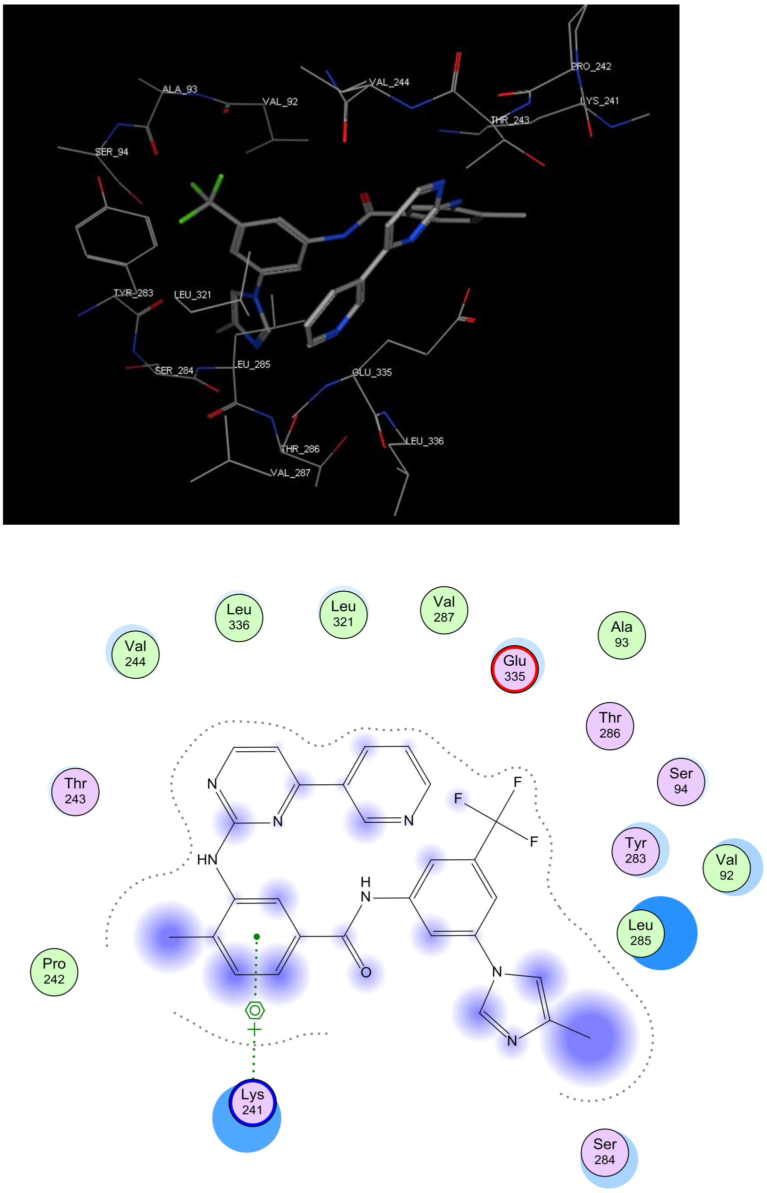

Supplementary Figure S41. Docking of p.(Phe317Leu) mutant ABL structure with Nilotinib.

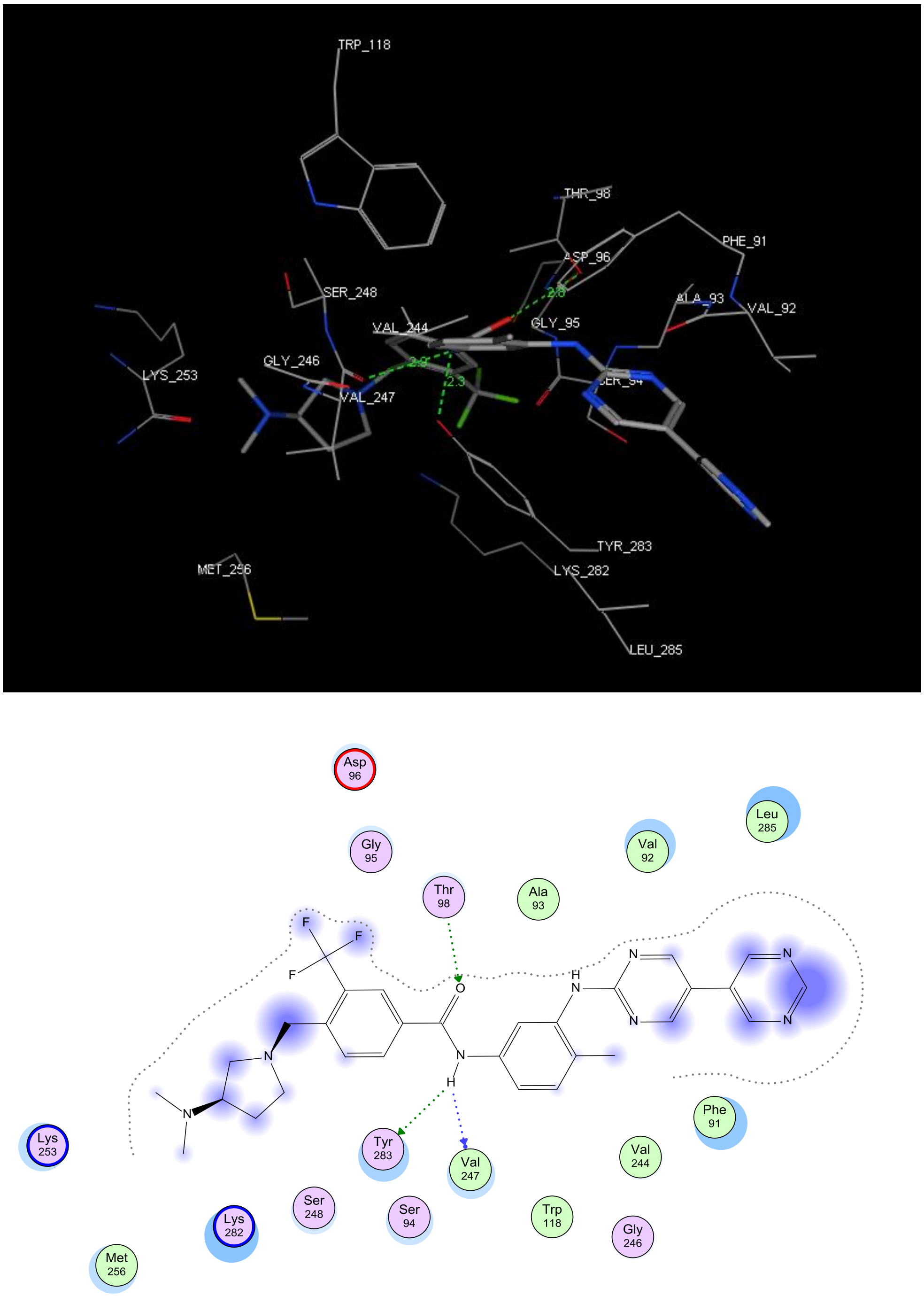

Supplementary Figure S42. Docking of p.(Phe317Leu) mutant ABL structure with Bafetinib.

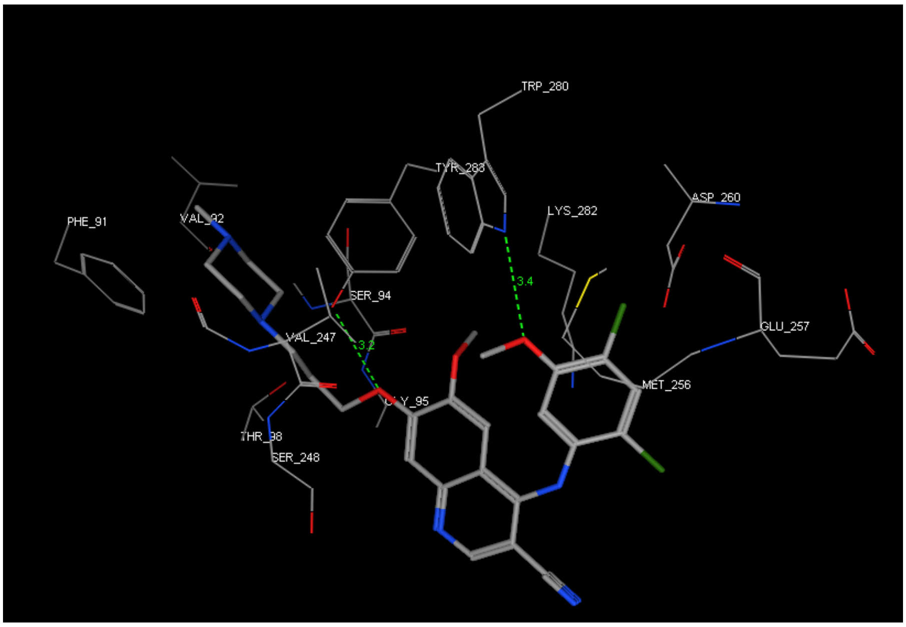

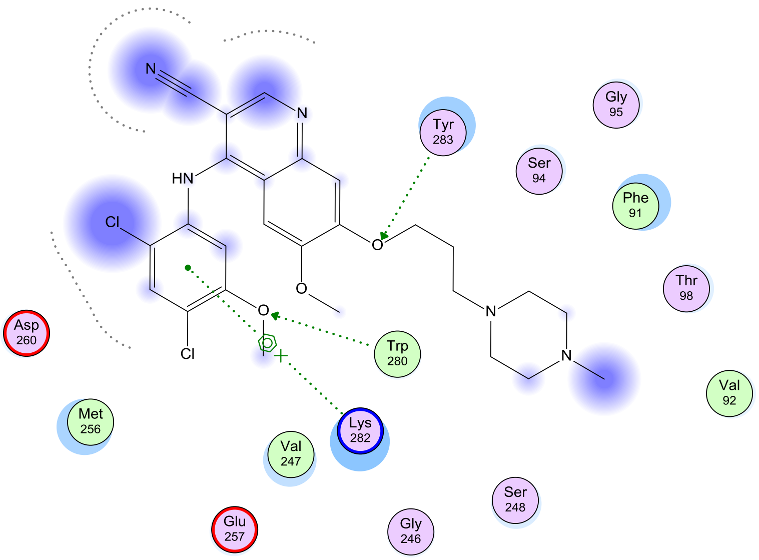

Supplementary Figure S43. Docking of p.(Phe317Leu) mutant ABL structure with Bosutinib.

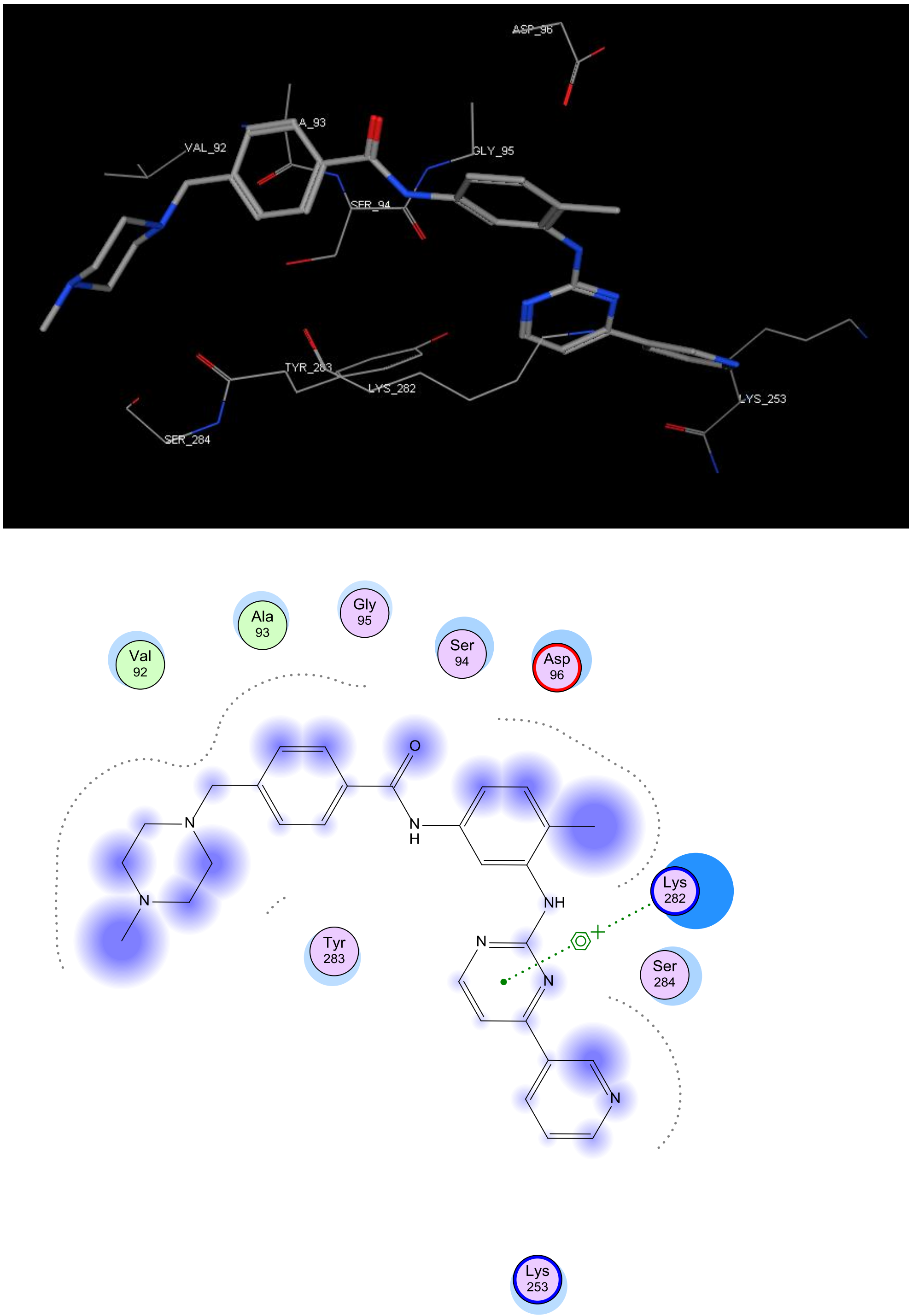

Supplementary Figure S44. Docking of p.(Phe317Leu) mutant ABL structure with Imatinib.

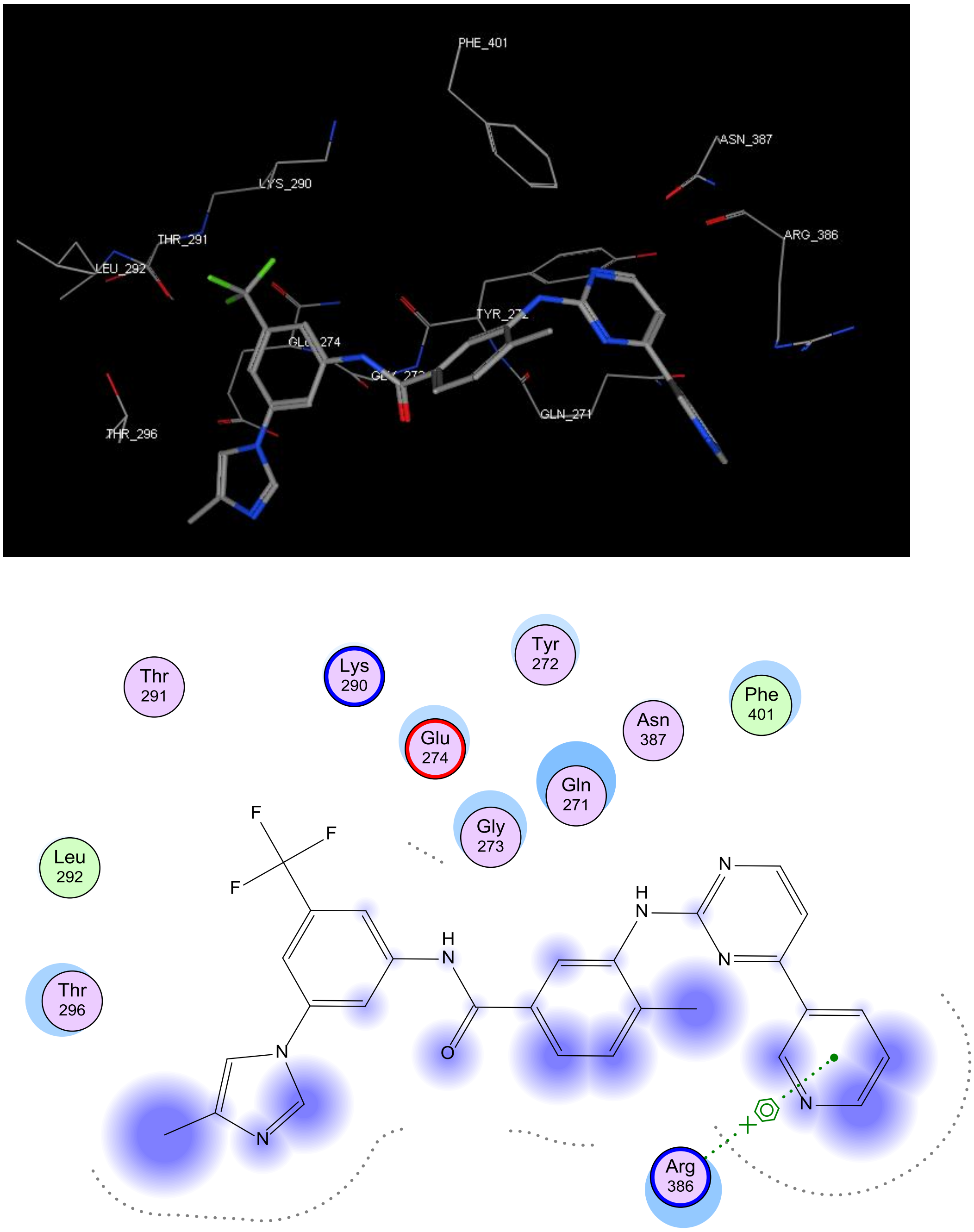

Supplementary Figure S45. Docking of p.(Thr315Ile) mutant ABL structure with Nilotinib.

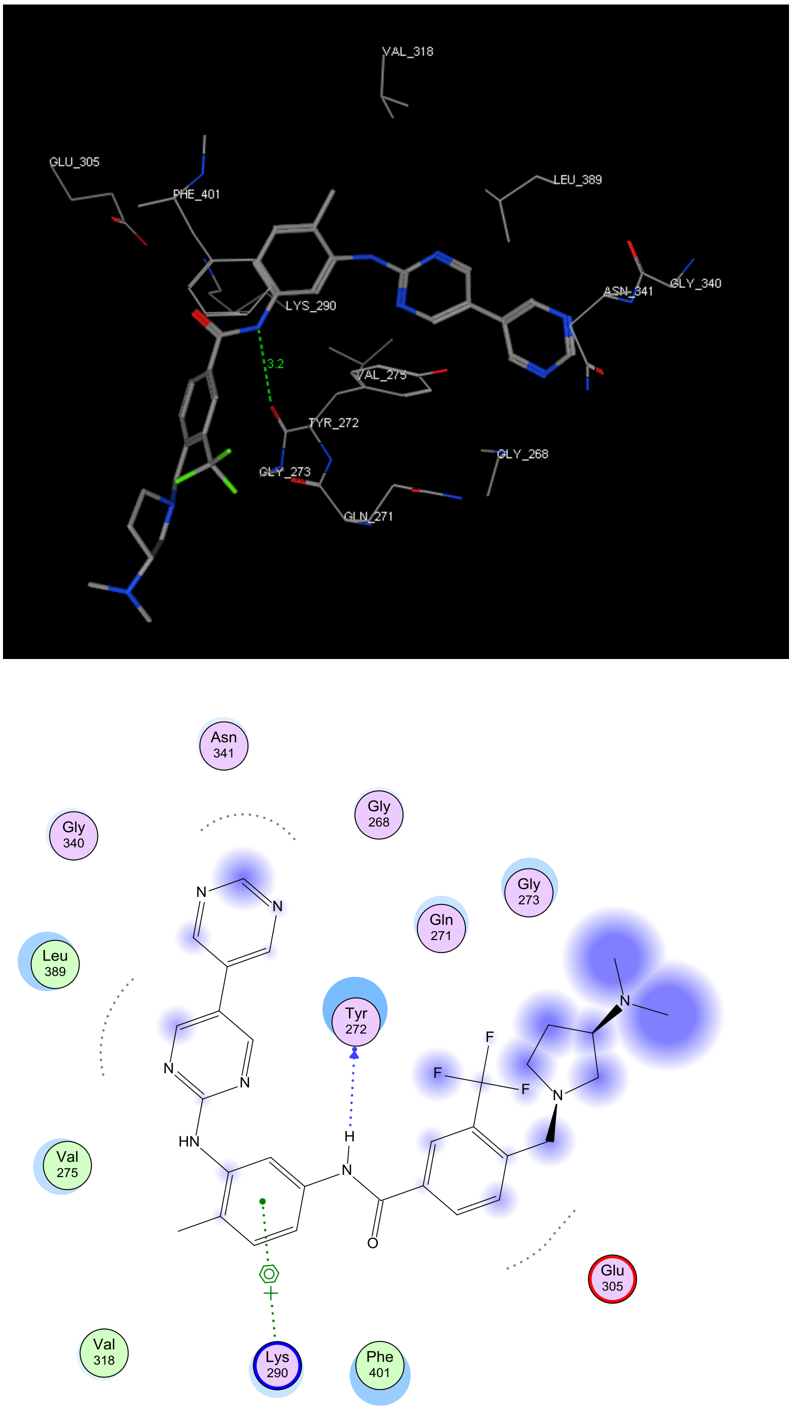

Supplementary Figure S46. Docking of p.(Thr315Ile) mutant ABL structure with Bafetinib.

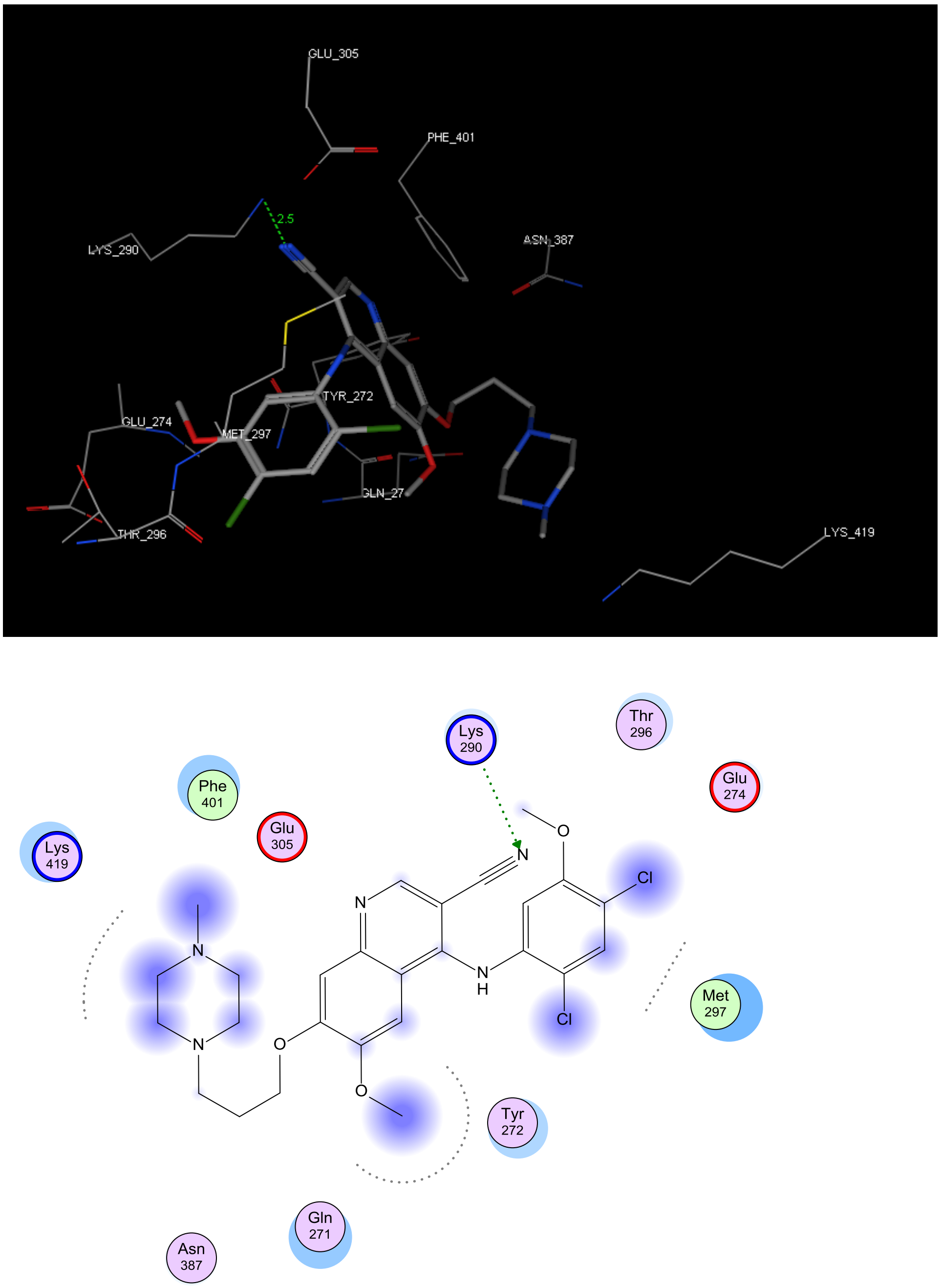

Supplementary Figure S47. Docking of p.(Thr315Ile) mutant ABL structure with Bosutinib.

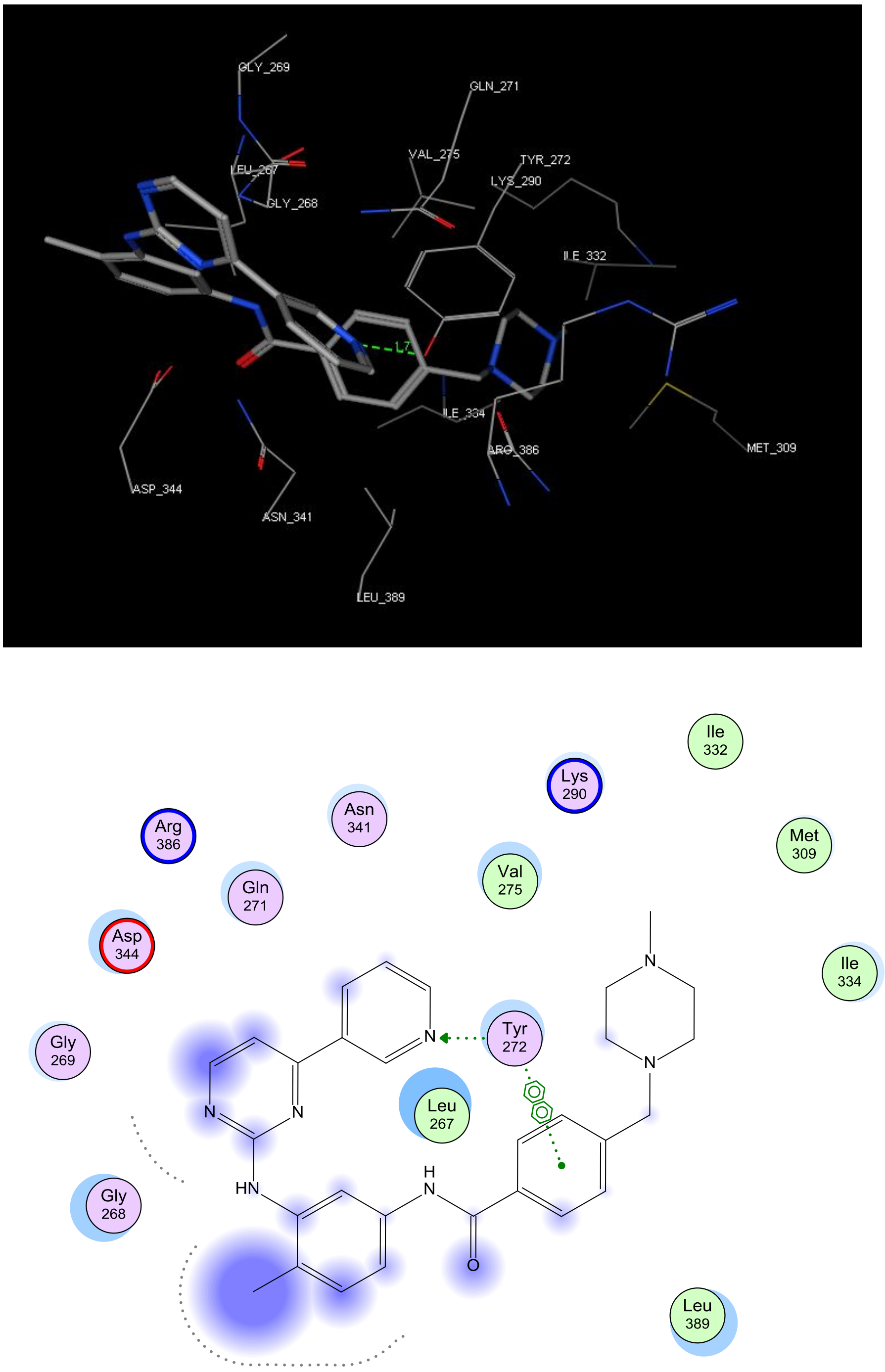

Supplementary Figure S48. Docking of p.(Thr315Ile) mutant ABL structure with Imatinib.

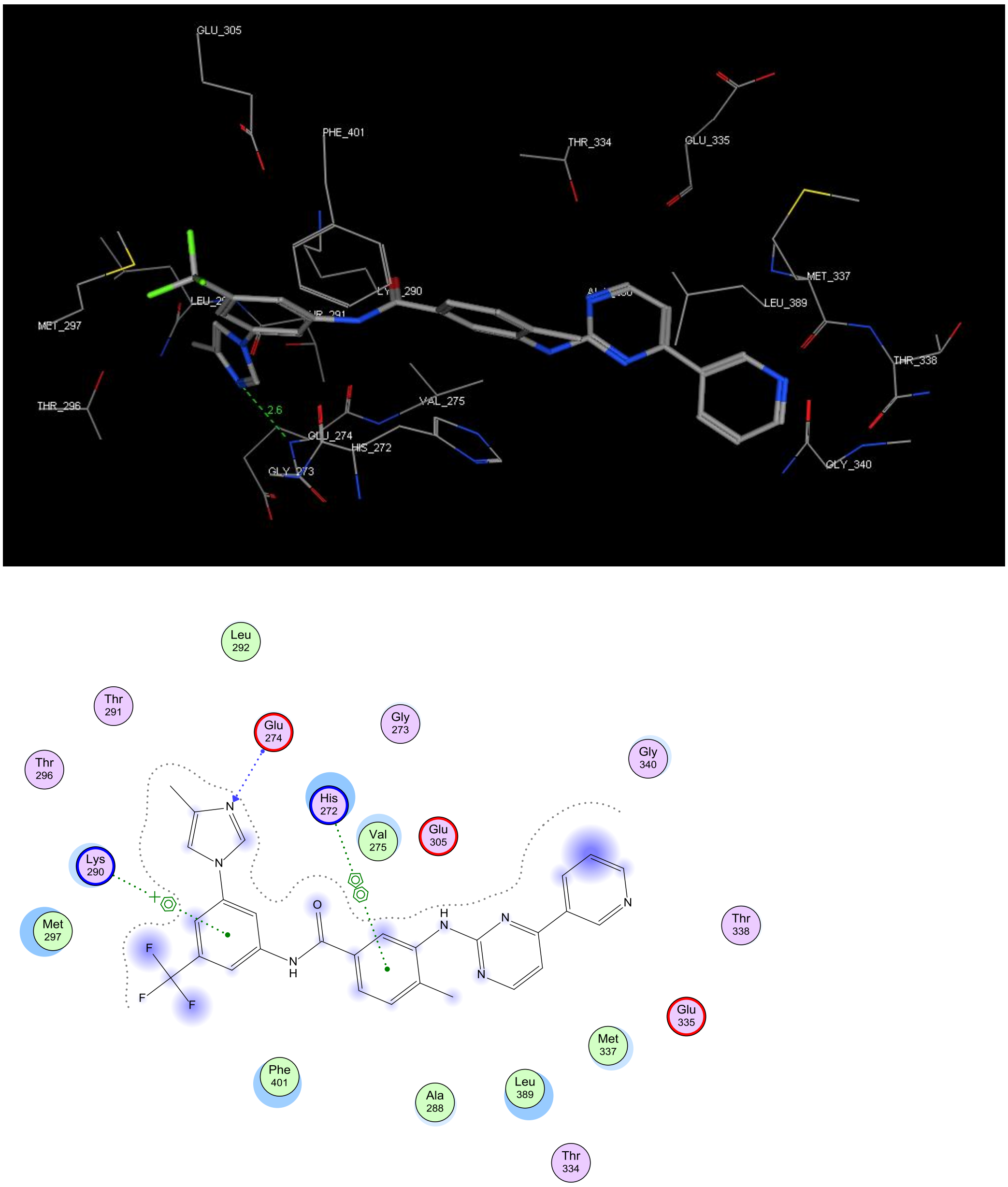

Supplementary Figure S49. Docking of p.(Tyr253His) mutant ABL structure with Nilotinib.

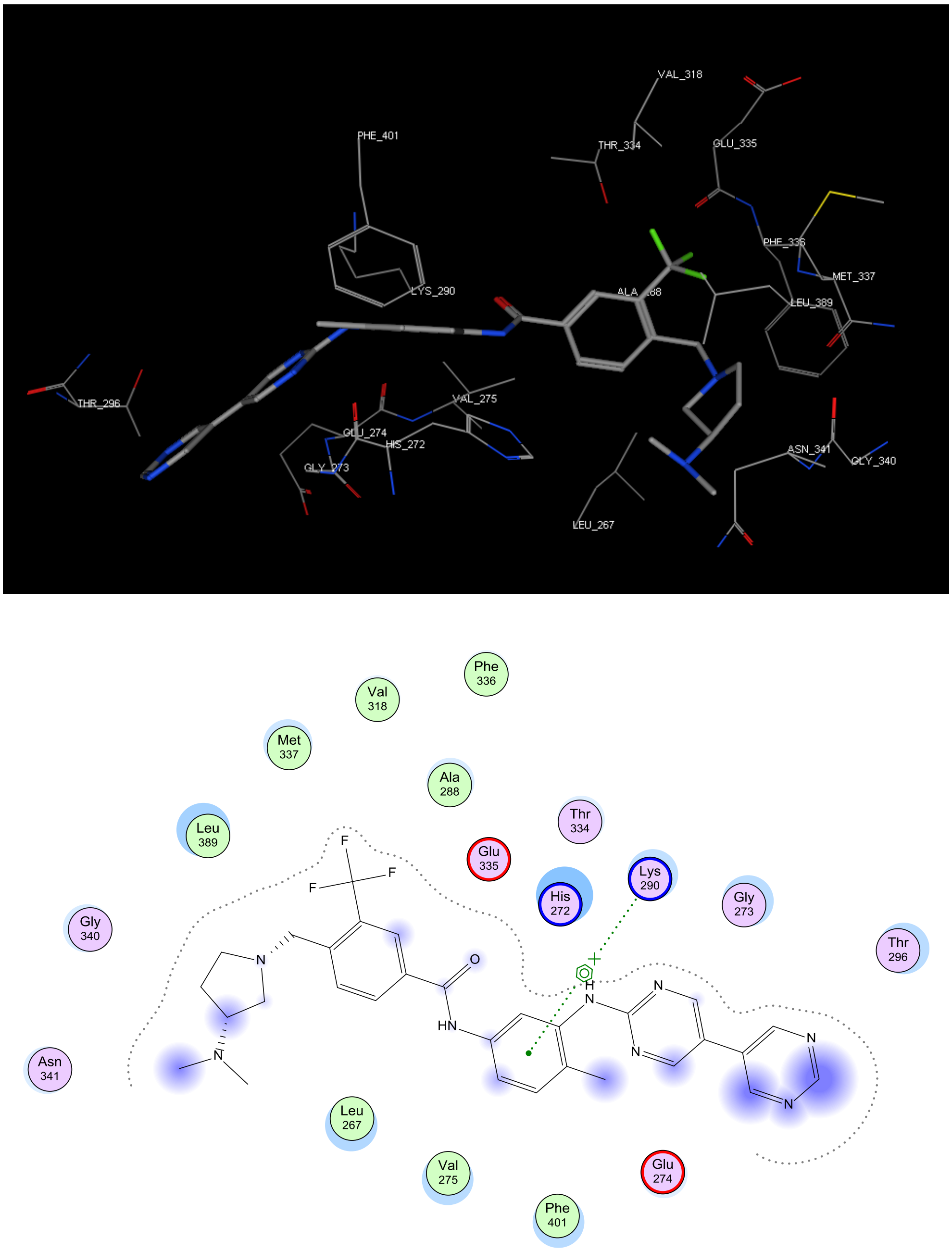

Supplementary Figure S50. Docking of p.(Tyr253His) mutant ABL structure with Bafetinib.

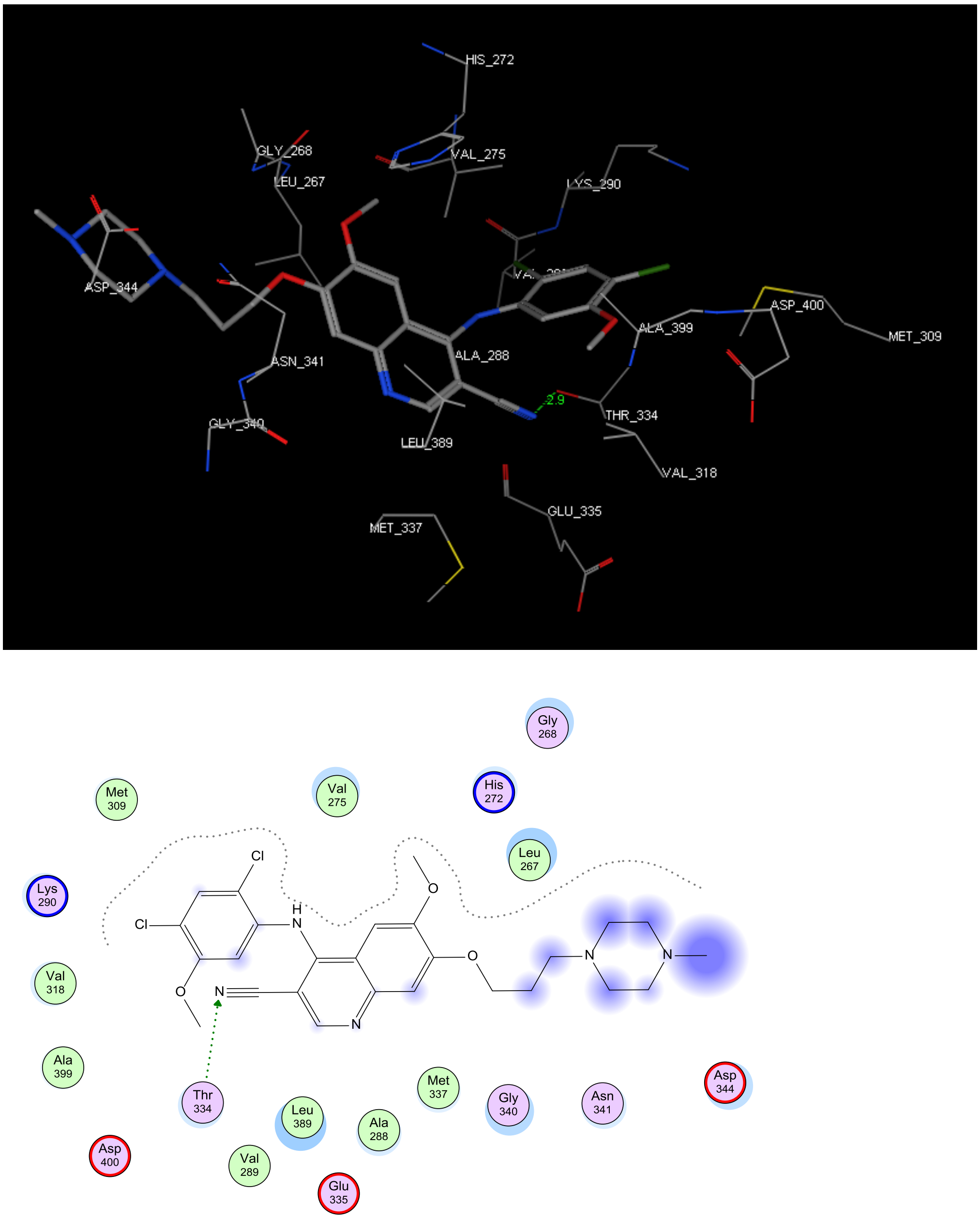

Supplementary Figure S51. Docking of p.(Tyr253His) mutant ABL structure with Bosutinib.

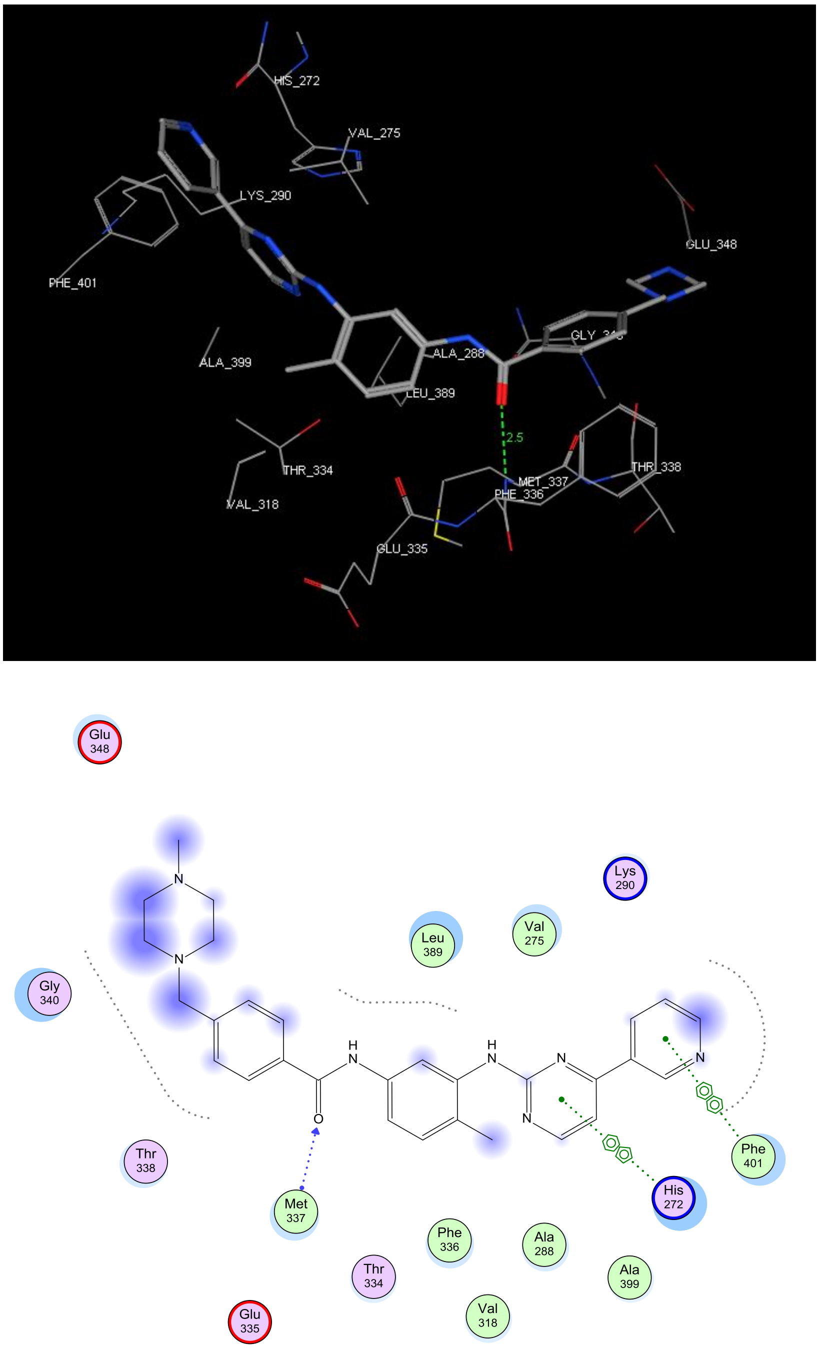

Supplementary Figure S52. Docking of p.(Tyr253His) mutant ABL structure with Imatinib.

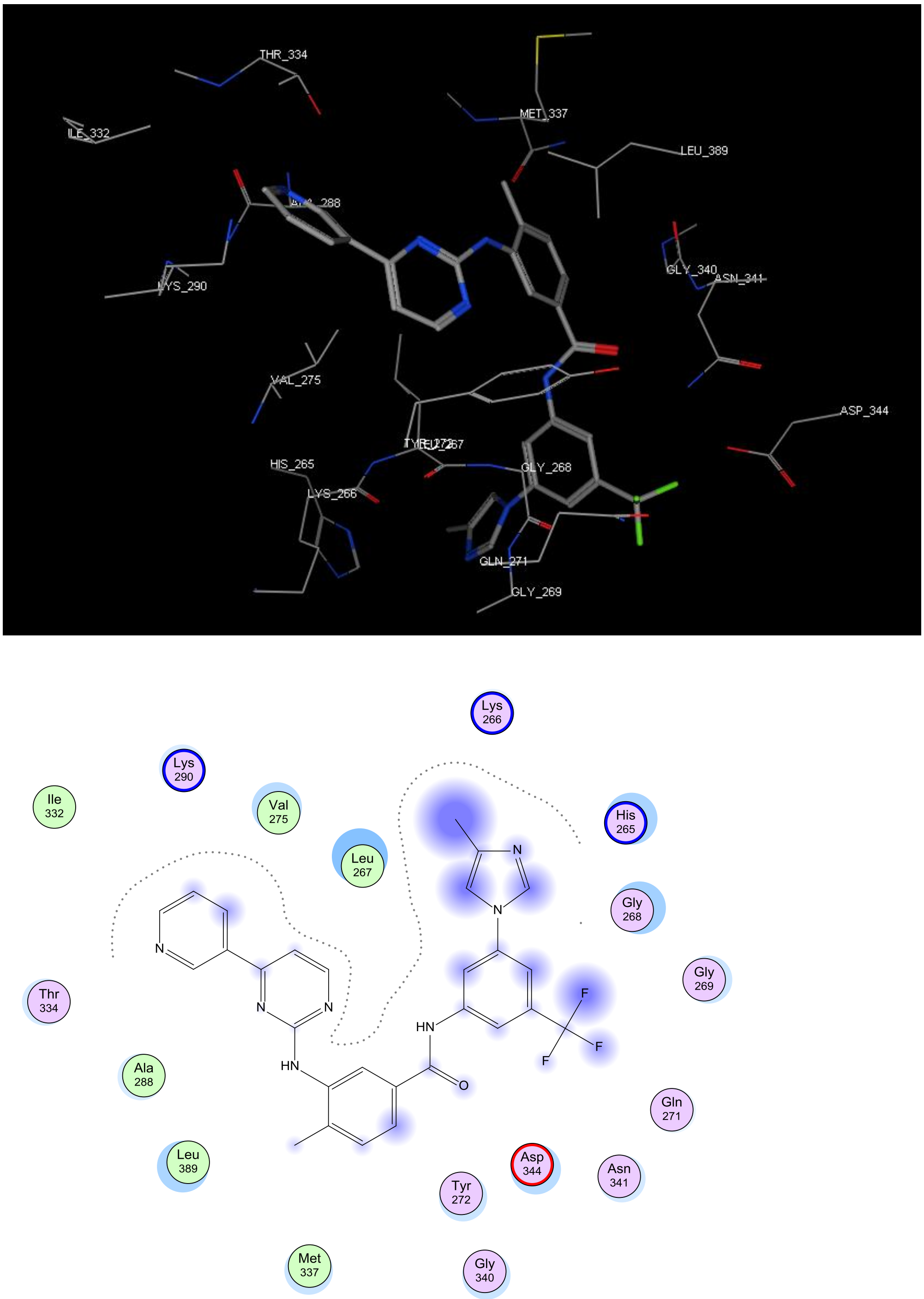

Supplementary Figure S53. Docking of p.(Tyr320His) mutant ABL structure with Nilotinib.

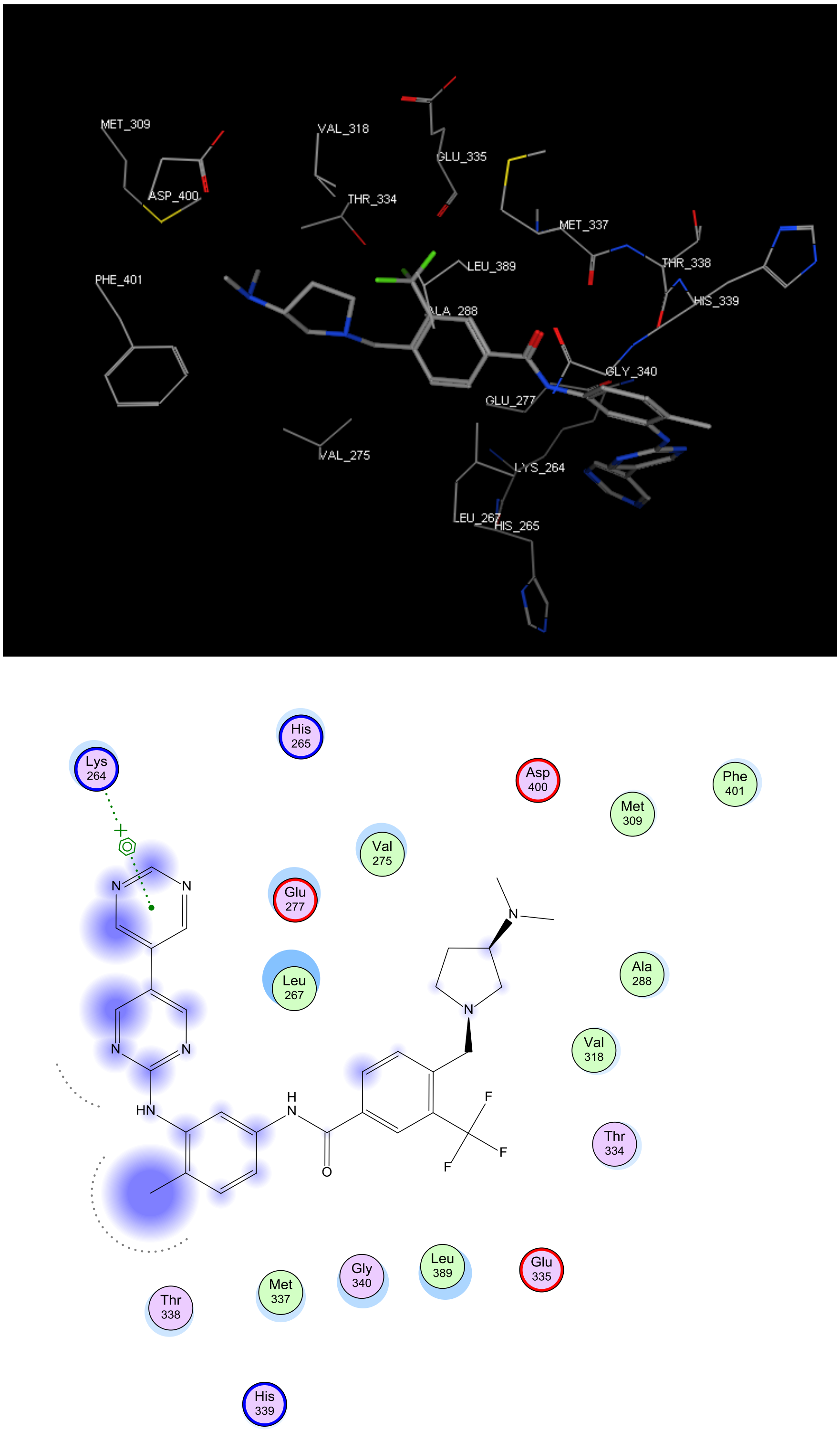

Supplementary Figure S54. Docking of p.(Tyr320His) mutant ABL structure with Bafetinib.

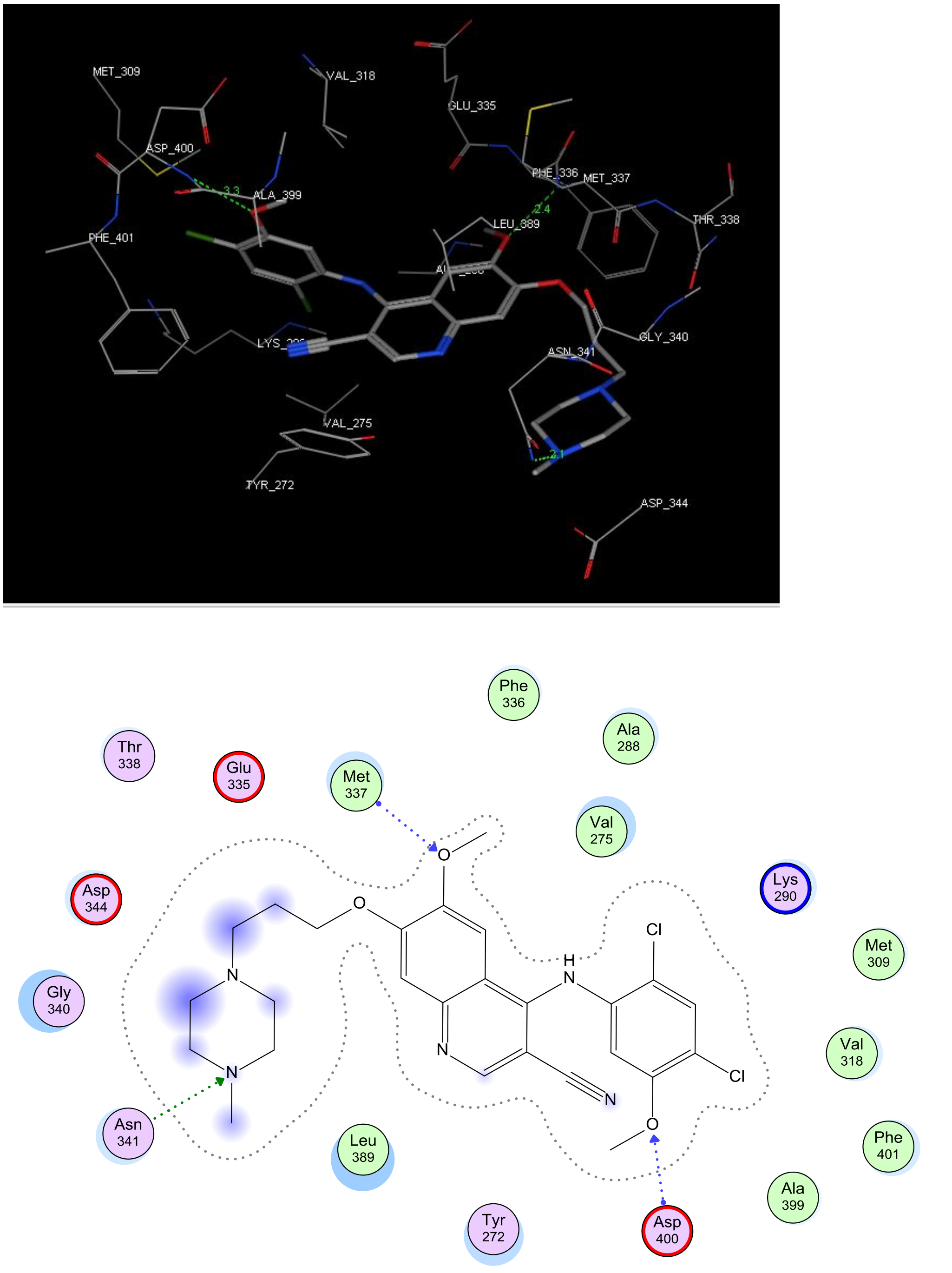

Supplementary Figure S55. Docking of p.(Tyr320His) mutant ABL structure with Bosutinib.

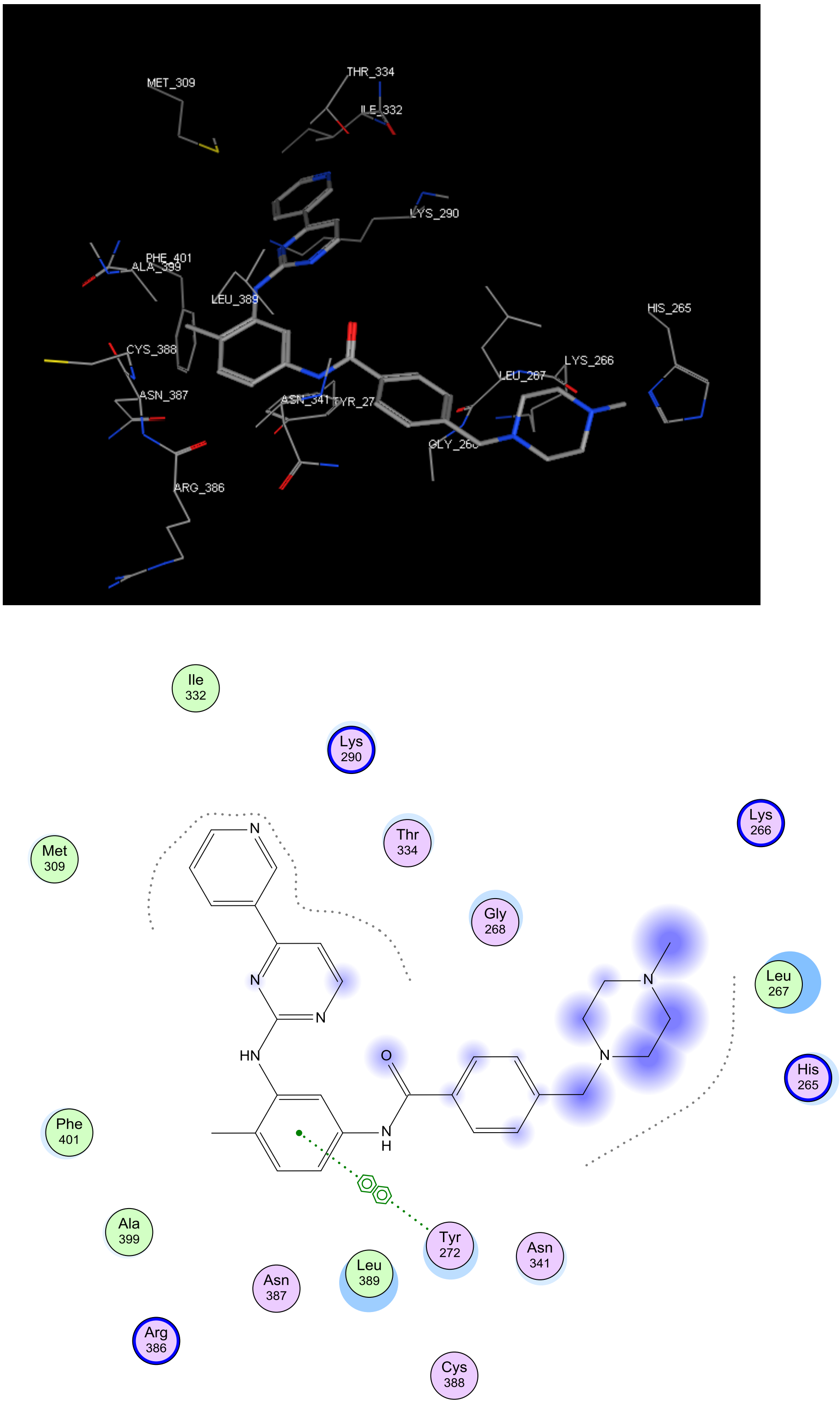

Supplementary Figure S56. Docking of p.(Tyr320His) mutant ABL structure with Imatinib.

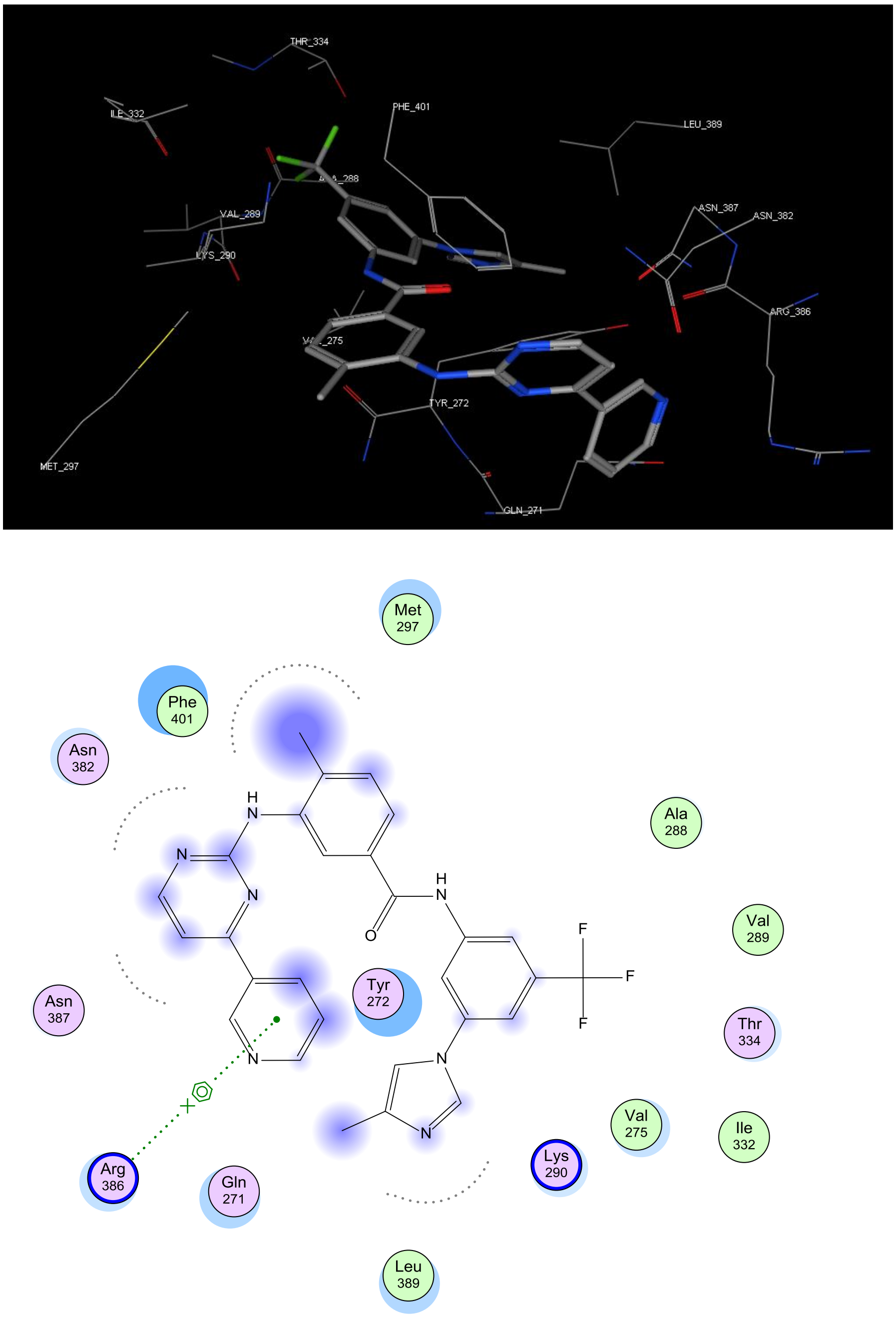

Supplementary Figure S57. Docking of p.(Tyr393*) mutant ABL structure with Nilotinib.

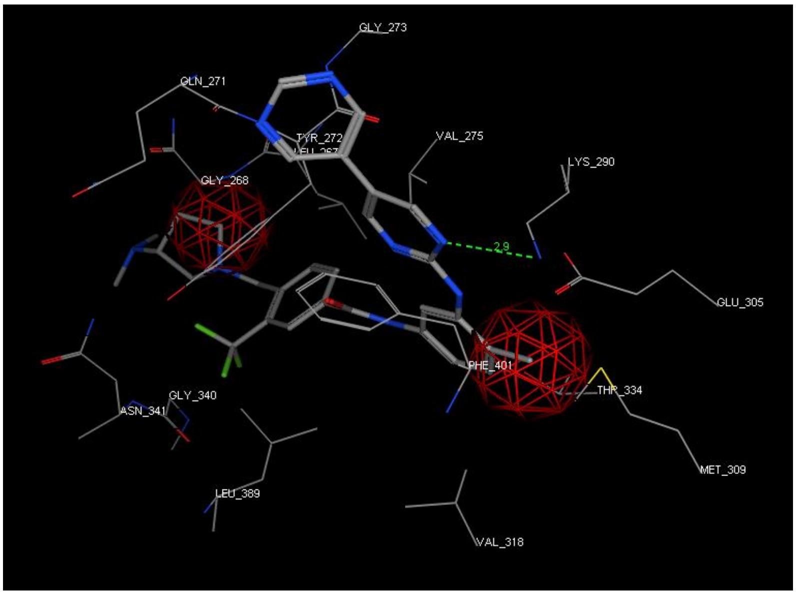

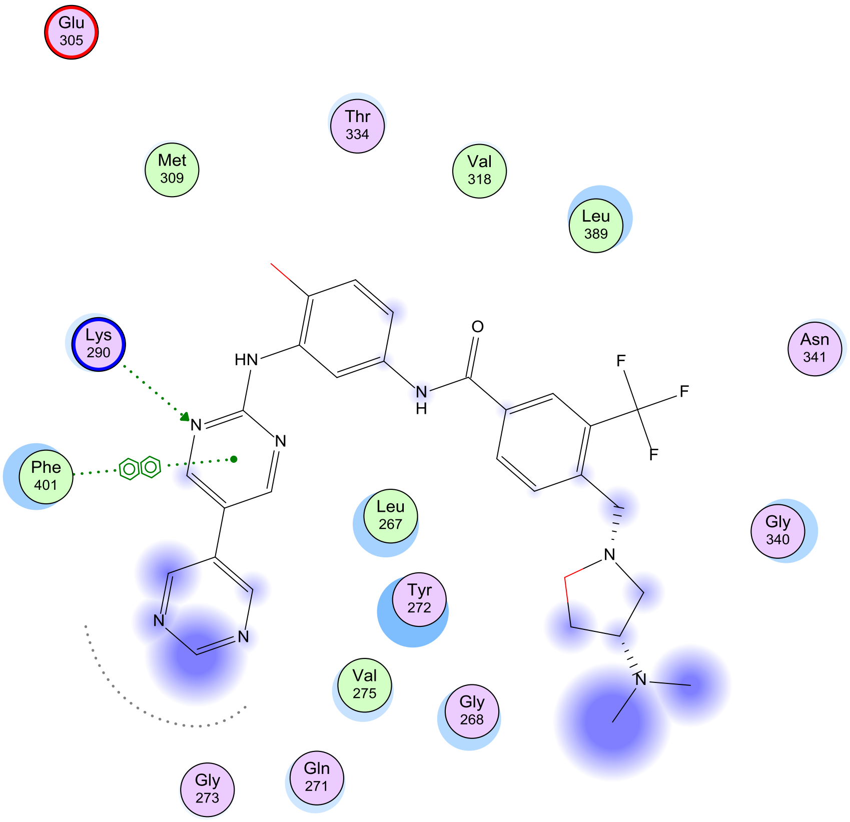

Supplementary Figure S58. Docking of p.(Tyr393*) mutant ABL structure with Bafetinib.

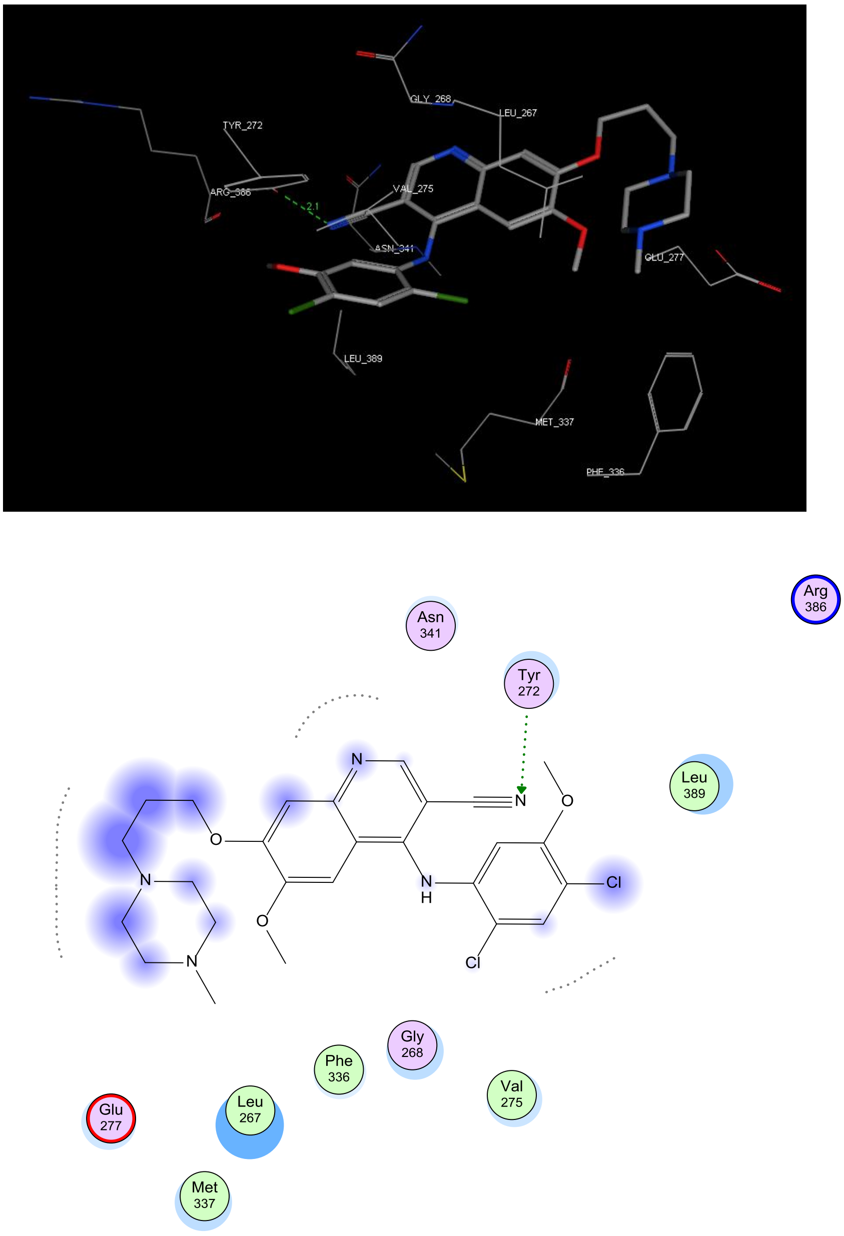

Supplementary Figure S59. Docking of p.(Tyr393*) mutant ABL structure with Bosutinib.

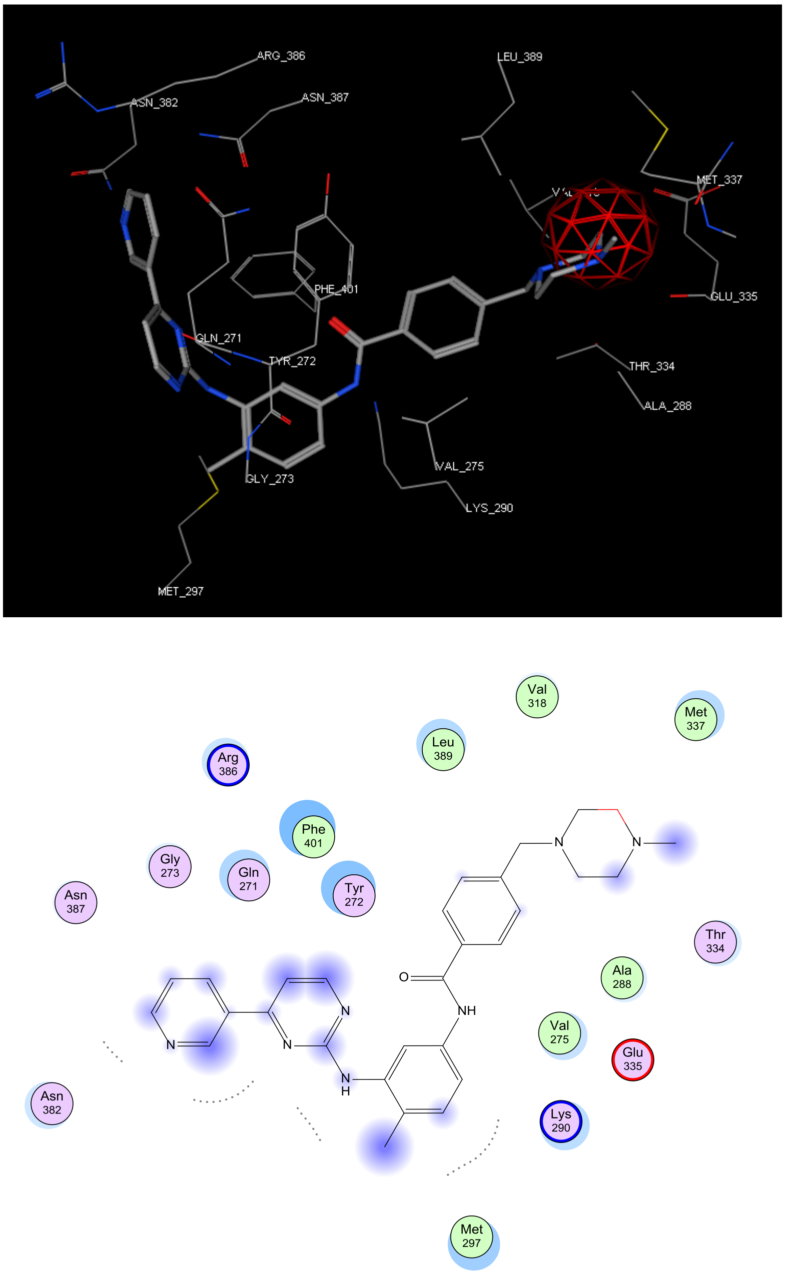

Supplementary Figure S60. Docking of p.(Tyr393*) mutant ABL structure with Imatinib.

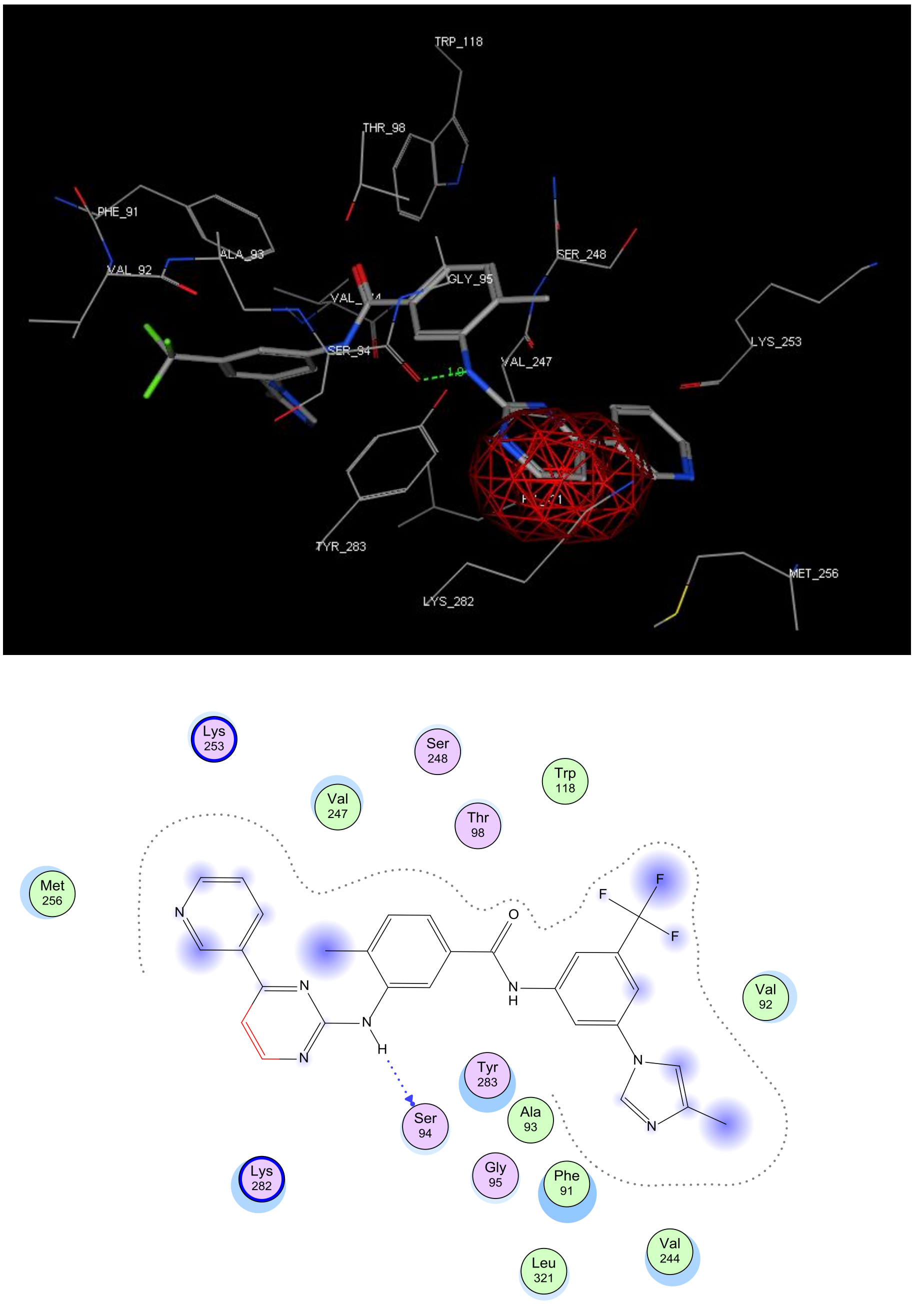

Supplementary Figure S61. Docking of p.(Val256Gly) mutant ABL structure with Nilotinib.

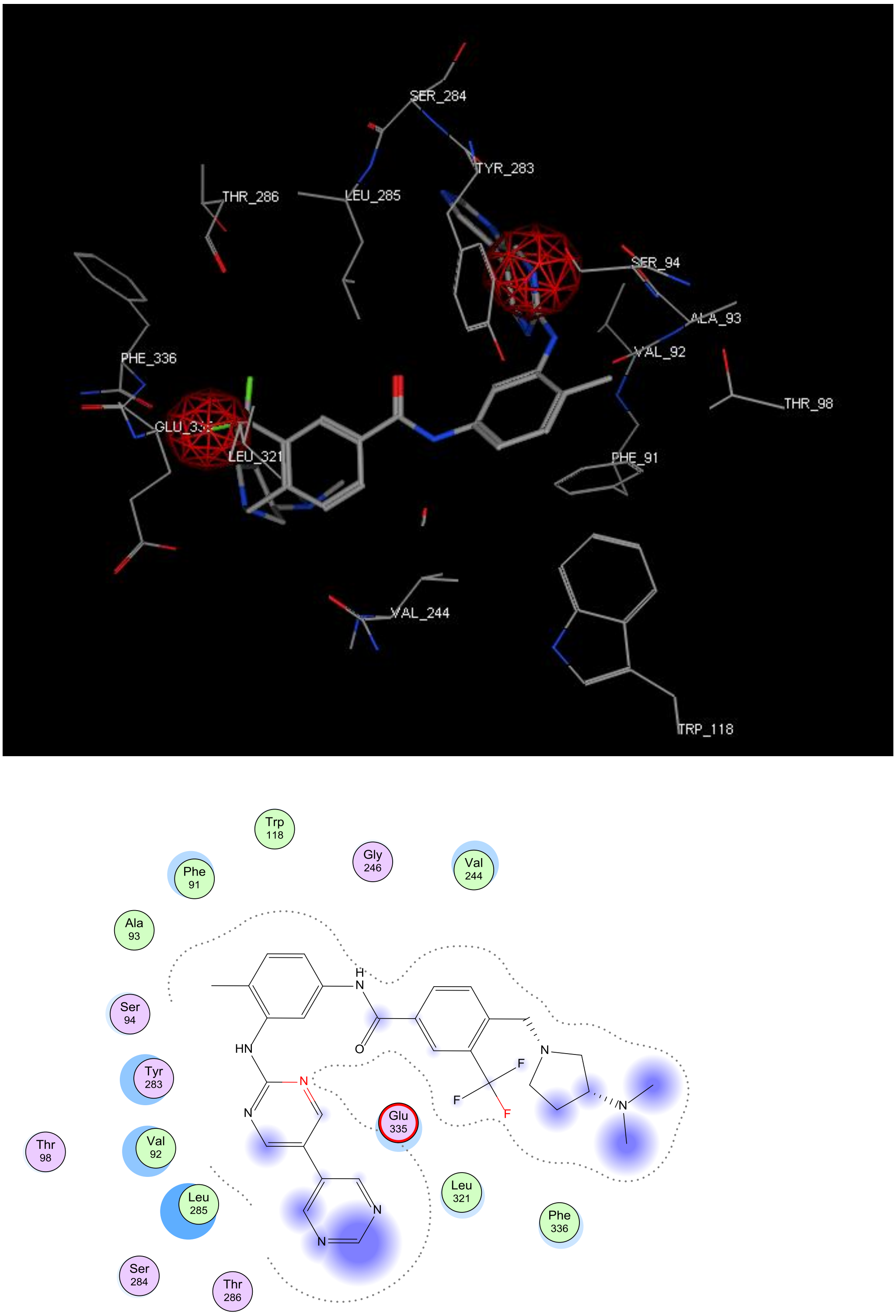

Supplementary Figure S62. Docking of p.(Val256Gly) mutant ABL structure with Bafetinib.

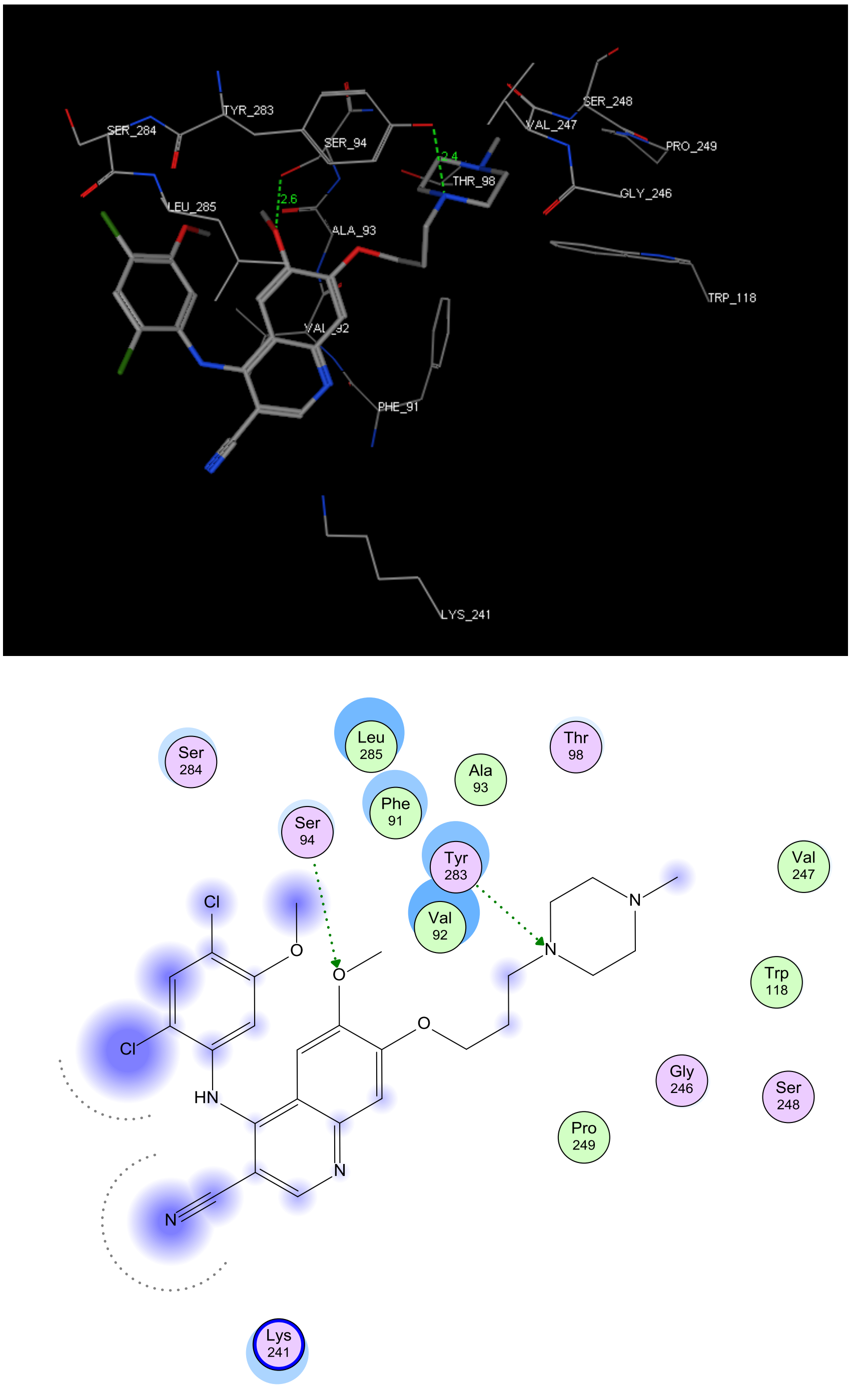

Supplementary Figure S63. Docking of p.(Val256Gly) mutant ABL structure with Bosutinib.

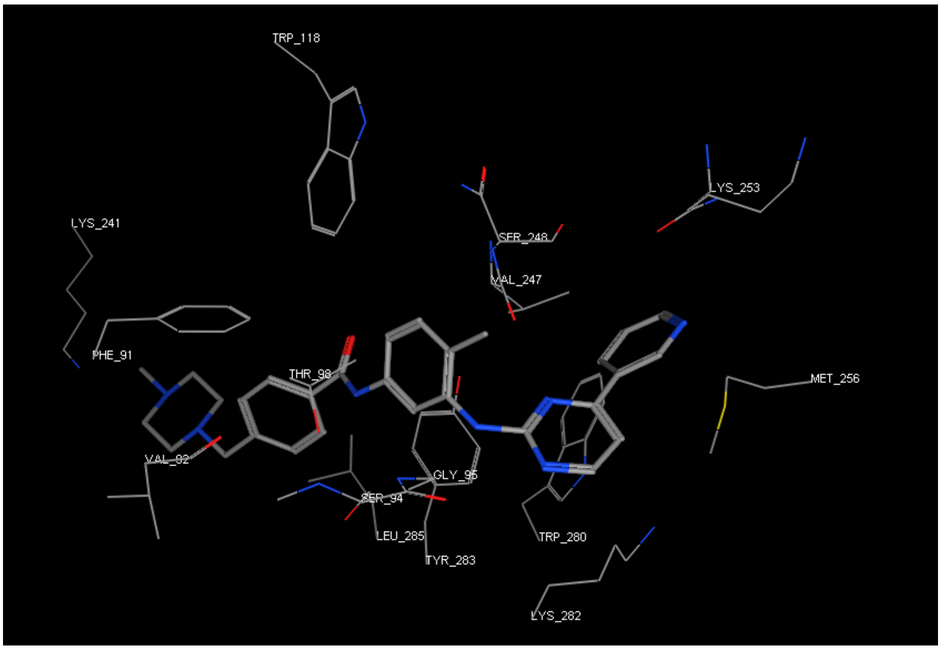

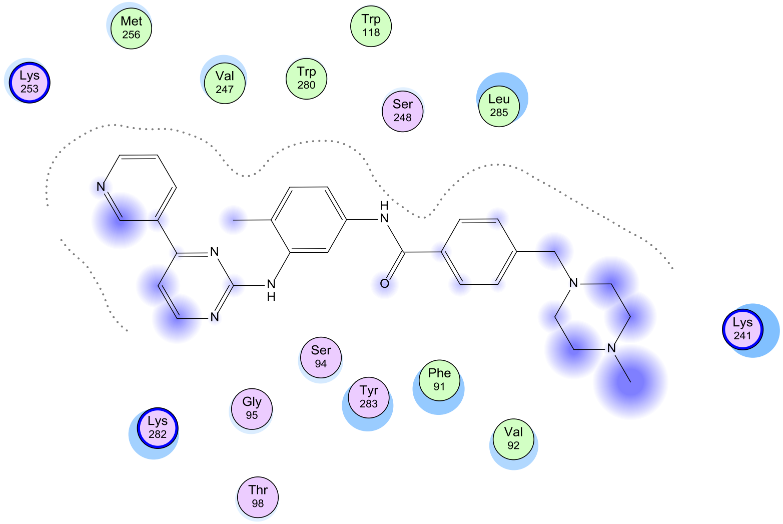

Supplementary Figure S64. Docking of p.(Val256Gly) mutant ABL structure with Imatinib.
